# Global DNA methylation and transcriptional analyses of human ESC-derived cardiomyocytes

**DOI:** 10.1007/s13238-013-0016-x

**Published:** 2014-01-29

**Authors:** Ying Gu, Guang-Hui Liu, Nongluk Plongthongkum, Christopher Benner, Fei Yi, Jing Qu, Keiichiro Suzuki, Jiping Yang, Weiqi Zhang, Mo Li, Nuria Montserrat, Isaac Crespo, Antonio del Sol, Concepcion Rodriguez Esteban, Kun Zhang, Juan Carlos Izpisua Belmonte

**Affiliations:** 1 Gene Expression Laboratory, Salk Institute for Biological Studies, 10010 North Torrey Pines Road, La Jolla, CA 92037 USA; 2 National Laboratory of Biomacromolecules, Institute of Biophysics, Chinese Academy of Sciences, Beijing, 100101 China; 3 State key Laboratory of Drug Research, Shanghai Institute of Materia Medica, Chinese Academy of Sciences, Shanghai, 201203 China; 4 Department of Bioengineering, University of California at San Diego, La Jolla, CA 92093 USA; 5 Center of Regenerative Medicine in Barcelona, Dr. Aiguader 88, 08003 Barcelona, Spain; 6 Biomedical Research Networking Center in Bioengineering, Biomaterials and Nanomedicine (CIBER-BBN), Poeta Mariano Esquillor s/n, 50018 Sargossa, Spain; 7 Luxembourg Centre for Systems Biomedicine (LCSB), University of Luxembourg, Walferdange, Luxembourg

**Keywords:** human cardiomyocyte, DNA methylation, microarray, heart development

## Abstract

With defined culture protocol, human embryonic stem cells (hESCs) are able to generate cardiomyocytes *in vitro*, therefore providing a great model for human heart development, and holding great potential for cardiac disease therapies. In this study, we successfully generated a highly pure population of human cardiomyocytes (hCMs) (>95% cTnT^+^) from hESC line, which enabled us to identify and characterize an hCM-specific signature, at both the gene expression and DNA methylation levels. Gene functional association network and gene-disease network analyses of these hCM-enriched genes provide new insights into the mechanisms of hCM transcriptional regulation, and stand as an informative and rich resource for investigating cardiac gene functions and disease mechanisms. Moreover, we show that cardiac-structural genes and cardiac-transcription factors have distinct epigenetic mechanisms to regulate their gene expression, providing a better understanding of how the epigenetic machinery coordinates to regulate gene expression in different cell types.

**Electronic supplementary material:**

The online version of this article (doi:10.1007/s13238-013-0016-x) contains supplementary material, which is available to authorized users.

## Introduction

Heart failure, caused by massive loss or dysfunction of hCMs, is the main cause of death and morbidity in the developed world. Treatments for this devastating disorder are inefficient and usually focused around symptomatic alleviation, while the main cause of the disease, that is, loss of hCMs and associated contractile function, remains unchallenged. Thus, identification of factors reducing fibrotic scarring or promoting hCM proliferation is of the utmost importance for public health. Achieving this goal is complicated by the difficulty of obtaining large numbers of pure, fully differentiated hCMs and their intermediate stages.

hESCs have the potential to differentiate into cells of all lineages, therefore providing an ideal *in vitro* model to study organ development and disease mechanisms. Recent efforts have successfully developed several protocols, by which hESCs can be differentiated into hCMs (Yang et al., 2008; Kattman et al., 2011; Lian et al., 2012; Willems et al., 2012; Zhang et al., 2012). Furthermore, hESC-derived hCMs have been analyzed by microarray and a group of cardio-specific genes were revealed (Beqqali et al., 2006; Cao et al., 2008; Synnergren et al., 2008). However, one inevitable concern is that by these published differentiation protocols, it is difficult to obtain pure hCMs. Hence, any following study using the mixed cell population may potentially give some misleading results. To overcome this, an improved protocol is needed in order to achieve a satisfactory purity of hESC-derived hCMs. Moreover, while the gene expression profile for hCMs has been provided, a more comprehensive investigation on gene expression together with epigenetic regulation, such as DNA methylation, during the hCM differentiation is still lacking.

In an effort to overcome these limitations, we have developed a new cardiac differentiation protocol starting from hESCs that yields a highly pure population of hCMs (>95%) suitable for genomic studies. As proof-of-concept, and in an effort to uncover new cardiac-specific targets relevant for therapeutic applications, we performed global epigenetic and transcriptional analyses during cardiac specification using this protocol. We performed transcriptional profiling and genome-wide DNA methylation analyses of hCMs and compared them to undifferentiated hCMs and hESC-derived neural stem cells (hNSCs). Our results provide a step forward towards the characterization of hCMs at both the transcriptional and epigenetic levels, and offer a powerful tool towards better understanding heart physiology and disease.

## Results

### Derivation of highly enriched cardiomyocytes from hESCs

**Figure 1:**
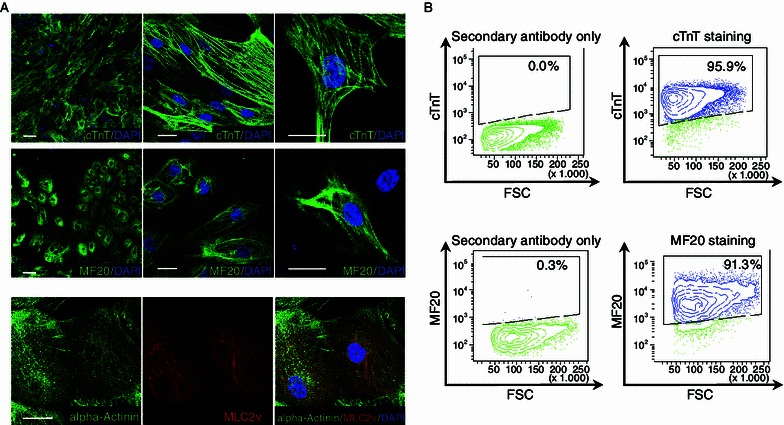
Characterization of hESC-derived hCMs. (A) Immunofluorescence analyses showing the expression of key cardiac markers in d25 hCMs derived from H9 hESCs. Top panel: cTnT (green). Middle panel: MF20 (green). Bottom Panel: alpha-Actinin (green) and MLC-2v (red). Scale bar: 20 μm. (B) Flow cytometry analysis of cells expressing cTnT (top panel) or MF20 (bottom panel). Cells were collected on day 25 of hCM differentiation

Following Palecek’s previous protocol (Lian et al., 2012), hESCs were seeded as single cells on Matrigel and maintained in mTeSR. The GSK3 specific inhibitor CHIR99021 was added on the first day of differentiation, followed by the Wnt inhibitor IWP4 on day 3. After 15 days, a relatively pure and contracting cardiomyocyte population was obtained (Movie S1). We enriched this fraction by collecting and washing the contracting hCM sheets and re-plating them on fresh Matrigel plates (Movies S2 and S3). These subcultured hCMs expressed the CM-specific markers cardiac troponin T (cTnT) and sarcomeric myosin (MF20), and exhibited normal cardiac sarcomere organization, as indicated by alpha-Actinin and MLC2v co-staining (Fig. 1A). Flow cytometry analysis indicated a majority of definitive cardiac cells were present at day 25 (~96% cTnT^+^ cells and ~91% MF20^+^, Fig. 1B).

### Global gene expression profiling in hCMs

**Figure 2:**
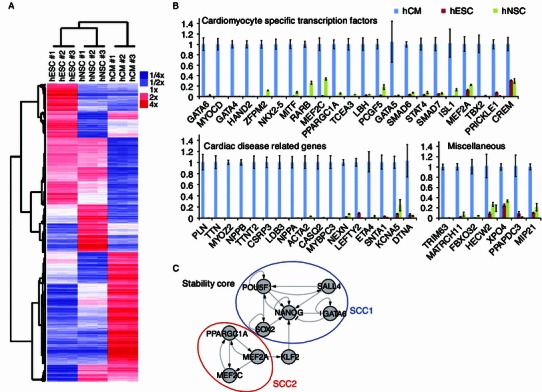
Global gene expression profiling of hCMs. (A) Heatmap and hierarchical clustering analysis of gene expression profiles of hESC, hNSCs, and hCMs performed in triplicate. Color represents the expression level relative to mean. (B) RT-qPCR analysis of transcript expression in hESCs, hCMs, and hNSCs. The expression levels of genes in hCMs were set to one. Data are shown as mean ± s.d., *n* = 3. (C) The gene regulation network that includes a stability core of 9 genes distributed in two strongly connected components (SCCs). These two SCCs or clusters of circuits, named as SCC1 and SCC2 could be broadly linked to pluripotency cellular features and the hCM-specific properties, respectively. Nodes represent genes and edges regulatory interactions positive (“→”) and negative (“—|”)

We obtained RNA from undifferentiated hESCs and hCMs and used it for microarray analysis. hESC-derived hNSCs (Liu et al., 2012) were used as a control population. All the cells shared the same genetic background (H9), allowing for an unbiased side-by-side comparison of their gene expression profile. Three biological replicates from each cell type were measured with PrimeView Human Gene Expression Arrays, covering more than 36,000 transcripts and variants. All of the replicates were highly reproducible, supporting the purity and reliability of the method. Clustering data indicated that hESCs and hNSCs were closer to each other in terms of expression, while hCMs showed a more distinct expression pattern (Fig. 2A). Among represented transcripts, we identified 695 genes that showed at least a two-fold up-regulation and 401 genes that showed at least a two-fold down-regulation in hCMs compared to both hESCs and hNSCs (Tables S1 and S2). A group of the cardiac-enriched genes were validated by qRT-PCR (Fig. 2B).

To gain further insight into the functions of these hCM differentially expressed genes, we performed Gene Ontology (GO) analyses using the BiNGO (Maere et al., 2005) Cytoscape (Shannon et al., 2003) plugin. Interestingly, hCM up-regulated genes were significantly over-represented in cardiac function-related GO terms (complete lists of GO terms are shown in Tables S3–5), including muscle contraction, heart development, and sarcomeric structures. In contrast, hCM differentially down-regulated genes were significantly clustered into GO terms such as M phase, nuclear division, and mitosis (complete lists of GO terms are shown in Tables S6–8), suggesting that mitosis in hCMs is strongly repressed, as has been consistently observed in hCMs during maturation (Zhang et al., 2012).

Next, we analyzed differentially regulated targets in the context of gene regulatory networks. We could identify the minimal combinations of reprogramming determinants responsible for the transition of hESCs towards hCMs. Specifically, our computational model defined a gene regulatory network stability core with two major components associated with both pluripotency and hCMs. Perturbation of these genes (up- or down-regulation, depending on the original state) triggered a regulatory chain reaction resulting on the transition of hESCs to hCMs (Fig. 2C).

### Interaction network and gene-disease network analyses of hCM-enriched genes

**Figure 3:**
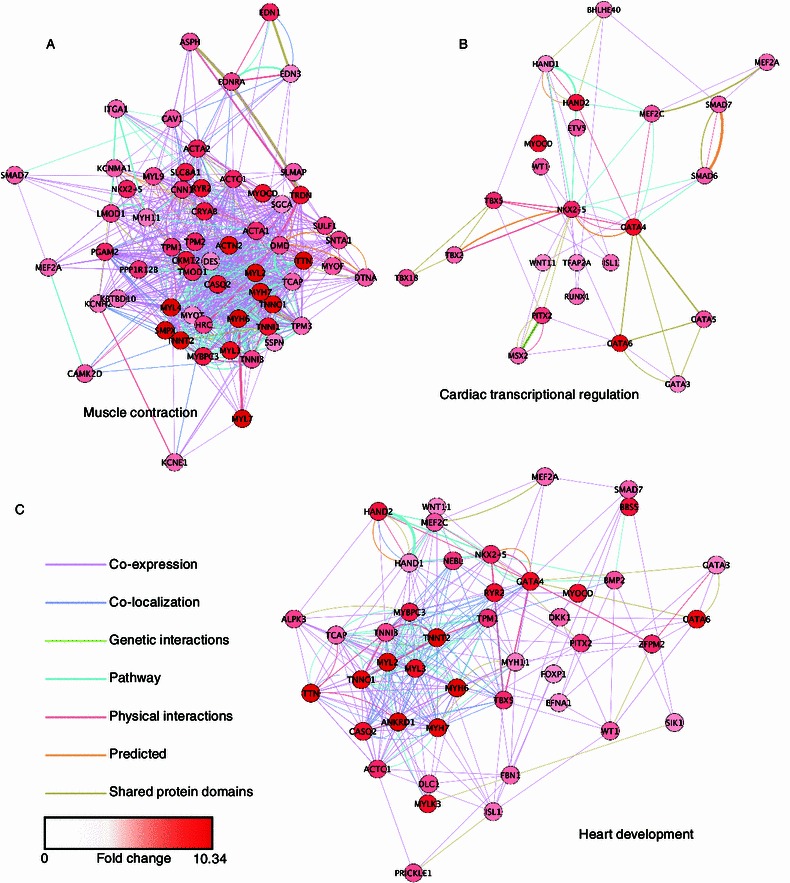
Association networks of hCM-enriched genes involved in function groups of muscle contraction, heart development, and cardiac transcriptional regulation. Networks were generated by GeneMANIA Cytoscape plugin. Nodes represent genes involved in the selected function group, and edges represent the relationships between linked genes. Node colors represent the average expression fold changes in hCMs compared to both hESCs and hNSCs

To better understand the functional interaction between the identified hCM-specific genes, all of the 695 hCM-enriched genes were screened into GeneMANIA (Montojo et al., 2010) Cytoscape plugin to produce a functional association network based on their relationships, such as co-expression, co-localization, genetic interaction, and physical interaction (a complete information of the interactions is shown in Table S9). Subnetworks of functional associations between genes involved in muscle contraction, heart development and cardiac transcriptional regulation were also generated (Fig. 3, complete information of interactions are shown in Tables S10–12). These networks may help in describing new relationships and provide a systematic resource for cardiac gene function prediction.

**Figure 4:**
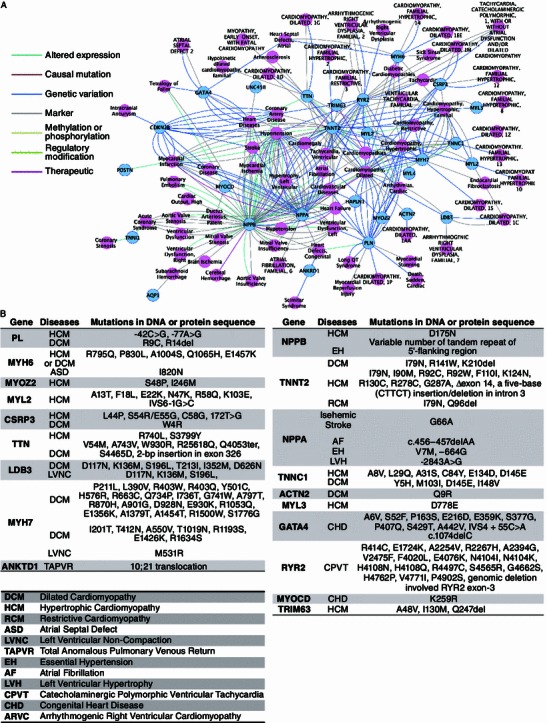
Disease associations of hCM-enriched genes. (A) Gene-disease network of hCM-enriched genes and cardiovascular diseases. Networks were generated by DisGeNET Cytoscape plugin. Nodes represent genes that showed association with cardiovascular diseases, and edges represent the relationship between linked genes and diseases. (B) List of cardiovascular disease-associated mutations in the top hCM-enriched genes. Among the top 50 expressed genes, the ones with known disease-related mutations were listed

Phenotype-genotype relationship studies facilitate the understanding of the normal and diseased physiology. We used the DisGeNET (Bauer-Mehren et al., 2010) plugin of Cytoscape to generate a gene-disease network of highly expressed hCM-specific genes. Of the top 50 up-regulated hCM-specific genes we analyzed, 27 of them (54%) showed associations with at least one known cardiovascular diseases and 5 of them (NPPB, TNNT2, NPPA, RYR2, and PLN) were linked to more than 10 different types of cardiovascular diseases (Fig. 4A, Table S13). In addition, a total number of 142 disease-associated mutations were found in those top hCM-enriched genes (mutations listed in Fig. 4B, references listed in Table S14), suggesting a preferred enrichment of disease causing mutations in hCM-specific genes responsible for critical human heart functions.

### Genome-wide DNA methylation analysis of hCMs

**Figure 5:**
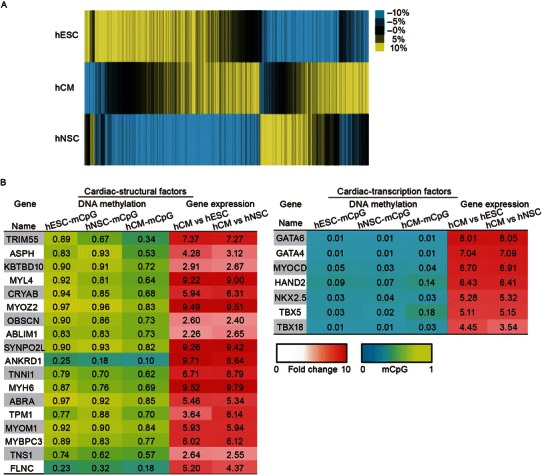
Genome-wide DNA methylation analysis of hCMs. (A) Heatmap and hierarchical clustering of CpG methylation levels measured within 1 kb of promoter regions. Color represents the mCpG/CpG level relative to mean. (B) List of cardiac-structural factors and cardiac-transcription factors with their mCpG levels and relative fold changes in their gene expression

Up to date, relatively few studies have analyzed global DNA methylation status in human cardiac lineages. hESCs, hCMs, and hNSCs were collected for genome-wide DNA methylation profiling using methylation sequencing with bisulfite padlock probes as previously described (Diep et al., 2012). hCMs displayed the highest global DNA methylation level (Fig. 5A) and 985 genes showed an increase in their promoter mCpG levels (5% or more), whereas 195 genes exhibited a 5% or more decrease of promoter DNA methylation compared to hESCs and hNSCs (Tables S15 and S16). Interestingly, these hCM-specific demethylated genes were significantly over-represented in our cardiac-related GO terms search, suggesting that promoter DNA demethylation may contribute to the regulation of cardiac functions (Tables S17–19). Since DNA methylation represents an essential epigenetic mechanism for gene repression, we speculated whether decreased DNA methylation correlated with the increased expression in cardiac-related genes. We combined DNA methylation and microarray data together and identified a group of 29 genes that exhibited hCM-specific promoter demethylation, as well as at least a two-fold up-regulation in gene expression (Table S20). These genes were highly enriched in cardiac-structural related GO terms such as muscle contraction, structural muscle constituents, and sarcomere-related transcripts (Tables S21–23). Again, this data supported the notion that genes encoding cardiac-structural proteins are largely regulated by DNA methylation in hCMs. However, and since the majority of demethylated genes did not show significant changes in gene expression, other layers of regulation, such as histone modifications, might also play a significant role (Xie et al., 2013).

Among those cardiac-structural genes that were specifically demethylated in hCMs, a majority of them exhibited hypermethylated mCpG (>70%) at the promoter regions in hESC and hNSCs (Fig. 5B), indicating that DNA methylation in the promoter region of these genes may function as a major repressive signal in non-cardiac lineages. In contrast, cardiac-specific transcription factors (TFs), such as NKX2.5, GATA6, GATA4, MYOCD, HAND2, TBX5, and TBX18, were more expressed in hCMs than in hESCs or hNSCs, while displaying a similar pattern of low promoter mCpG levels (<10%) in all three lineages, suggesting that the regulation of the expression of cardiac-specific TFs is largely independent of DNA methylation (Fig. 5B).

## Discussion

In this study, we report on an improved protocol for the generation of hCMs from hESCs, which enabled us to identify and characterize a hCM-specific signature, at both the gene expression and DNA methylation levels. The interaction networks we present in this study describe the precise mutual relationships between every hCM-enriched genes, as well as the critical genes involved in the fundamental heart functions, therefore providing an informative and systematic resource for gene function prediction in cardiac research field. In regard to understanding disease mechanisms, we identified a tight correlation between various heart diseases and hCM highly up-regulated genes. These findings not only highlight the importance of these cardiac-specific genes in maintaining normal heart development and functions, but may also provide novel gene targets for uncovering heart disease mechanisms.

By combining the promoter DNA methylation and gene expression profile, we got an overall glance at the gene regulation by DNA methylation in hCMs. Interestingly, we found that a group of cardiac-structural genes exhibited hypermethylated mCpG at the promoter regions in hESCs and hNSCs, but were demethylated in hCMs, suggesting that promoter DNA methylation may be employed as a major transcription repression mechanism in these genes. Supporting this notion, cardiac-structural genes were minimally expressed and often lacked another essential repressive mark, H3K27me3, in human ESCs and early stage cells of multiple non-cardiac lineages (Paige et al., 2012; Xie et al., 2013). In contrast, a group of cardiac TFs display similar low promoter mCpG levels in all three linages we tested. These observations are also in line with the idea that these developmental-related genes are more likely to locate in large genomic domains devoid of DNA methylation in most lineages (Xie et al., 2013). Interestingly, these cardiac-specific TFs were previously found to have high levels of the repressive histone mark H3K27me3 in hESCs and many other non-cardiac lineages. However, during hESC differentiation towards hCM, the levels of H3K27me3 were decreased in hCM specific TFs, highlighting this mark as a major repressing factor in non-cardiac cell types (Paige et al., 2012; Xie et al., 2013). Whether DNA methylation offers a more stable regulation for cardiac-structural genes expression and whether the highly flexible nature of histone modifications is required for the dynamic cardiac-TF expression in a time-sensitive manner will be interesting questions to ask in future studies.

Overall, our analysis provides new insights into the mechanisms of hCM transcriptional regulation, and stands as an informative and rich resource for investigating cardiac gene functions, as well as providing a better understanding of how the epigenetic machinery coordinates to regulate gene expression in different cell types. Furthermore, the cardiac-specific molecular hallmarks and epigenetic signatures presented in this study might be of relevance for clinical applications as biomarkers for diagnosis and treatment of heart-related diseases.

## Materials and methods

### Cell culture and differentiation

H9 hESCs were purchased from WiCell Research, and cultured under standard hESC culture conditions as previously described (Liu et al., 2011). Differentiation of H9 hESC into hNSC was followed by the protocol described previously (Liu et al., 2012).

The hCM derivation from H9 hESCs was performed as previously described (Lian et al., 2012), with important modification to further improve the purity. Briefly, single cell suspension of H9 hESCs were seeded onto Matrigel (BD Biosciences) pre-coated cell culture dishes at a density of 100,000 cells per cm^2^ in mTeSR (StemCell Technologies) in the presence of ROCK inhibitor Y-27632 (Sigma-Aldrich) for 24 h. Cells were then cultured in mTeSR for another 2 days. Differentiation was initiated by treatment with 12 μmol/L CHIR99021 (Selleck) in RPMI/B27-insulin (Life Technologies) for 24 h (day 0 to day 1), and medium was then changed to RPMI/B27-insulin. On day 3, 5 μmol/L Wnt inhibitor IWP4 (Stemgent) was added into RPMI/B27-insulin and cells were cultured without medium change for 48 h. From day 5, cells were maintained in RPMI/B27 with medium change every 2–3 days. On day 15, contracting hCM sheets or clusters were collected by intensive mechanic washing or manually picking, re-plated onto fresh Matrigel pre-coated dishes and maintained in RPMI/B27.

### Immunofluorescence

Cells were fixed with 4% paraformaldehyde, permeabilized in 0.3% Triton X-100/PBS and incubated with primary antibodies overnight at 4°C at following dilutions: mouse anti-cTnT (1:200, Lab Vision), mouse anti-MF-20 (1:20 Developmental Studies Hybridoma Bank), rabbit anti-MLC2v (1:200, ProteinTech Group), mouse anti-alpha-Actinin (1:200, Sigma-Aldrich). Cells were then washed and incubated with Alex Fluor 488 goat anti-mouse IgG (1:500, Life Technologies) and/or Alex Fluor 568 goat anti-rabbit IgG (1:500, Life Technologies) for 1 h at RT. Cell nuclei were counter stained with DAPI (1 μg/mL, Sigma-Aldrich). Images were captured using a Zeiss LSM780 confocal microscope, and were analyzed in ZEN 2011 software.

### Flow cytometry

Flow cytometry analyses were performed as described previously (Zhang et al., 2012), with antibody concentrations as following: mouse anti-cTnT (1:200, Lab Vision), mouse anti-MF-20 (1:20 Developmental Studies Hybridoma Bank), and Alex Fluor 488 goat anti-mouse IgG (1:500, Life Technologies). Data were collected and analyzed on a LSRII flow cytometer (Becton-Dickinson).

### Microarray analysis

hESC, hNSC, and hCM samples were prepared in biological triplicates. Total RNA was extracted using Trizol Reagent (Life Technologies) and further purified by RNeasy Mini Kit (Qiagen). Microarray experiments were performed using Affymetrix GeneChip PrimeView Human Gene Expression Arrays in the Functional Genomics Core Facility at the Salk Institute for Biological Studies according to the manufacturer’s protocol (Affymetrix, Santa Clara, CA). Expression signals were scanned on Affymetrix GeneChip Scanner 3000 7G. Gene expression values were calculated and normalized with RMA using R/Bioconductor. Expression levels were clustered using Cluster 3.0 and visualized using Java TreeView. All data have been deposited in NCBI’s Gene Expression Omnibus and are accessible through GEO Series accession number GSE48257. http://www.ncbi.nlm.nih.gov/geo/query/acc.cgi?token=fxqpxgigmoaggzg&acc=GSE48257

### Quantitative RT-PCR

RT-qPCR was performed as described previously (Liu et al., 2011). The primer sequences were listed in Table S24.

### Genome-wide DNA methylation analysis

Bisulfite padlock probe sequencing was performed as previously described (Diep et al., 2012). Briefly, genomic DNA was extracted from H9-hESC, H9-hNSC, and H9-hCM using QIAamp DNA Micro Kit (QIAGEN), and approximately 1000 ng of genomic DNA was bisulfite converted with EZ-96 DNA Methylation-Lightning MagPrep kit (Zymo Research). Approximately 250 ng of bisulfite converted genomic DNAs were mixed with normalized amount of the genome-wide scale padlock probe set. The annealed padlock probes were polymerized, gap-filled, and ligated to generate circularized DNA. The circularized captured targets were amplified and barcoded by PCR using the library-free BSPP protocol as previously described (Diep et al., 2012). The resulting bisulfite sequencing libraries were pooled in the same molar ratio, size-selected at the fragment size approximately 375 bp in 6% TBE PAGE gel (Life Technologies), and sequenced by Illumina HiSeq2000 sequencer (110 bp, paired-end reads). The bisulfite reads were mapped to the *in silico* bisulfite-converted human genome sequences (hg19) by bisREADMapper (Diep et al., 2012). DNA methylation frequency (at level from 0–1) at each CpG site with minimum 10× depth coverage was calculated. Only CpG sites with 10× coverage in each cell type were used for differential analysis. Promoter CpG methylation levels were calculated by averaging the mCpG/CpG ratio for all CpG dinucleotides with 10× coverage within 1 kb of the TSS. Clustering of promoter methylation ratios was carried out using Cluster 3.0 and visualized using Java TreeView.

### Gene Ontology (GO) analysis

Functional properties of the differentially expressed or methylated genes were categorized using the BiNGO Cytoscape plugin as described previously (Maere et al., 2005). A *P*-value of less than 0.05 was considered statistically significant.

### Gene regulatory networks reconstruction

Differentially expressed genes obtained after the performance of a *t*-test with a *P*-value < 0.05 were connected to expression regulatory interactions from literature. For this specific purpose we use the information contained in the ResNet mammalian database from Ariadne Genomics (http://www.ariadnegenomics.com/) (Novichkova et al., 2003; Daraselia et al., 2004). We selected only the interactions included in the category of Promoter Binding and Direct Regulation. In order to contextualize the network to the biological conditions under which the expression data was obtained we applied an algorithm that exploits the consistency between predicted and known stable states from experimental data to guide an iterative network pruning. The algorithm predicted missing expression values in gene regulatory networks, and could be applied to contextualize the network when all the expression values in two attractors are known. The method assumes a Boolean model to compute attractors of networks that are iteratively pruned by means of an evolutionary algorithm. The evolutionary algorithm samples the probability distribution of positive circuits and individual interactions within the subpopulation of the best-pruned networks at each iteration. The resulting contextualized network is based not only on previous knowledge about local connectivity but also on a global network property (stability).

Attractor computation was performed assuming a Boolean model applying a synchronous updating scheme (Garg et al., 2008) that updates all gene states simultaneously at each step until the system reaches an attractor. For this purpose we used our own implementation (Crespo et al., 2013) written in Perl of the algorithm described by Garg and co-workers (Garg et al., 2007). We implemented the Johnsons algorithm (Johnson, 1975) to detect all elementary circuits in the network. Both elementary circuit detection and positive circuits sorting scripts were implemented in Perl.

### Gene functional association network analysis

Networks of gene associations (co-expression, co-localization, genetic interaction, pathway, and more) were generated using the GeneMANIA Cytoscape plugin as described previously (Montojo et al., 2010). The “query gene based” weighting method was used in this study. Sub-networks were generated by selecting genes that were categorized in the same functional GO group.

### Gene-disease association network analysis

Network of gene-disease associations (altered expression, casual mutation, genetic variation, marker, and more) were generated using DisGeNET Cytoscape plugin as described previously (Bauer-Mehren et al., 2010). The top 50 hCM up-regulated genes were inquired individually for disease association in the cardiovascular disease class. All the individual gene-disease networks were then combined based on the shared disease type.

## Electronic supplementary material

Below is the link to the electronic supplementary material.

Movie S1: hCMs on day 15 of differentiation on Matrigel dish. Movie was made under Olympus IX51 inverted microscope with 4x objective

Movie S2: Subcultured hCMs on day 25 of differentiation on Matrigel dish. Movie was made under Olympus IX51 inverted microscope with 10x objective

Movie S3: Subcultured hCMs on day 25 of differentiation on Matrigel dish. Movie was made under Olympus IX51 inverted microscope with 20x objective

Table S1: List of genes differentially up-regulated in hCMs compared to hESCs and hNSCs. This file contains the list of 695 genes that showed at least a two-fold increaseof transcript expression in hCMs compared to hESCs and hNSCs

Table S2: List of genes differentially down-regulated in hCMs compared to hESCs and hNSCs. This file contains the list of 401 genes that showed at least a two-fold decrease of transcript expression in hCMs compared to hESCs and hNSCs

Table S3: GO analysis of hCM differentially up-regulated genes-Biological Process. This file contains the list of GO terms in the category of biological process that were significantly (p<0.05) overrepresented among the 695 hCM differentially up-regulatedgenes

Table S4: GO analysis of hCM differentially up-regulated genes-Molecular Function.This file contains the list of GO terms in the category of molecular function that were significantly (p<0.05) overrepresented among the 695 hCM differentially up-regulatedgenes

Table S5: GO analysis of hCM differentially up-regulated genes-Cellular Component. This file contains the list of GO terms in the category of cellular component that were significantly (p<0.05) overrepresented among the 695 hCM differentially upregulated genes

Table S6: GO analysis of hCM differentially down-regulated genes-Biological Process. This file contains the list of GO terms in the category of biological process that were significantly (p<0.05) overrepresented among the 401 hCM differentially downregulatedgenes

Table S7: GO analysis of hCM differentially down-regulated genes-Molecular Function. This file contains the list of GO terms in the category of molecular function that were significantly (p<0.05) overrepresented among the 401 hCM differentiallydown-regulated genes

Table S8: GO analysis of hCM differentially down-regulated genes-Cellular Component. This file contains the list of GO terms in the category of cellular component that were significantly (p<0.05) overrepresented among the 401 hCM differentiallydown-regulated genes

Table S9: Gene association network information for hCM enriched genes. This file shows the details of gene-gene interactions among the 695 hCM differentially upregulated genes

Table S10: Gene association network information for hCM enriched genes that were involved in muscle contraction. This file shows the details of gene-gene interactions among the hCM enriched genes that were involved in muscle contraction. Network was shown in Figure S2

Table S11: Gene association network information for hCM enriched genes that were involved in heart development. This file shows the details of gene-gene interactions among the hCM enriched genes that were involved in heart development. Network wasshown in Figure S2

Table S12: Gene association network information for hCM enriched genes that were involved in cardiac transcriptional regulation. This file shows the details of gene-gene interactions among the hCM enriched genes that were involved in cardiac transcriptionalregulation. Network was shown in Figure S2

Table S13: Gene-disease network information for the top hCM up-regulated genes. This file lists all the genes that show association with cardiovascular diseases out of the top 50 up-regulated hCM enriched genes. It also contains the detailed information (nodes and edges) of the gene-disease network that is shown as Figure 1D

**Table S14: Mutations associated with cardiovascular diseases in the top hCM upregulated genes.** This file shows the list of cardiovascular disease-associated mutations found in the top 50 up-regulated hCM enriched genes and the references. The simplifiedtable is shown as Figure 1E

**Table S15: List of genes show 5% or more increase in promoter mCpG level in hCMs compared to hESCs and hNSCs.** This file contains the list of 985 genes that showed at least 5% increase in promoter mCpG level in hCMs compared to hESCs andhNSCs

**Table S16: List of genes show 5% or more decrease in promoter mCpG level in hCMs compared to hESCs and hNSCs.** This file contains the list of 195 genes that showed at least 5% decrease in promoter mCpG level in hCMs compared to hESCs andhNSCs

**Table S17: GO analysis of hCM differentially de-methylated genes-Biological Process.** This file contains the list of GO terms in the category of biological process that were significantly (p<0.05) overrepresented among the 195 hCM differentially demethylatedgenes

**Table S18: GO analysis of hCM differentially de-methylated genes-Molecular Function.** This file contains the list of GO terms in the category of molecular function that were significantly (p<0.05) overrepresented among the 195 hCM differentially demethylated genes

**Table S19: GO analysis of hCM differentially de-methylated genes-Cellular Component.** This file contains the list of GO terms in the category of cellular component that were significantly (p<0.05) overrepresented among the 195 hCM differentially demethylated genes

**Table S20: List of genes show hCM specific promoter de-methylation as well as upregulated expression.** This file contains the list of 29 genes that showed at least 5% decrease in promoter mCpG level and at least a two-fold up-regulation in gene expression in hCMs compared to hESCs and hNSCs

**Table S21: GO analysis of hCM differentially de-methylated as well as up-regulated genes-Biological Process.** This file contains the list of GO terms in the category of biological process that were significantly (p<0.05) overrepresented among the 29 genes listed in TableS20

**Table S22: GO analysis of hCM differentially de-methylated as well as up-regulated genes -Molecular Function.** This file contains the list of GO terms in the category of molecular function that were significantly (p<0.05) overrepresented among the 29 29 genes listed in TableS20

**Table S23: GO analysis of hCM differentially de-methylated as well as up-regulated genes -Cellular Component.** This file contains the list of GO terms in the category of cellular component that were significantly (p<0.05) overrepresented among the 29 29 genes listed in TableS20

Table S24: Primer List

## Abbreviations

cTnT: cardiac troponin T
GO: Gene Ontology
hESCs: human embryonic stem cells
hCM: human cardiomyocyte
hNSCs: hESC-derived neural stem cells
TFs: transcription factors
