# Supplementary material for: Global DNA methylation and transcriptional analyses of human ESC-derived cardiomyocytes
Source: Protein Cell. 2014 Jan 29;5(1):59–68. doi: 10.1007/s13238-013-0016-x (PMC3938846; doi:10.1007/s13238-013-0016-x)
Supplement: Supplementary file 4 — Table S1: List of genes differentially up-regulated in hCMs compared to hESCs and hNSCs. This file contains the list of 695 genes that showed at least a two-fold increaseof transcript expression in hCMs compared to hESCs and hNSCs [file 13238_2013_16_MOESM4_ESM.pdf]

Gene name	Description and ID	Cardio vs ES	Cardio vs NSC
PLN	phospholamban [Source:HGNC Symbol;Acc:9080]	10.51956298	10.53302087
MYL7	myosin, light chain 7, regulatory [Source:HGNC Symbol;Acc:21719]	9.683048044	10.96719567
SMPX	small muscle protein, X-linked [Source:HGNC Symbol;Acc:11122]	9.91336154	9.678290914
MYH6	myosin, heavy chain 6, cardiac muscle, alpha [Source:HGNC Symbol;Acc:7576]	9.518535857	9.794227963
MYOZ2	myozenin 2 [Source:HGNC Symbol;Acc:1330]	9.48535322	9.510284971
MYL2	myosin, light chain 2, regulatory, cardiac, slow [Source:HGNC Symbol;Acc:7583]	9.4230166	9.526811594
SYNPO2L	synaptopodin 2-like [Source:HGNC Symbol;Acc:23532]	9.25673506	9.420535272
CSRP3	cysteine and glycine-rich protein 3 (cardiac LIM protein) [Source:HGNC Symbol;Acc:2472]	9.149525101	9.187714594
TTN	titin [Source:HGNC Symbol;Acc:12403]	9.140593819	9.74528868
UNC45B	unc-45 homolog B (C. elegans) [Source:HGNC Symbol;Acc:14304]	9.020141934	9.030576104
MYL4	myosin, light chain 4, alkali; atrial, embryonic [Source:HGNC Symbol;Acc:7585]	9.22112583	9.002175408
LDB3	LIM domain binding 3 [Source:HGNC Symbol;Acc:15710]	8.929639919	8.979982833
MYH7	myosin, heavy chain 7, cardiac muscle, beta [Source:HGNC Symbol;Acc:7577]	8.704774119	8.720207235
ANKRD1	ankyrin repeat domain 1 (cardiac muscle) [Source:HGNC Symbol;Acc:15819]	9.714009013	8.638574866
NPPB	natriuretic peptide B [Source:HGNC Symbol;Acc:7940]	8.477789119	8.589353528
TNNT2	troponin T type 2 (cardiac) [Source:HGNC Symbol;Acc:11949]	8.452019224	9.944182113
NPNT	nephronectin [Source:HGNC Symbol;Acc:27405]	8.250716674	8.31867258
HSPB3	heat shock 27kDa protein 3 [Source:HGNC Symbol;Acc:5248]	8.452281116	8.141237584
HOPX	HOP homeobox [Source:HGNC Symbol;Acc:24961]	8.250478735	8.087433669
GATA6	GATA binding protein 6 [Source:HGNC Symbol;Acc:4174]	8.012008327	8.048913487
NPPA	natriuretic peptide A [Source:HGNC Symbol;Acc:7939]	7.983519001	8.03271611
TNNC1	troponin C type 1 (slow) [Source:HGNC Symbol;Acc:11943]	7.817135367	9.713527394
ACTN2	actinin, alpha 2 [Source:HGNC Symbol;Acc:164]	7.784577156	9.089164511
SMYD1	SET and MYND domain containing 1 [Source:HGNC Symbol;Acc:20986]	7.648559811	7.804898231
POPDC2	popeye domain containing 2 [Source:HGNC Symbol;Acc:17648]	8.402616293	7.562352603
TRIM55	tripartite motif containing 55 [Source:HGNC Symbol;Acc:14215]	7.373137025	7.267597143
CDKN2B	cyclin-dependent kinase inhibitor 2B (p15, inhibits CDK4) [Source:HGNC Symbol;Acc:1788]	7.551247101	7.236497703
MYL3	myosin, light chain 3, alkali; ventricular, skeletal, slow [Source:HGNC Symbol;Acc:7584]	7.246851697	7.221066118
LRRC10	leucine rich repeat containing 10 [Source:HGNC Symbol;Acc:20264]	7.323030049	7.188206433
SPHKAP	SPHK1 interactor, AKAP domain containing [Source:HGNC Symbol;Acc:30619]	7.330259468	7.168662602
MYLK3	myosin light chain kinase 3 [Source:HGNC Symbol;Acc:29826]	7.250097452	7.07127301
LMOD2	leiomodin 2 (cardiac) [Source:HGNC Symbol;Acc:6648]	7.044116937	7.201199056
GATA4	GATA binding protein 4 [Source:HGNC Symbol;Acc:4173]	7.037629174	7.08958716
TECRL	trans-2,3-enoyl-CoA reductase-like [Source:HGNC Symbol;Acc:27365]	7.193736054	7.019114275
HAPLN1	hyaluronan and proteoglycan link protein 1 [Source:HGNC Symbol;Acc:2380]	6.990331264	7.059102954
XIRP1	xin actin-binding repeat containing 1 [Source:HGNC Symbol;Acc:14301]	6.963610313	7.210248185
AQP1	aquaporin 1 (Colton blood group) [Source:HGNC Symbol;Acc:633]	7.177085607	6.946817667
FILIP1	filamin A interacting protein 1 [Source:HGNC Symbol;Acc:21015]	6.920428571	7.794390913
DUSP27	dual specificity phosphatase 27 (putative) [Source:HGNC Symbol;Acc:25034]	6.857012082	7.013080847
IGFBP7	insulin-like growth factor binding protein 7 [Source:HGNC Symbol;Acc:5476]	8.452781256	6.784095644
SYTL5	synaptotagmin-like 5 [Source:HGNC Symbol;Acc:15589]	6.76385208	6.888909035
POSTN	periostin, osteoblast specific factor [Source:HGNC Symbol;Acc:16953]	8.213897694	6.707659534
TNNI1	troponin I type 1 (skeletal, slow) [Source:HGNC Symbol;Acc:11945]	6.705106323	6.794815577
PLCXD3	phosphatidylinositol-specific phospholipase C, X domain containing 3 [Source:HGNC Symbol;Acc:31822]	7.092591209	6.698815928
MYOCD	myocardin [Source:HGNC Symbol;Acc:16067]	6.69624243	6.908026166
APOBEC2	apolipoprotein B mRNA editing enzyme, catalytic polypeptide-like 2 [Source:HGNC Symbol;Acc:605]	6.833633349	6.664638177
RYR2	ryanodine receptor 2 (cardiac) [Source:HGNC Symbol;Acc:10484]	6.778031068	6.628316321
MASP1	mannan-binding lectin serine peptidase 1 (C4/C2 activating component of Ra-reactive factor) [Source:HGNC Symbol;Acc:6901]	7.240842303	6.569487061
TRIM63	tripartite motif containing 63, E3 ubiquitin protein ligase [Source:HGNC Symbol;Acc:16007]	6.500906584	6.587652769
HAND2	heart and neural crest derivatives expressed 2 [Source:HGNC Symbol;Acc:4808]	6.425795574	6.411606651
CASQ2	calsequestrin 2 (cardiac muscle) [Source:HGNC Symbol;Acc:1513]	7.204662419	6.365908148
HSPB7	heat shock 27kDa protein family, member 7 (cardiovascular) [Source:HGNC Symbol;Acc:5249]	6.447867991	6.295762763
RCSD1	RCSD domain containing 1 [Source:HGNC Symbol;Acc:28310]	6.275273643	6.600905919
TRDN	triadin [Source:HGNC Symbol;Acc:12261]	6.262050507	7.208815318
CORIN	corin, serine peptidase [Source:HGNC Symbol;Acc:19012]	7.653017074	6.231240579
ACE2	angiotensin I converting enzyme (peptidyl-dipeptidase A) 2 [Source:HGNC Symbol;Acc:13557]	6.206629969	6.300730869
SERPINA1	serpin peptidase inhibitor, clade A (alpha-1 antiproteinase, antitrypsin), member 1 [Source:HGNC Symbol;Acc:8941]	6.304037302	6.205224228
MYBPC3	myosin binding protein C, cardiac [Source:HGNC Symbol;Acc:7551]	6.01967711	6.115402912
C7	complement component 7 [Source:HGNC Symbol;Acc:1346]	8.260692108	6.01745698
SYNPO2	synaptopodin 2 [Source:HGNC Symbol;Acc:17732]	5.954817429	6.771396362
CAV2	caveolin 2 [Source:HGNC Symbol;Acc:1528]	5.940893529	6.328288119
CRYAB	crystallin, alpha B [Source:HGNC Symbol;Acc:2389]	5.935066651	6.305402948
MYOM1	myomesin 1, 185kDa [Source:HGNC Symbol;Acc:7613]	5.928375111	5.942358498
ASB2	ankyrin repeat and SOCS box containing 2 [Source:HGNC Symbol;Acc:16012]	5.906016606	6.011695356
GJA3	gap junction protein, alpha 3, 46kDa [Source:HGNC Symbol;Acc:4277]	5.899047054	7.122944061
GHR	growth hormone receptor [Source:HGNC Symbol;Acc:4263]	6.524302471	5.888957933
BBS5	Bardet-Biedl syndrome 5 [Source:HGNC Symbol;Acc:970]	7.085189078	5.86104633
NEXN	nexilin (F actin binding protein) [Source:HGNC Symbol;Acc:29557]	6.017296052	5.851277932
MFAP5	microfibrillar associated protein 5 [Source:HGNC Symbol;Acc:29673]	5.833873116	6.09428939
MGP	matrix Gla protein [Source:HGNC Symbol;Acc:7060]	5.808147477	5.784055364
RSPO3	R-spondin 3 [Source:HGNC Symbol;Acc:20866]	5.838938992	5.764014019
TNNI3K	FPGT-TNNI3K fusion protein isoform b [Source:RefSeq peptide;Acc:NP_001186256]	5.743743518	5.94151794
CKMT2	creatine kinase, mitochondrial 2 (sarcomeric) [Source:HGNC Symbol;Acc:1996]	5.731750639	6.977746462

MICAL2	microtubule associated monoxygenase, calponin and LIM domain containing 2 [Source:HGNC Symbol;Acc:24693]	5.724084653	6.282215087
PDLIM5	PDZ and LIM domain 5 [Source:HGNC Symbol;Acc:17468]	6.28506473	5.702176279
SULT1E1	sulfotransferase family 1E, estrogen-preferring, member 1 [Source:HGNC Symbol;Acc:11377]	5.695016886	5.730610594
CLIC5	chloride intracellular channel 5 [Source:HGNC Symbol;Acc:13517]	5.710061366	5.683038375
PDE4DIP	TNNI3 interacting kinase [Source:HGNC Symbol;Acc:19661]	5.651810887	5.763026873
C1orf105	chromosome 1 open reading frame 105 [Source:HGNC Symbol;Acc:29591]	5.880343194	5.558800703
MMP1	matrix metallopeptidase 1 (interstitial collagenase) [Source:HGNC Symbol;Acc:7155]	5.783961585	5.542137796
RELN	reelin [Source:HGNC Symbol;Acc:9957]	7.053337661	5.536651466
LEFTY2	left-right determination factor 2 [Source:HGNC Symbol;Acc:3122]	5.533921939	6.504193495
C5orf46	chromosome 5 open reading frame 46 [Source:HGNC Symbol;Acc:33768]	5.54128843	5.528948581
ENO3	enolase 3 (beta, muscle) [Source:HGNC Symbol;Acc:3354]	5.483619264	6.911731563
ACTA2	actin, alpha 2, smooth muscle, aorta [Source:HGNC Symbol;Acc:130]	5.425159564	5.586194259
CBLN2	cerebellin 2 precursor [Source:HGNC Symbol;Acc:1544]	5.558760577	5.411758764
GSG1	germ cell associated 1 [Source:HGNC Symbol;Acc:19716]	5.38154256	5.541766307
CYP2J2	cytochrome P450, family 2, subfamily J, polypeptide 2 [Source:HGNC Symbol;Acc:2634]	5.3644271	6.373027382
JPH2	junctophilin 2 [Source:HGNC Symbol;Acc:14202]	5.503950869	5.356740993
MYO18B	myosin XVIIIB [Source:HGNC Symbol;Acc:18150]	5.35314239	5.786420245
C21orf7	chromosome 21 open reading frame 7 [Source:HGNC Symbol;Acc:16457]	5.341191568	5.389991449
ABRA	actin-binding Rho activating protein [Source:HGNC Symbol;Acc:30655]	5.464788018	5.340692628
SLC8A1	solute carrier family 8 (sodium/calcium exchanger), member 1 [Source:HGNC Symbol;Acc:11068]	6.697277985	5.311511454
IGFBP3	insulin-like growth factor binding protein 3 [Source:HGNC Symbol;Acc:5472]	5.281587923	5.343524727
NKX2-5	NK2 homeobox 5 [Source:HGNC Symbol;Acc:2488]	5.276619134	5.318893908
EDN1	endothelin 1 [Source:HGNC Symbol;Acc:3176]	5.23121772	5.618159105
LOC653513	phosphodiesterase 4D interacting protein pseudogene	5.212381125	5.198816559
C15orf52	chromosome 15 open reading frame 52 [Source:HGNC Symbol;Acc:33488]	5.192978911	5.918630472
EYA4	eyes absent homolog 4 (Drosophila) [Source:HGNC Symbol;Acc:3522]	5.832521575	5.160979898
DGKI	diacylglycerol kinase, iota [Source:HGNC Symbol;Acc:2855]	5.776656915	5.151648358
TBX5	T-box 5 [Source:HGNC Symbol;Acc:11604]	5.107802724	5.14740947
ITGB1BP2	integrin beta 1 binding protein (melusin) 2 [Source:HGNC Symbol;Acc:6154]	6.275810197	5.097896102
SLC5A1	solute carrier family 5 (sodium/glucose cotransporter), member 1 [Source:HGNC Symbol;Acc:11036]	5.219318659	5.079557014
GPRC5A	G protein-coupled receptor, family C, group 5, member A [Source:HGNC Symbol;Acc:9836]	5.072541896	5.181596195
INHBA	inhibin, beta A [Source:HGNC Symbol;Acc:6066]	5.147059611	5.045977615
PLCL1	phospholipase C-like 1 [Source:HGNC Symbol;Acc:9063]	5.952187796	5.041382625
BVES	blood vessel epicardial substance [Source:HGNC Symbol;Acc:1152]	5.552883117	5.036801337
VCAM1	vascular cell adhesion molecule 1 [Source:HGNC Symbol;Acc:12663]	7.651247496	4.999369044
TGM2	transglutaminase 2 (C polypeptide, protein-glutamine-gamma-glutamyltransferase) [Source:HGNC Symbol;Acc:11778]	7.629746794	4.976532929
RGS5	regulator of G-protein signaling 5 [Source:HGNC Symbol;Acc:10001]	4.965997416	5.729400235
GUCY1A3	guanylate cyclase 1, soluble, alpha 3 [Source:HGNC Symbol;Acc:4685]	7.990587542	4.961909975
FRMD3	FERM domain containing 3 [Source:HGNC Symbol;Acc:24125]	6.174420569	4.960405566
ADAMTS9	ADAM metallopeptidase with thrombospondin type 1 motif, 9 [Source:HGNC Symbol;Acc:13202]	5.788228423	4.955851478
FLRT2	fibronectin leucine rich transmembrane protein 2 [Source:HGNC Symbol;Acc:3761]	4.94852864	5.380600175
TM4SF1	transmembrane 4 L six family member 1 [Source:HGNC Symbol;Acc:11853]	4.93883911	4.977312044
FABP3	fatty acid binding protein 3, muscle and heart (mammary-derived growth inhibitor) [Source:HGNC Symbol;Acc:3557]	4.915996895	6.836111197
CASQ1	calsequestrin 1 (fast-twitch, skeletal muscle) [Source:HGNC Symbol;Acc:1512]	4.915785061	5.22922426
MYH7B	myosin, heavy chain 7B, cardiac muscle, beta [Source:HGNC Symbol;Acc:15906]	4.901631607	4.96658216
TMOD1	tropomodulin 1 [Source:HGNC Symbol;Acc:11871]	4.886436396	5.764246459
CORO6	coronin 6 [Source:HGNC Symbol;Acc:21356]	4.883743475	5.178794029
CACNA1C	calcium channel, voltage-dependent, L type, alpha 1C subunit [Source:HGNC Symbol;Acc:1390]	4.970909798	4.869675683
SERPINE1	serpin peptidase inhibitor, clade E (nexin, plasminogen activator inhibitor type 1), member 1 [Source:HGNC Symbol;Acc:8583]	4.860576618	6.369659758
MITF	microphthalmia-associated transcription factor [Source:HGNC Symbol;Acc:7105]	5.806916971	4.842064668
RBM20	RNA binding motif protein 20 [Source:HGNC Symbol;Acc:27424]	5.48481006	4.839849027
LOX	lysyl oxidase [Source:HGNC Symbol;Acc:6664]	4.937654527	4.777910635
CCDC80	coiled-coil domain containing 80 [Source:HGNC Symbol;Acc:30649]	6.336886176	4.767390512
RBM24	RNA binding motif protein 24 [Source:HGNC Symbol;Acc:21539]	4.7959802	4.734891104
BAMBI	BMP and activin membrane-bound inhibitor homolog (Xenopus laevis) [Source:HGNC Symbol;Acc:30251]	4.689634687	5.299232759
LRRC39	leucine rich repeat containing 39 [Source:HGNC Symbol;Acc:28228]	5.429890209	4.685294797
CMYA5	cardiomyopathy associated 5 [Source:HGNC Symbol;Acc:14305]	4.682412295	4.726400792
HBEGF	heparin-binding EGF-like growth factor [Source:HGNC Symbol;Acc:3059]	5.018569174	4.666490133
PGM5	phosphoglucomutase 5 [Source:HGNC Symbol;Acc:8908]	5.754383615	4.641832281
THBD	thrombomodulin [Source:HGNC Symbol;Acc:11784]	4.617506311	4.643280426
NCAM1	neural cell adhesion molecule 1 [Source:HGNC Symbol;Acc:7656]	4.599113395	4.670038247
GCOM1	GRINL1A complex locus 1 [Source:HGNC Symbol;Acc:26424]	4.539596096	5.651449572
PITX2	paired-like homeodomain 2 [Source:HGNC Symbol;Acc:9005]	4.524937516	4.997411585
PCDH7	protocadherin 7 [Source:HGNC Symbol;Acc:8659]	4.829372566	4.508221908
CNN1	calponin 1, basic, smooth muscle [Source:HGNC Symbol;Acc:2155]	4.498172036	5.205638705
LOXL4	lysyl oxidase-like 4 [Source:HGNC Symbol;Acc:17171]	4.529037597	4.49755327
KLHL31	kelch-like 31 (Drosophila) [Source:HGNC Symbol;Acc:21353]	4.493443031	4.951624707
TIMP3	TIMP metallopeptidase inhibitor 3 [Source:HGNC Symbol;Acc:11822]	8.046071607	4.476509638
FGF1	fibroblast growth factor 1 (acidic) [Source:HGNC Symbol;Acc:3665]	4.475200987	4.817952431
COL21A1	collagen, type XXI, alpha 1 [Source:HGNC Symbol;Acc:17025]	4.47384744	4.778008173
MB	myoglobin [Source:HGNC Symbol;Acc:6915]	4.460138462	5.256318105
TPM2	tropomyosin 2 (beta) [Source:HGNC Symbol;Acc:12011]	6.049999407	4.452519754

PPP1R12B	protein phosphatase 1, regulatory subunit 12B [Source:HGNC Symbol;Acc:7619]	5.017316019	4.440918736
CALB2	calbindin 2 [Source:HGNC Symbol;Acc:1435]	4.438248408	4.496617923
GREM1	gremlin 1 [Source:HGNC Symbol;Acc:2001]	4.435826932	4.531978656
CCDC85A	coiled-coil domain containing 85A [Source:HGNC Symbol;Acc:29400]	4.628968023	4.401310225
DACT1	dapper, antagonist of beta-catenin, homolog 1 (Xenopus laevis) [Source:HGNC Symbol;Acc:17748]	4.371019387	4.925309227
FLNC	filamin C, gamma [Source:HGNC Symbol;Acc:3756]	5.199968343	4.370411535
POPDC3	popeye domain containing 3 [Source:HGNC Symbol;Acc:17649]	4.288096896	5.33795594
GADD45B	growth arrest and DNA-damage-inducible, beta [Source:HGNC Symbol;Acc:4096]	4.283618744	4.310003408
CKM	creatine kinase, muscle [Source:HGNC Symbol;Acc:1994]	4.274123967	4.410938629
PALM2	paralemmin 2 [Source:HGNC Symbol;Acc:15845]	4.401727676	4.262473608
RASGRP3	RAS guanyl releasing protein 3 (calcium and DAG-regulated) [Source:HGNC Symbol;Acc:14545]	4.364960221	4.250705154
TCEA3	transcription elongation factor A (SII), 3 [Source:HGNC Symbol;Acc:11615]	4.564334974	4.249863147
GLS	glutaminase [Source:HGNC Symbol;Acc:4331]	4.839427882	4.240646894
PPARGC1A	peroxisome proliferator-activated receptor gamma, coactivator 1 alpha [Source:HGNC Symbol;Acc:9237]	4.564589293	4.228540981
PGAM2	phosphoglycerate mutase 2 (muscle) [Source:HGNC Symbol;Acc:8889]	4.225140363	6.089744173
BNC1	basonuclin 1 [Source:HGNC Symbol;Acc:1081]	4.46489	4.209971333
LMOD1	leiomodin 1 (smooth muscle) [Source:HGNC Symbol;Acc:6647]	4.191709955	4.443053112
PPP1R3C	protein phosphatase 1, regulatory subunit 3C [Source:HGNC Symbol;Acc:9293]	4.184343092	5.377028835
HRC	histidine rich calcium binding protein [Source:HGNC Symbol;Acc:5178]	4.169851437	4.623754598
RNF207	ring finger protein 207 [Source:HGNC Symbol;Acc:32947]	4.167994068	5.029016329
FAM78A	family with sequence similarity 78, member A [Source:HGNC Symbol;Acc:25465]	4.167262714	4.475770626
NEBL	nebulette [Source:HGNC Symbol;Acc:16932]	4.140315839	5.218207647
COL12A1	collagen, type XII, alpha 1 [Source:HGNC Symbol;Acc:2188]	4.24254316	4.131641057
ANXA8	annexin A8 [Source:HGNC Symbol;Acc:546]	4.108254115	4.269978227
AARSD1	alanyl-tRNA synthetase domain containing 1 [Source:HGNC Symbol;Acc:28417]	4.09629024	4.49820923
KIF26B	kinesin family member 26B [Source:HGNC Symbol;Acc:25484]	5.835828762	4.093566301
TMEM40	transmembrane protein 40 [Source:HGNC Symbol;Acc:25620]	4.120951024	4.088514275
FGF5	fibroblast growth factor 5 [Source:HGNC Symbol;Acc:3683]	4.111512767	4.084648676
SORBS2	sorbin and SH3 domain containing 2 [Source:HGNC Symbol;Acc:24098]	6.346527601	4.077064014
C18orf1	chromosome 18 open reading frame 1 [Source:HGNC Symbol;Acc:1224]	4.185296877	4.071498157
AFP	alpha-fetoprotein [Source:HGNC Symbol;Acc:317]	4.056965484	4.653591857
HSPB8	heat shock 22kDa protein 8 [Source:HGNC Symbol;Acc:30171]	4.029594886	5.333807436
GDF6	growth differentiation factor 6 [Source:HGNC Symbol;Acc:4221]	7.375474613	4.02922441
SNTA1	syntrophin, alpha 1 [Source:HGNC Symbol;Acc:11167]	4.250711284	4.022870927
ALDH1A2	aldehyde dehydrogenase 1 family, member A2 [Source:HGNC Symbol;Acc:15472]	4.086647853	4.018751579
CACNB2	calcium channel, voltage-dependent, beta 2 subunit [Source:HGNC Symbol;Acc:1402]	4.075539896	4.018009687
AGPAT9	1-acylglycerol-3-phosphate O-acyltransferase 9 [Source:HGNC Symbol;Acc:28157]	4.007638668	4.836101398
C4orf32	chromosome 4 open reading frame 32 [Source:HGNC Symbol;Acc:26813]	4.006175244	4.608902493
PCDH20	protocadherin 20 [Source:HGNC Symbol;Acc:14257]	3.993009607	4.96200615
TAGLN	transgelin [Source:HGNC Symbol;Acc:11553]	3.988337073	3.999212863
THBS1	thrombospondin 1 [Source:HGNC Symbol;Acc:11785]	4.47081521	3.984513539
COL8A1	collagen, type VIII, alpha 1 [Source:HGNC Symbol;Acc:2215]	4.463234596	3.98345625
AQP10	aquaporin 10 [Source:HGNC Symbol;Acc:16029]	4.036107495	3.966214459
PTH1R	parathyroid hormone 1 receptor [Source:HGNC Symbol;Acc:9608]	3.952966248	3.995915063
AMIGO2	adhesion molecule with Ig-like domain 2 [Source:HGNC Symbol;Acc:24073]	3.940535998	5.093044961
EMILIN2	elastin microfibril interfacer 2 [Source:HGNC Symbol;Acc:19881]	3.937766139	5.227801696
YPEL2	yippee-like 2 (Drosophila) [Source:HGNC Symbol;Acc:18326]	3.934908722	4.743664542
DMD	dystrophin [Source:HGNC Symbol;Acc:2928]	3.934591414	4.236926196
CCDC141	coiled-coil domain containing 141 [Source:HGNC Symbol;Acc:26821]	3.921679661	4.405129209
LMO7	LIM domain 7 [Source:HGNC Symbol;Acc:6646]	3.912279708	5.979542544
ACTA1	actin, alpha 1, skeletal muscle [Source:HGNC Symbol;Acc:129]	3.909288772	5.927630542
TBX3	T-box 3 [Source:HGNC Symbol;Acc:11602]	4.002176689	3.89827799
ITGB6	integrin, beta 6 [Source:HGNC Symbol;Acc:6161]	3.996495104	3.87832235
SAMD4A	sterile alpha motif domain containing 4A [Source:HGNC Symbol;Acc:23023]	4.482013086	3.853192857
LIMS2	LIM and senescent cell antigen-like domains 2 [Source:HGNC Symbol;Acc:16084]	3.837574887	4.0861631
XPO4	exportin 4 [Source:HGNC Symbol;Acc:17796]	3.828685064	4.041548514
GPRIN3	GPRIN family member 3 [Source:HGNC Symbol;Acc:27733]	3.815976417	3.948232509
FBXO40	F-box protein 40 [Source:HGNC Symbol;Acc:29816]	3.782329162	3.841905068
CMAH	cytidine monophospho-N-acetylneuraminic acid hydroxylase, pseudogene [Source:HGNC Symbol;Acc:2098]	3.813634983	3.777427829
PRRX1	paired related homeobox 1 [Source:HGNC Symbol;Acc:9142]	5.28616409	3.777210634
SH3BGR	SH3 domain binding glutamic acid-rich protein [Source:HGNC Symbol;Acc:10822]	3.775274305	5.414330613
ODAM	odontogenic, ameloblast asssociated [Source:HGNC Symbol;Acc:26043]	3.770150841	4.000983698
ZFPM2	zinc finger protein, multitype 2 [Source:HGNC Symbol;Acc:16700]	6.790240204	3.757175873
GAS2	growth arrest-specific 2 [Source:HGNC Symbol;Acc:4167]	4.014995108	3.749369129
ACTC1	actin, alpha, cardiac muscle 1 [Source:HGNC Symbol;Acc:143]	3.734713366	6.762926976
TXLNB	taxilin beta [Source:HGNC Symbol;Acc:21617]	4.567009829	3.731192855
EXTL1	exostoses (multiple)-like 1 [Source:HGNC Symbol;Acc:3515]	3.84245012	3.723439576
GPR183	G protein-coupled receptor 183 [Source:HGNC Symbol;Acc:3128]	5.493169636	3.702574374
SULF1	sulfatase 1 [Source:HGNC Symbol;Acc:20391]	5.09674084	3.696635414
MYL9	myosin, light chain 9, regulatory [Source:HGNC Symbol;Acc:15754]	3.686553302	4.299079977
GPR124	G protein-coupled receptor 124 [Source:HGNC Symbol;Acc:17849]	4.503723834	3.68301383
COL1A1	collagen, type I, alpha 1 [Source:HGNC Symbol;Acc:2197]	3.676693168	4.108510574
DKK3	dickkopf 3 homolog (Xenopus laevis) [Source:HGNC Symbol;Acc:2893]	3.675077508	3.70103591
PRSS35	protease, serine, 35 [Source:HGNC Symbol;Acc:21387]	6.38029479	3.659907006
LGI2	leucine-rich repeat LGI family, member 2 [Source:HGNC Symbol;Acc:18710]	4.638385578	3.658732569

FN1	fibronectin 1 [Source:HGNC Symbol;Acc:3778]	5.436184474	3.653527953
ALPK2	alpha-kinase 2 [Source:HGNC Symbol;Acc:20565]	8.069528368	3.647151766
PRDM6	PR domain containing 6 [Source:HGNC Symbol;Acc:9350]	3.851703099	3.639483105
TPM1	tropomyosin 1 (alpha) [Source:HGNC Symbol;Acc:12010]	3.637236226	6.143185497
APOA1	apolipoprotein A-I [Source:HGNC Symbol;Acc:600]	3.96717492	3.629650588
MMP15	matrix metallopeptidase 15 (membrane-inserted) [Source:HGNC Symbol;Acc:7161]	3.628150535	3.68927269
LBH	limb bud and heart development homolog (mouse) [Source:HGNC Symbol;Acc:29532]	4.113576624	3.606374943
SYNM	synemin, intermediate filament protein [Source:HGNC Symbol;Acc:24466]	4.204370994	3.596166759
CDC42EP3	CDC42 effector protein (Rho GTPase binding) 3 [Source:HGNC Symbol;Acc:16943]	3.788450748	3.589516376
C13orf31	laccase (multicopper oxidoreductase) domain containing 1 [Source:HGNC Symbol;Acc:26789]	3.564474713	4.855031462
PPP1R3A	protein phosphatase 1, regulatory subunit 3A [Source:HGNC Symbol;Acc:9291]	3.563366821	3.636446622
NIPSNAP3B	nipsnap homolog 3B (C. elegans) [Source:HGNC Symbol;Acc:23641]	4.044010104	3.56154586
EDNRA	endothelin receptor type A [Source:HGNC Symbol;Acc:3179]	4.530980289	3.561002066
FBXO32	F-box protein 32 [Source:HGNC Symbol;Acc:16731]	4.289660926	3.551195378
TBX18	T-box 18 [Source:HGNC Symbol;Acc:11595]	4.44934012	3.541477809
PTPLA	protein tyrosine phosphatase-like (proline instead of catalytic arginine), member A [Source:HGNC Symbol;Acc:9639]	3.523972607	4.440632464
WT1	Wilms tumor 1 [Source:HGNC Symbol;Acc:12796]	3.522015935	3.576377026
PKP2	plakophilin 2 [Source:HGNC Symbol;Acc:9024]	3.518005782	5.148726071
ZAK	sterile alpha motif and leucine zipper containing kinase AZK	3.84646871	3.51298724
C13orf15	regulator of cell cycle [Source:HGNC Symbol;Acc:20369]	4.646950345	3.508402146
HIST2H2BE	histone cluster 2, H2be [Source:HGNC Symbol;Acc:4760]	4.852112465	3.500020323
SYPL2	synaptophysin-like 2 [Source:HGNC Symbol;Acc:27638]	3.495341917	3.73749005
EPSTI1	epithelial stromal interaction 1 (breast) [Source:HGNC Symbol;Acc:16465]	3.492899429	3.953554894
CXCL14	chemokine (C-X-C motif) ligand 14 [Source:HGNC Symbol;Acc:10640]	3.488075927	4.749968412
STX11	syntaxin 11 [Source:HGNC Symbol;Acc:11429]	3.711269513	3.477239434
ADPRHL1	ADP-ribosylhydrolase like 1 [Source:HGNC Symbol;Acc:21303]	3.861605168	3.467278111
EFEMP2	EGF containing fibulin-like extracellular matrix protein 2 [Source:HGNC Symbol;Acc:3219]	3.464156991	3.95874873
ADAM19	ADAM metallopeptidase domain 19 [Source:HGNC Symbol;Acc:197]	3.458750579	4.221630995
DSC3	desmocollin 3 [Source:HGNC Symbol;Acc:3037]	3.453827091	4.537216483
KANK4	KN motif and ankyrin repeat domains 4 [Source:HGNC Symbol;Acc:27263]	3.511430214	3.444812872
GATA5	GATA binding protein 5 [Source:HGNC Symbol;Acc:15802]	3.96816957	3.435379919
CASZ1	castor zinc finger 1 [Source:HGNC Symbol;Acc:26002]	3.429145917	3.43108908
PLEKHA7	pleckstrin homology domain containing, family A member 7 [Source:HGNC Symbol;Acc:27049]	3.591801679	3.418910691
FBN2	fibrillin 2 [Source:HGNC Symbol;Acc:3604]	4.08852035	3.418469983
RFTN1	raftlin, lipid raft linker 1 [Source:HGNC Symbol;Acc:30278]	4.65327228	3.409564904
CDKN1A	cyclin-dependent kinase inhibitor 1A (p21, Cip1) [Source:HGNC Symbol;Acc:1784]	4.214325703	3.398875097
GPR126	G protein-coupled receptor 126 [Source:HGNC Symbol;Acc:13841]	4.467689668	3.398686907
TMEM56	transmembrane protein 56 [Source:HGNC Symbol;Acc:26477]	3.383261865	3.466118039
G0S2	G0/G1switch 2 [Source:HGNC Symbol;Acc:30229]	3.369870248	3.892070786
KCNA5	potassium voltage-gated channel, shaker-related subfamily, member 5 [Source:HGNC Symbol;Acc:6224]	3.363810594	3.742087652
PDLIM3	PDZ and LIM domain 3 [Source:HGNC Symbol;Acc:20767]	4.193211975	3.361789589
FAM134B	family with sequence similarity 134, member B [Source:HGNC Symbol;Acc:25964]	3.874440157	3.344425727
BMP5	bone morphogenetic protein 5 [Source:HGNC Symbol;Acc:1072]	5.521284392	3.341466403
GMPR	guanosine monophosphate reductase [Source:HGNC Symbol;Acc:4376]	3.333102976	4.171587453
PTRF	polymerase I and transcript release factor [Source:HGNC Symbol;Acc:9688]	3.33288344	4.701753772
MPPED2	metallophosphoesterase domain containing 2 [Source:HGNC Symbol;Acc:1180]	3.332234965	5.302924734
C17orf91	MIR22 host gene (non-protein coding) [Source:HGNC Symbol;Acc:28219]	4.804595663	3.319238447
TGFBI	transforming growth factor, beta-induced, 68kDa [Source:HGNC Symbol;Acc:11771]	3.315934365	5.927413583
VEGFC	vascular endothelial growth factor C [Source:HGNC Symbol;Acc:12682]	3.875628672	3.311070597
CSRP1	cysteine and glycine-rich protein 1 [Source:HGNC Symbol;Acc:2469]	4.117052485	3.287666063
INPP4B	inositol polyphosphate-4-phosphatase, type II, 105kDa [Source:HGNC Symbol;Acc:6075]	3.282013625	3.42244494
COL5A1	collagen, type V, alpha 1 [Source:HGNC Symbol;Acc:2209]	4.222149459	3.279249727
C11orf9	chromosome 11 open reading frame 9 [Source:HGNC Symbol;Acc:1181]	3.800948335	3.275385438
PEAR1	platelet endothelial aggregation receptor 1 [Source:HGNC Symbol;Acc:33631]	3.386738772	3.272098942
CAV1	caveolin 1, caveolae protein, 22kDa [Source:HGNC Symbol;Acc:1527]	3.269144181	3.858154017
SYNPO	synaptopodin [Source:HGNC Symbol;Acc:30672]	4.707407304	3.267633482
DTNA	dystrobrevin, alpha [Source:HGNC Symbol;Acc:3057]	3.266816176	3.704597497
C6orf114	glucose-fructose oxidoreductase domain containing 1 [Source:HGNC Symbol;Acc:21096]	3.301978594	3.264685195
TMEM173	transmembrane protein 173 [Source:HGNC Symbol;Acc:27962]	3.532873504	3.262586904
SLCO3A1	solute carrier organic anion transporter family, member 3A1 [Source:HGNC Symbol;Acc:10952]	3.27079601	3.260297636
FSTL3	follistatin-like 3 (secreted glycoprotein) [Source:HGNC Symbol;Acc:3973]	3.260175943	3.395860142
EPAS1	endothelial PAS domain protein 1 [Source:HGNC Symbol;Acc:3374]	3.252138559	3.926120259
MUM1L1	melanoma associated antigen (mutated) 1-like 1 [Source:HGNC Symbol;Acc:26583]	3.711043974	3.249818718
MATN2	matrilin 2 [Source:HGNC Symbol;Acc:6908]	3.241129232	5.815188161
CHSY3	chondroitin sulfate synthase 3 [Source:HGNC Symbol;Acc:24293]	3.368216931	3.231545177
PNKD	paroxysmal nonkinesigenic dyskinesia [Source:HGNC Symbol;Acc:9153]	3.226127486	3.66970193
ADAMTS5	ADAM metallopeptidase with thrombospondin type 1 motif, 5 [Source:HGNC Symbol;Acc:221]	3.223557003	3.347141329
CTGF	connective tissue growth factor [Source:HGNC Symbol;Acc:2500]	3.524139003	3.220348495
AGT	angiotensinogen (serpin peptidase inhibitor, clade A, member 8) [Source:HGNC Symbol;Acc:333]	5.540128866	3.218657637
AHNAK2	AHNAK nucleoprotein 2 [Source:HGNC Symbol;Acc:20125]	3.40214299	3.218444575
STAT4	signal transducer and activator of transcription 4 [Source:HGNC Symbol;Acc:11365]	3.461616225	3.213801878
NEK7	NIMA (never in mitosis gene a)-related kinase 7 [Source:HGNC Symbol;Acc:13386]	3.426497271	3.208340875
DLC1	deleted in liver cancer 1 [Source:HGNC Symbol;Acc:2897]	4.976104762	3.206743295
EHD3	EH-domain containing 3 [Source:HGNC Symbol;Acc:3244]	3.206292172	3.535477133
ARSJ	arylsulfatase family, member J [Source:HGNC Symbol;Acc:26286]	3.189485951	4.395517834

TSPAN32	tetraspanin 32 [Source:HGNC Symbol;Acc:13410]	3.269662147	3.184953553
ATXN1	ataxin 1 [Source:HGNC Symbol;Acc:10548]	4.971619352	3.184495447
TBX2	T-box 2 [Source:HGNC Symbol;Acc:11597]	3.237111289	3.182624548
PRICKLE1	prickle homolog 1 (Drosophila) [Source:HGNC Symbol;Acc:17019]	3.168728773	5.003646824
ITGAV	integrin, alpha V [Source:HGNC Symbol;Acc:6150]	3.14908452	3.559856802
TM4SF4	transmembrane 4 L six family member 4 [Source:HGNC Symbol;Acc:11856]	3.305096062	3.148017993
GFOD1	glucose-fructose oxidoreductase domain containing 1 [Source:HGNC Symbol;Acc:21096]	3.146192522	3.32191812
SLC4A4	solute carrier family 4, sodium bicarbonate cotransporter, member 4 [Source:HGNC Symbol;Acc:11030]	4.415763617	3.142500731
SLMAP	sarcolemma associated protein [Source:HGNC Symbol;Acc:16643]	3.140633747	3.658860387
DCN	decorin [Source:HGNC Symbol;Acc:2705]	7.792488573	3.12303916
ASPH	aspartate beta-hydroxylase [Source:HGNC Symbol;Acc:757]	4.281290866	3.122582294
PPIC	peptidylprolyl isomerase C (cyclophilin C) [Source:HGNC Symbol;Acc:9256]	3.120383243	4.30692316
DOK4	docking protein 4 [Source:HGNC Symbol;Acc:19868]	3.120212547	3.802146927
GPR133	G protein-coupled receptor 133 [Source:HGNC Symbol;Acc:19893]	3.915849876	3.118377322
SFXN3	sideroflexin 3 [Source:HGNC Symbol;Acc:16087]	3.18152232	3.114776229
SH3RF2	SH3 domain containing ring finger 2 [Source:HGNC Symbol;Acc:26299]	3.266958144	3.114454163
SH3BP5	SH3-domain binding protein 5 (BTK-associated) [Source:HGNC Symbol;Acc:10827]	3.112385136	3.674882864
MGST2	microsomal glutathione S-transferase 2 [Source:HGNC Symbol;Acc:7063]	3.110676994	3.479659201
C9orf150	leucine rich adaptor protein 1-like [Source:HGNC Symbol;Acc:31452]	3.739417542	3.109582336
PDGFD	platelet derived growth factor D [Source:HGNC Symbol;Acc:30620]	3.9466157	3.109295143
F2RL2	coagulation factor II (thrombin) receptor-like 2 [Source:HGNC Symbol;Acc:3539]	3.218976947	3.106845011
DKK1	dickkopf 1 homolog (Xenopus laevis) [Source:HGNC Symbol;Acc:2891]	3.364351143	3.100771984
CAPN3	calpain 3, (p94) [Source:HGNC Symbol;Acc:1480]	3.291303321	3.099764564
MSRB3	methionine sulfoxide reductase B3 [Source:HGNC Symbol;Acc:27375]	5.938221702	3.081893572
KCNE4	potassium voltage-gated channel, Isk-related family, member 4 [Source:HGNC Symbol;Acc:6244]	3.084766872	3.080877408
RRAD	Ras-related associated with diabetes [Source:HGNC Symbol;Acc:10446]	3.342462229	3.080564297
SGMS2	sphingomyelin synthase 2 [Source:HGNC Symbol;Acc:28395]	4.094407737	3.072752539
TCAP	titin-cap (telethonin) [Source:HGNC Symbol;Acc:11610]	3.243732704	3.071411858
KRT17	keratin 17 [Source:HGNC Symbol;Acc:6427]	3.071094631	3.289504029
DKK2	dickkopf 2 homolog (Xenopus laevis) [Source:HGNC Symbol;Acc:2892]	3.882349307	3.070130151
GP1BB	glycoprotein Ib (platelet), beta polypeptide [Source:HGNC Symbol;Acc:4440]	3.40336298	3.05971128
GPR155	G protein-coupled receptor 155 [Source:HGNC Symbol;Acc:22951]	4.091892409	3.057004385
ANTXR1	anthrax toxin receptor 1 [Source:HGNC Symbol;Acc:21014]	3.055543498	3.218084486
SMAD6	SMAD family member 6 [Source:HGNC Symbol;Acc:6772]	3.727929743	3.052503683
SH3PXD2A	SH3 and PX domains 2A [Source:HGNC Symbol;Acc:23664]	3.084525918	3.039930816
CXCL12	chemokine (C-X-C motif) ligand 12 [Source:HGNC Symbol;Acc:10672]	3.023770547	5.76792036
RGS3	regulator of G-protein signaling 3 [Source:HGNC Symbol;Acc:9999]	3.083525112	3.021870631
MIR21	microRNA 21 [Source:HGNC Symbol;Acc:31586]	4.435860068	3.013103472
SVIL	supervillin [Source:HGNC Symbol;Acc:11480]	4.333390869	3.011515526
SIRPA	signal-regulatory protein alpha [Source:HGNC Symbol;Acc:9662]	3.37222921	3.010042655
MAN1C1	mannosidase, alpha, class 1C, member 1 [Source:HGNC Symbol;Acc:19080]	3.324044593	2.996336796
SMAD7	SMAD family member 7 [Source:HGNC Symbol;Acc:6773]	3.50744217	2.993059731
MRAS	muscle RAS oncogene homolog [Source:HGNC Symbol;Acc:7227]	2.983110395	3.388090149
CFI	complement factor I [Source:HGNC Symbol;Acc:5394]	4.085440187	2.982462024
ME1	malic enzyme 1, NADP(+)-dependent, cytosolic [Source:HGNC Symbol;Acc:6983]	2.9812966	3.910515021
C6orf142	muscular LMNA-interacting protein [Source:HGNC Symbol;Acc:21355]	3.737105435	2.979728594
GRIA3	glutamate receptor, ionotropic, AMPA 3 [Source:HGNC Symbol;Acc:4573]	3.464327022	2.978299924
SLC40A1	solute carrier family 40 (iron-regulated transporter), member 1 [Source:HGNC Symbol;Acc:10909]	5.182081766	2.976341607
IGF2	insulin-like growth factor 2 (somatomedin A) [Source:HGNC Symbol;Acc:5466]	6.350185867	2.975622131
ANO4	anoctamin 4 [Source:HGNC Symbol;Acc:23837]	2.965610789	3.676386918
CAMK2D	calcium/calmodulin-dependent protein kinase II delta [Source:HGNC Symbol;Acc:1462]	4.041471205	2.95892519
GC	group-specific component (vitamin D binding protein) [Source:HGNC Symbol;Acc:4187]	2.945318129	3.2508194
B3GALT2	UDP-Gal:betaGlcNAc beta 1,3-galactosyltransferase, polypeptide 2 [Source:HGNC Symbol;Acc:917]	5.486363215	2.937385398
AHNAK	AHNAK nucleoprotein [Source:HGNC Symbol;Acc:347]	2.937322613	3.28981027
IER3	immediate early response 3 [Source:HGNC Symbol;Acc:5392]	2.933350649	4.68671059
KRT8	keratin 8 [Source:HGNC Symbol;Acc:6446]	2.930783835	5.057879375
CLSTN2	calsyntenin 2 [Source:HGNC Symbol;Acc:17448]	3.522039267	2.929115518
LTBP1	latent transforming growth factor beta binding protein 1 [Source:HGNC Symbol;Acc:6714]	3.097008108	2.919967211
LRCH1	leucine-rich repeats and calponin homology (CH) domain containing 1 [Source:HGNC Symbol;Acc:20309]	3.143210628	2.917734838
ST3GAL1	ST3 beta-galactoside alpha-2,3-sialyltransferase 1 [Source:HGNC Symbol;Acc:10862]	3.295053489	2.91396703
RAC1	ras-related C3 botulinum toxin substrate 1 (rho family, small GTP binding protein Rac1) [Source:HGNC Symbol;Acc:9801]	2.910217072	3.406450678
MMP10	matrix metallopeptidase 10 (stromelysin 2) [Source:HGNC Symbol;Acc:7156]	2.909270776	2.965717393
TGFB1I1	transforming growth factor beta 1 induced transcript 1 [Source:HGNC Symbol;Acc:11767]	3.506700531	2.895517885
CAP2	CAP, adenylate cyclase-associated protein, 2 (yeast) [Source:HGNC Symbol;Acc:20039]	3.474233449	2.893925451
TLL1	tolloid-like 1 [Source:HGNC Symbol;Acc:11843]	3.404711926	2.891127394
C11orf21	chromosome 11 open reading frame 21 [Source:HGNC Symbol;Acc:13231]	2.888256344	3.106834967
ASAH1	N-acylsphingosine amidohydrolase (acid ceramidase) 1 [Source:HGNC Symbol;Acc:735]	3.161971033	2.886899014
SRPX	sushi-repeat containing protein, X-linked [Source:HGNC Symbol;Acc:11309]	2.884197158	3.459290335
FRY	furry homolog (Drosophila) [Source:HGNC Symbol;Acc:20367]	3.23189812	2.868701832
MMP23A	matrix metallopeptidase 23A (pseudogene) [Source:HGNC Symbol;Acc:7170]	2.864373922	3.207075999
TSPAN9	tetraspanin 9 [Source:HGNC Symbol;Acc:21640]	2.854710319	3.440669168
SV2C	synaptic vesicle glycoprotein 2C [Source:HGNC Symbol;Acc:30670]	2.953991902	2.854073427
C7orf41	chromosome 7 open reading frame 41 [Source:HGNC Symbol;Acc:25457]	2.852747202	2.865183937
WNT5B	wingless-type MMTV integration site family, member 5B [Source:HGNC Symbol;Acc:16265]	4.357485501	2.850073122

C4orf26	chromosome 4 open reading frame 26 [Source:HGNC Symbol;Acc:26300]	2.838992113	2.843571499
COL4A1	collagen, type IV, alpha 1 [Source:HGNC Symbol;Acc:2202]	2.839182408	2.83725054
DYSF	dysferlin, limb girdle muscular dystrophy 2B (autosomal recessive) [Source:HGNC Symbol;Acc:3097]	2.836661947	3.498873776
GPR158	G protein-coupled receptor 158 [Source:HGNC Symbol;Acc:23689]	2.831602914	3.135468175
FAM40B	family with sequence similarity 40, member B [Source:HGNC Symbol;Acc:22209]	3.524875809	2.830397097
TPM3	tropomyosin 3 [Source:HGNC Symbol;Acc:12012]	3.19206622	2.830037261
TIPARP	TCDD-inducible poly(ADP-ribose) polymerase [Source:HGNC Symbol;Acc:23696]	3.073091106	2.824921162
ATP2A2	ATPase, Ca++ transporting, cardiac muscle, slow twitch 2 [Source:HGNC Symbol;Acc:812]	2.824051945	2.924371694
MMP11	matrix metallopeptidase 11 (stromelysin 3) [Source:HGNC Symbol;Acc:7157]	2.81412193	2.826774269
FITM1	fat storage-inducing transmembrane protein 1 [Source:HGNC Symbol;Acc:33714]	2.878635414	2.808146673
SLIT3	slit homolog 3 (Drosophila) [Source:HGNC Symbol;Acc:11087]	5.738291503	2.805793018
DNAJC15	DnaJ (Hsp40) homolog, subfamily C, member 15 [Source:HGNC Symbol;Acc:20325]	2.80535935	4.497887154
MTUS2	microtubule associated tumor suppressor candidate 2 [Source:HGNC Symbol;Acc:20595]	3.472164656	2.805041625
ITGA1	integrin, alpha 1 [Source:HGNC Symbol;Acc:6134]	2.801877915	3.114270155
SLC46A3	solute carrier family 46, member 3 [Source:HGNC Symbol;Acc:27501]	3.31789386	2.800809432
PCGF5	polycomb group ring finger 5 [Source:HGNC Symbol;Acc:28264]	3.859591049	2.79899488
ZCCHC24	zinc finger, CCHC domain containing 24 [Source:HGNC Symbol;Acc:26911]	2.7971073	3.196160016
CREM	cAMP responsive element modulator [Source:HGNC Symbol;Acc:2352]	2.796993465	2.919244591
KRT7	keratin 7 [Source:HGNC Symbol;Acc:6445]	2.794013561	5.026917298
DNAJC6	DnaJ (Hsp40) homolog, subfamily C, member 6 [Source:HGNC Symbol;Acc:15469]	3.002105339	2.786817047
ANXA3	annexin A3 [Source:HGNC Symbol;Acc:541]	2.769837017	7.719927506
FLI1	Friend leukemia virus integration 1 [Source:HGNC Symbol;Acc:3749]	2.796466076	2.766734396
CNTN1	contactin 1 [Source:HGNC Symbol;Acc:2171]	2.766411957	4.891983578
KCNE1	potassium voltage-gated channel, Isk-related family, member 1 [Source:HGNC Symbol;Acc:6240]	3.192531136	2.76486461
ADRB2	adrenoceptor beta 2, surface [Source:HGNC Symbol;Acc:286]	2.762816286	2.90277449
PRKAG2	protein kinase, AMP-activated, gamma 2 non-catalytic subunit [Source:HGNC Symbol;Acc:9386]	3.064844421	2.761559696
ADAMTSL1	ADAMTS-like 1 [Source:HGNC Symbol;Acc:14632]	2.761485688	2.807900935
ANXA1	annexin A1 [Source:HGNC Symbol;Acc:533]	7.170799465	2.752470172
LPCAT2	lysophosphatidylcholine acyltransferase 2 [Source:HGNC Symbol;Acc:26032]	3.233037157	2.751663674
NT5E	5'-nucleotidase, ecto (CD73) [Source:HGNC Symbol;Acc:8021]	2.75005365	3.74847992
EMP3	epithelial membrane protein 3 [Source:HGNC Symbol;Acc:3335]	2.747748305	2.966535431
SLC16A12	solute carrier family 16, member 12 (monocarboxylic acid transporter 12) [Source:HGNC Symbol;Acc:23094]	2.747320899	5.705729034
HECW2	HECT, C2 and WW domain containing E3 ubiquitin protein ligase 2 [Source:HGNC Symbol;Acc:29853]	3.397341109	2.738483416
ETV5	ets variant 5 [Source:HGNC Symbol;Acc:3494]	2.737512807	4.596342471
C5orf23	natriuretic peptide receptor C/guanylate cyclase C (atrionatriuretic peptide receptor C) [Source:HGNC Symbol;Acc:7945]	3.193258673	2.733957378
NOTCH2	notch 2 [Source:HGNC Symbol;Acc:7882]	3.141489505	2.732178315
GBE1	glucan (1,4-alpha-), branching enzyme 1 [Source:HGNC Symbol;Acc:4180]	2.731734031	2.881444142
PTP4A3	protein tyrosine phosphatase type IVA, member 3 [Source:HGNC Symbol;Acc:9636]	2.721212174	3.865885502
NFIC	nuclear factor I/C (CCAAT-binding transcription factor) [Source:HGNC Symbol;Acc:7786]	2.718799073	2.846270479
LTBP3	latent transforming growth factor beta binding protein 3 [Source:HGNC Symbol;Acc:6716]	2.718684022	2.91958739
HIST2H2AA3	histone cluster 2, H2aa3 [Source:HGNC Symbol;Acc:4736]	3.927734856	2.71043284
TNNI3	troponin I type 3 (cardiac) [Source:HGNC Symbol;Acc:11947]	2.705739814	5.661829518
NIPAL3	NIPA-like domain containing 3 [Source:HGNC Symbol;Acc:25233]	2.704810791	3.897408884
CEBPD	CCAAT/enhancer binding protein (C/EBP), delta [Source:HGNC Symbol;Acc:1835]	3.64376844	2.697669305
ALPK3	alpha-kinase 3 [Source:HGNC Symbol;Acc:17574]	2.697126068	5.754653531
FIBIN	fin bud initiation factor homolog (zebrafish) [Source:HGNC Symbol;Acc:33747]	2.697069187	2.803376777
KIFAP3	kinesin-associated protein 3 [Source:HGNC Symbol;Acc:17060]	5.123029963	2.693487243
CLIP4	CAP-GLY domain containing linker protein family, member 4 [Source:HGNC Symbol;Acc:26108]	5.394633482	2.682009431
FOSL2	FOS-like antigen 2 [Source:HGNC Symbol;Acc:3798]	4.423555396	2.681173545
LUM	lumican [Source:HGNC Symbol;Acc:6724]	8.733512506	2.679319828
AFAP1L1	actin filament associated protein 1-like 1 [Source:HGNC Symbol;Acc:26714]	2.673182411	3.725859388
KBTBD10	kelch repeat and BTB (POZ) domain containing 10 [Source:HGNC Symbol;Acc:16905]	2.910404942	2.670527553
ITIH2	inter-alpha-trypsin inhibitor heavy chain 2 [Source:HGNC Symbol;Acc:6167]	3.456291265	2.659130726
NTF4	neurotrophin 4 [Source:HGNC Symbol;Acc:8024]	2.656981118	4.251183748
DUSP5	dual specificity phosphatase 5 [Source:HGNC Symbol;Acc:3071]	2.654799588	3.615327677
SPINK1	serine peptidase inhibitor, Kazal type 1 [Source:HGNC Symbol;Acc:11244]	2.654537311	4.62034007
FAM198B	family with sequence similarity 198, member B [Source:HGNC Symbol;Acc:25312]	8.443275189	2.653490933
LOC645954	supervillin pseudogene 1 [Source:HGNC Symbol;Acc:44959]	3.866087216	2.648025391
C1orf198	chromosome 1 open reading frame 198 [Source:HGNC Symbol;Acc:25900]	3.40434572	2.646626383
PYGM	phosphorylase, glycogen, muscle [Source:HGNC Symbol;Acc:9726]	2.644214198	4.018465006
ARHGDIB	Rho GDP dissociation inhibitor (GDI) beta [Source:HGNC Symbol;Acc:679]	2.957175439	2.643826097
HMGCLL1	3-hydroxymethyl-3-methylglutaryl-CoA lyase-like 1 [Source:HGNC Symbol;Acc:21359]	4.458553728	2.642193458
AMY1A	amylase, alpha 1B (salivary) [Source:HGNC Symbol;Acc:475]	2.637543379	2.639898907
LAMA2	laminin, alpha 2 [Source:HGNC Symbol;Acc:6482]	4.1250157	2.633525822
HEG1	HEG homolog 1 (zebrafish) [Source:HGNC Symbol;Acc:29227]	3.525693555	2.632212016
EMP2	epithelial membrane protein 2 [Source:HGNC Symbol;Acc:3334]	2.72255821	2.632176827
TMEM71	transmembrane protein 71 [Source:HGNC Symbol;Acc:26572]	3.095803825	2.631314923
MEF2A	myocyte enhancer factor 2A [Source:HGNC Symbol;Acc:6993]	3.318135518	2.630792529
KCNMA1	potassium large conductance calcium-activated channel, subfamily M, alpha member 1 [Source:HGNC Symbol;Acc:6284]	2.630498738	3.963736336
BHLHE40	basic helix-loop-helix family, member e40 [Source:HGNC Symbol;Acc:1046]	2.618728344	2.858362529
PLAT	plasminogen activator, tissue [Source:HGNC Symbol;Acc:9051]	3.061323078	2.615331978
EEF1A2	eukaryotic translation elongation factor 1 alpha 2 [Source:HGNC Symbol;Acc:3192]	2.612651486	3.017534267
RDH10	retinol dehydrogenase 10 (all-trans) [Source:HGNC Symbol;Acc:19975]	3.689321996	2.607326679
SPARC	secreted protein, acidic, cysteine-rich (osteonectin) [Source:HGNC Symbol;Acc:11219]	2.665645879	2.602676313

COL5A2	collagen, type V, alpha 2 [Source:HGNC Symbol;Acc:2210]	3.378509016	2.599429787
VTN	vitronectin [Source:HGNC Symbol;Acc:12724]	2.701506785	2.599024567
GABRE	gamma-aminobutyric acid (GABA) A receptor, epsilon [Source:HGNC Symbol;Acc:4085]	2.593954096	3.086928895
TNC	tenascin C [Source:HGNC Symbol;Acc:5318]	4.219436562	2.590306334
BMP2	bone morphogenetic protein 2 [Source:HGNC Symbol;Acc:1069]	2.587286651	4.830238418
SHB	Src homology 2 domain containing adaptor protein B [Source:HGNC Symbol;Acc:10838]	2.585851373	3.51979674
IDH3A	isocitrate dehydrogenase 3 (NAD+) alpha [Source:HGNC Symbol;Acc:5384]	2.584052493	2.702302946
PPAPDC1A	phosphatidic acid phosphatase type 2 domain containing 1A [Source:HGNC Symbol;Acc:23531]	2.584200732	2.579158097
NFIA	nuclear factor I/A [Source:HGNC Symbol;Acc:7784]	4.111276698	2.578995125
HHATL	hedgehog acyltransferase-like [Source:HGNC Symbol;Acc:13242]	2.573813156	2.605819453
PPFIA4	protein tyrosine phosphatase, receptor type, f polypeptide (PTPRF), interacting protein (liprin), alpha 4 [Source:HGNC Symbol;Acc:9248]	2.573045521	4.07873122
CACNB1	calcium channel, voltage-dependent, beta 1 subunit [Source:HGNC Symbol;Acc:1401]	2.569133945	2.797106375
EPHB3	EPH receptor B3 [Source:HGNC Symbol;Acc:3394]	3.764781988	2.568780365
TNMD	tenomodulin [Source:HGNC Symbol;Acc:17757]	2.567325173	4.014867628
RARB	retinoic acid receptor, beta [Source:HGNC Symbol;Acc:9865]	4.796149928	2.566751556
LOC728264	MIR143 host gene [Source:HGNC Symbol;Acc:42872]	2.574700118	2.566682262
SMTNL2	smoothelin-like 2 [Source:HGNC Symbol;Acc:24764]	2.562785291	3.221129459
CRIM1	Processed cysteine-rich motor neuron 1 protein [Source:UniProtKB/TrEMBL;Acc:H7C1Z0]	2.562661178	2.575094224
DGKB	diacylglycerol kinase, beta 90kDa [Source:HGNC Symbol;Acc:2850]	2.739386968	2.560779267
PLD1	phospholipase D1, phosphatidylcholine-specific [Source:HGNC Symbol;Acc:9067]	2.648152436	2.554023921
MYOF	myoferlin [Source:HGNC Symbol;Acc:3656]	4.66984565	2.551160105
RAB3IP	RAB3A interacting protein (rabin3) [Source:HGNC Symbol;Acc:16508]	3.44082479	2.549437891
TNS1	tensin 1 [Source:HGNC Symbol;Acc:11973]	2.641474521	2.546919208
TNFRSF11B	tumor necrosis factor receptor superfamily, member 11b [Source:HGNC Symbol;Acc:11909]	2.546140502	2.812419299
PLXNA4	plexin A4 [Source:HGNC Symbol;Acc:9102]	3.430911981	2.540565806
AREG	amphiregulin B [Source:HGNC Symbol;Acc:34509]	2.602307604	2.537663019
DDR2	discoidin domain receptor tyrosine kinase 2 [Source:HGNC Symbol;Acc:2731]	3.112312694	2.53759852
MBNL2	muscleblind-like splicing regulator 2 [Source:HGNC Symbol;Acc:16746]	4.618815759	2.537115197
CLDN1	claudin 1 [Source:HGNC Symbol;Acc:2032]	2.530102806	3.133223146
SERPINB7	serpin peptidase inhibitor, clade B (ovalbumin), member 7 [Source:HGNC Symbol;Acc:13902]	2.779117293	2.522815521
LRRC49	leucine rich repeat containing 49 [Source:HGNC Symbol;Acc:25965]	3.514996157	2.521342547
SIPA1L2	signal-induced proliferation-associated 1 like 2 [Source:HGNC Symbol;Acc:23800]	5.326517398	2.518694226
GPR22	G protein-coupled receptor 22 [Source:HGNC Symbol;Acc:4477]	2.877277776	2.518031814
FBN1	fibrillin 1 [Source:HGNC Symbol;Acc:3603]	3.777926632	2.517590536
LTBP2	latent transforming growth factor beta binding protein 2 [Source:HGNC Symbol;Acc:6715]	2.760352643	2.504542555
WLS	wntless homolog (Drosophila) [Source:HGNC Symbol;Acc:30238]	6.596645997	2.50304107
NAV1	neuron navigator 1 [Source:HGNC Symbol;Acc:15989]	3.008586839	2.497332693
KIFC3	kinesin family member C3 [Source:HGNC Symbol;Acc:6326]	3.039001045	2.494397493
PINK1	PTEN induced putative kinase 1 [Source:HGNC Symbol;Acc:14581]	2.491679005	2.730420915
LRRN4	leucine rich repeat neuronal 4 [Source:HGNC Symbol;Acc:16208]	2.491450066	2.533850351
FHOD3	formin homology 2 domain containing 3 [Source:HGNC Symbol;Acc:26178]	4.005283351	2.488221595
REEP5	receptor accessory protein 5 [Source:HGNC Symbol;Acc:30077]	2.487355532	2.989278033
PIK3AP1	phosphoinositide-3-kinase adaptor protein 1 [Source:HGNC Symbol;Acc:30034]	2.486972061	2.718404066
DNAJB4	DnaJ (Hsp40) homolog, subfamily B, member 4 [Source:HGNC Symbol;Acc:14886]	2.507697012	2.484844348
RAI2	retinoic acid induced 2 [Source:HGNC Symbol;Acc:9835]	2.479067265	2.527148915
ANKRD29	ankyrin repeat domain 29 [Source:HGNC Symbol;Acc:27110]	2.473043879	2.515934989
PCDH10	protocadherin 10 [Source:HGNC Symbol;Acc:13404]	2.472472085	3.436129838
S1PR1	sphingosine-1-phosphate receptor 1 [Source:HGNC Symbol;Acc:3165]	5.443617927	2.472169138
CRIP2	cysteine-rich protein 2 [Source:HGNC Symbol;Acc:2361]	4.575460604	2.470417571
C12orf75	chromosome 12 open reading frame 75 [Source:HGNC Symbol;Acc:35164]	2.46501322	3.115888731
FSD2	fibronectin type III and SPRY domain containing 2 [Source:HGNC Symbol;Acc:18024]	2.773458454	2.460849195
MYH11	myosin, heavy chain 11, smooth muscle [Source:HGNC Symbol;Acc:7569]	2.449936615	2.740463304
KCNH2	potassium voltage-gated channel, subfamily H (eag-related), member 2 [Source:HGNC Symbol;Acc:6251]	2.949892734	2.446804348
C4orf22	chromosome 4 open reading frame 22 [Source:HGNC Symbol;Acc:28554]	2.699260778	2.446799361
PKIG	protein kinase (cAMP-dependent, catalytic) inhibitor gamma [Source:HGNC Symbol;Acc:9019]	2.527671586	2.446752788
RUNX1	runt-related transcription factor 1 [Source:HGNC Symbol;Acc:10471]	3.448153228	2.446637046
AMY2B	amylase, alpha 2B (pancreatic) [Source:HGNC Symbol;Acc:478]	2.44565044	2.521556014
KLHL21	kelch-like 21 (Drosophila) [Source:HGNC Symbol;Acc:29041]	2.443354616	2.446434123
PPM1K	protein phosphatase, Mg2+/Mn2+ dependent, 1K [Source:HGNC Symbol;Acc:25415]	2.588844359	2.441710627
NRP1	neuropilin 1 [Source:HGNC Symbol;Acc:8004]	2.69760084	2.43766035
RGN	regucalcin (senescence marker protein-30) [Source:HGNC Symbol;Acc:9989]	2.43764957	2.979863598
RNF217	ring finger protein 217 [Source:HGNC Symbol;Acc:21487]	2.803458776	2.434558966
LRRC32	leucine rich repeat containing 32 [Source:HGNC Symbol;Acc:4161]	2.432995651	2.898733097
ITGA3	integrin, alpha 3 (antigen CD49C, alpha 3 subunit of VLA-3 receptor) [Source:HGNC Symbol;Acc:6139]	2.432596736	3.447559752
MFAP4	microfibrillar-associated protein 4 [Source:HGNC Symbol;Acc:7035]	2.432324547	2.494674818
CPEB4	cytoplasmic polyadenylation element binding protein 4 [Source:HGNC Symbol;Acc:21747]	3.562931182	2.431663897
CYB5R1	cytochrome b5 reductase 1 [Source:HGNC Symbol;Acc:13397]	2.894761764	2.431505046
SUCNR1	succinate receptor 1 [Source:HGNC Symbol;Acc:4542]	2.426428834	2.527418895
C3orf45	chromosome 3 open reading frame 45 [Source:HGNC Symbol;Acc:26781]	2.426051695	2.746004221
MEF2C	myocyte enhancer factor 2A [Source:HGNC Symbol;Acc:6993]	4.873186024	2.425738741
EPHA2	EPH receptor A2 [Source:HGNC Symbol;Acc:3386]	2.688396111	2.422263399
FXYD1	FXYD domain containing ion transport regulator 1 [Source:HGNC Symbol;Acc:4025]	2.419993843	3.225921016
CYP24A1	cytochrome P450, family 24, subfamily A, polypeptide 1 [Source:HGNC Symbol;Acc:2602]	2.419123217	2.757398216
AGTR1	angiotensin II receptor, type 1 [Source:HGNC Symbol;Acc:336]	2.653599845	2.412591053
NRK	Nik related kinase [Source:HGNC Symbol;Acc:25391]	2.403506377	4.248352806

LAMA4	laminin, alpha 4 [Source:HGNC Symbol;Acc:6484]	3.746126123	2.401208596
OBSCN	obscurin, cytoskeletal calmodulin and titin-interacting RhoGEF [Source:HGNC Symbol;Acc:15719]	2.598541342	2.401037422
GSN	gelsolin [Source:HGNC Symbol;Acc:4620]	2.399892711	3.618107353
RGS4	regulator of G-protein signaling 4 [Source:HGNC Symbol;Acc:10000]	4.096154983	2.398720288
TPD52L1	tumor protein D52-like 1 [Source:HGNC Symbol;Acc:12006]	2.397345114	6.008245637
PIK3IP1	phosphoinositide-3-kinase interacting protein 1 [Source:HGNC Symbol;Acc:24942]	2.624510026	2.394294113
KCNMB1	potassium large conductance calcium-activated channel, subfamily M, beta member 1 [Source:HGNC Symbol;Acc:6285]	2.391409626	2.767254811
SLC4A3	solute carrier family 4, anion exchanger, member 3 [Source:HGNC Symbol;Acc:11029]	2.48130215	2.38933331
GJB2	gap junction protein, beta 2, 26kDa [Source:HGNC Symbol;Acc:4284]	3.581366669	2.38918059
HIST1H2BC	histone cluster 1, H2bc [Source:HGNC Symbol;Acc:4757]	2.387947011	2.405709082
COL16A1	collagen, type XVI, alpha 1 [Source:HGNC Symbol;Acc:2193]	2.386694542	2.733669244
FILIP1L	filamin A interacting protein 1-like [Source:HGNC Symbol;Acc:24589]	5.578265841	2.378232274
ITGA9	integrin, alpha 9 [Source:HGNC Symbol;Acc:6145]	3.648124556	2.373465137
DNAJB9	DnaJ (Hsp40) homolog, subfamily B, member 9 [Source:HGNC Symbol;Acc:6968]	2.372232699	2.608504436
CHPF	chondroitin polymerizing factor [Source:HGNC Symbol;Acc:24291]	2.371691788	2.624657229
KLF5	Kruppel-like factor 5 (intestinal) [Source:HGNC Symbol;Acc:6349]	2.369880741	2.725423568
HTRA1	HtrA serine peptidase 1 [Source:HGNC Symbol;Acc:9476]	4.13356547	2.366230258
PLAC8	placenta-specific 8 [Source:HGNC Symbol;Acc:19254]	2.365435004	3.297954212
NOTCH2NL	notch 2 N-terminal like [Source:HGNC Symbol;Acc:31862]	2.564634882	2.365392364
SPTB	spectrin, beta, erythrocytic [Source:HGNC Symbol;Acc:11274]	2.365841866	2.352369594
TOM1L2	target of myb1-like 2 (chicken) [Source:HGNC Symbol;Acc:11984]	4.122592757	2.347589042
SLC47A1	solute carrier family 47, member 1 [Source:HGNC Symbol;Acc:25588]	2.34630636	2.954455606
RAPGEF2	Rap guanine nucleotide exchange factor (GEF) 2 [Source:HGNC Symbol;Acc:16854]	2.832073402	2.343758812
SH3GL3	SH3-domain GRB2-like 3 [Source:HGNC Symbol;Acc:10832]	2.340150365	5.194960145
DYNC1I1	dynein, cytoplasmic 1, intermediate chain 1 [Source:HGNC Symbol;Acc:2963]	2.339263907	2.819607679
BMP1	bone morphogenetic protein 1 [Source:HGNC Symbol;Acc:1067]	3.014227605	2.337648181
RCAN1	regulator of calcineurin 1 [Source:HGNC Symbol;Acc:3040]	3.840867349	2.335225437
IL11RA	interleukin 11 receptor, alpha [Source:HGNC Symbol;Acc:5967]	2.961026924	2.334197306
ESRRG	estrogen-related receptor gamma [Source:HGNC Symbol;Acc:3474]	3.481058905	2.333943976
FNDC3B	fibronectin type III domain containing 3B [Source:HGNC Symbol;Acc:24670]	2.42896011	2.330817762
GABRB2	gamma-aminobutyric acid (GABA) A receptor, beta 2 [Source:HGNC Symbol;Acc:4082]	2.330433789	2.496642512
MYO18A	myosin XVIIIA [Source:HGNC Symbol;Acc:31104]	2.481218089	2.330270541
CAMK2B	calcium/calmodulin-dependent protein kinase II beta [Source:HGNC Symbol;Acc:1461]	2.599947171	2.327674682
ALAS2	aminolevulinate, delta-, synthase 2 [Source:HGNC Symbol;Acc:397]	2.36416665	2.321688083
ADORA1	adenosine A1 receptor [Source:HGNC Symbol;Acc:262]	2.320413999	2.856332214
ST3GAL6	ST3 beta-galactoside alpha-2,3-sialyltransferase 6 [Source:HGNC Symbol;Acc:18080]	2.317131238	3.313021541
NIPAL2	NIPA-like domain containing 2 [Source:HGNC Symbol;Acc:25854]	2.315313141	3.081663593
MICALCL	MICAL C-terminal like [Source:HGNC Symbol;Acc:25933]	2.314163621	2.314131279
NPY6R	neuropeptide Y receptor Y6 (pseudogene) [Source:HGNC Symbol;Acc:7959]	2.533402031	2.303925424
DPF3	D4, zinc and double PHD fingers, family 3 [Source:HGNC Symbol;Acc:17427]	2.330159124	2.297639325
GADD45A	growth arrest and DNA-damage-inducible, alpha [Source:HGNC Symbol;Acc:4095]	2.629543878	2.293602718
ISL1	ISL LIM homeobox 1 [Source:HGNC Symbol;Acc:6132]	3.566077192	2.292955142
P4HA2	prolyl 4-hydroxylase, alpha polypeptide II [Source:HGNC Symbol;Acc:8547]	2.2906579	3.382339652
HOXC6	homeobox C6 [Source:HGNC Symbol;Acc:5128]	2.298448122	2.289346408
EDN3	endothelin 3 [Source:HGNC Symbol;Acc:3178]	2.289270913	2.348828147
HSBP1L1	heat shock factor binding protein 1-like 1 [Source:HGNC Symbol;Acc:37243]	2.288523359	2.464323206
ARHGAP1	Rho GTPase activating protein 1 [Source:HGNC Symbol;Acc:673]	2.287345087	2.586896318
OLFML1	olfactomedin-like 1 [Source:HGNC Symbol;Acc:24473]	2.733435173	2.284483834
GATA3	GATA binding protein 3 [Source:HGNC Symbol;Acc:4172]	2.488925452	2.282521451
TRIM54	tripartite motif containing 54 [Source:HGNC Symbol;Acc:16008]	2.281271808	2.753741378
FAM43A	family with sequence similarity 43, member A [Source:HGNC Symbol;Acc:26888]	2.278824178	4.117270172
FAM114A1	family with sequence similarity 114, member A1 [Source:HGNC Symbol;Acc:25087]	2.277625262	2.316076259
C12orf39	chromosome 12 open reading frame 39 [Source:HGNC Symbol;Acc:28139]	2.275078059	2.823024651
TNFRSF12A	tumor necrosis factor receptor superfamily, member 12A [Source:HGNC Symbol;Acc:18152]	2.274744111	5.473612315
PPAPDC3	phosphatidic acid phosphatase type 2 domain containing 3 [Source:HGNC Symbol;Acc:28174]	2.542112063	2.271834859
HSPB1	heat shock 27kDa protein 1 [Source:HGNC Symbol;Acc:5246]	2.270522749	3.522735401
P2RX1	purinergic receptor P2X, ligand-gated ion channel, 1 [Source:HGNC Symbol;Acc:8533]	2.267513004	2.269241321
GPR87	G protein-coupled receptor 87 [Source:HGNC Symbol;Acc:4538]	2.527927796	2.265925459
ARHGAP24	Rho GTPase activating protein 24 [Source:HGNC Symbol;Acc:25361]	3.020766115	2.263093956
COL1A2	collagen, type I, alpha 2 [Source:HGNC Symbol;Acc:2198]	2.485941066	2.261964288
ABLIM1	actin binding LIM protein 1 [Source:HGNC Symbol;Acc:78]	2.257898689	2.653017499
WNT11	wingless-type MMTV integration site family, member 11 [Source:HGNC Symbol;Acc:12776]	2.257796944	2.322254015
IL17B	interleukin 17B [Source:HGNC Symbol;Acc:5982]	2.257117338	2.445549648
DES	desmin [Source:HGNC Symbol;Acc:2770]	2.256009807	2.296948768
SCP2	sterol carrier protein 2 [Source:HGNC Symbol;Acc:10606]	2.547423401	2.252429705
CTSB	cathepsin B [Source:HGNC Symbol;Acc:2527]	2.96519629	2.251876805
SRL	sarcalumenin [Source:HGNC Symbol;Acc:11295]	2.48872319	2.251483202
KRT18	keratin 18 [Source:HGNC Symbol;Acc:6430]	2.251329725	5.206768591
AEBP1	AE binding protein 1 [Source:HGNC Symbol;Acc:303]	2.241638587	2.596430693
NTN4	netrin 4 [Source:HGNC Symbol;Acc:13658]	4.847453201	2.239017681
HAND1	heart and neural crest derivatives expressed 1 [Source:HGNC Symbol;Acc:4807]	2.268566842	2.238637115
TINAGL1	tubulointerstitial nephritis antigen-like 1 [Source:HGNC Symbol;Acc:19168]	2.237202304	2.664746116
CHCHD10	coiled-coil-helix-coiled-coil-helix domain containing 10 [Source:HGNC Symbol;Acc:15559]	2.235225793	3.026886444
SEMA5A	sema domain, seven thrombospondin repeats (type 1 and type 1-like), transmembrane domain (TM) and short cytoplasmic domain, (semaphorin) 5A [Source:HGNC Symbol;Acc:10736]	2.234909804	2.364783174
RHOC	ras homolog family member C [Source:HGNC Symbol;Acc:669]	2.629222146	2.22772206

SIK1	salt-inducible kinase 1 [Source:HGNC Symbol;Acc:11142]	2.217898721	2.462259226
LYPD6B	LY6/PLAUR domain containing 6B [Source:HGNC Symbol;Acc:27018]	2.215989968	3.835385502
TFPI2	tissue factor pathway inhibitor 2 [Source:HGNC Symbol;Acc:11761]	2.472962293	2.21139321
CPNE5	copine V [Source:HGNC Symbol;Acc:2318]	2.210552425	2.334982429
FGF18	fibroblast growth factor 18 [Source:HGNC Symbol;Acc:3674]	4.055660823	2.207151699
BDNF	brain-derived neurotrophic factor [Source:HGNC Symbol;Acc:1033]	2.206718462	4.715377223
TANC1	tetratricopeptide repeat, ankyrin repeat and coiled-coil containing 1 [Source:HGNC Symbol;Acc:29364]	3.119489226	2.204827172
SGCA	sarcoglycan, alpha (50kDa dystrophin-associated glycoprotein) [Source:HGNC Symbol;Acc:10805]	2.271977237	2.204772184
C13orf1	chromosome 13 open reading frame 1 [Source:HGNC Symbol;Acc:14297]	2.277928352	2.201910776
CCDC69	coiled-coil domain containing 69 [Source:HGNC Symbol;Acc:24487]	2.200240456	3.724009502
EFNA1	ephrin-A1 [Source:HGNC Symbol;Acc:3221]	2.329725627	2.199268696
DACT3	dapper, antagonist of beta-catenin, homolog 3 (Xenopus laevis) [Source:HGNC Symbol;Acc:30745]	3.666765448	2.194030857
FZD8	frizzled family receptor 8 [Source:HGNC Symbol;Acc:4046]	2.187670726	3.019546569
BTK	Bruton agammaglobulinemia tyrosine kinase [Source:HGNC Symbol;Acc:1133]	2.185687411	2.182037875
KLF2	Kruppel-like factor 2 (lung) [Source:HGNC Symbol;Acc:6347]	2.274460531	2.1802963
AKAP2	A kinase (PRKA) anchor protein 2 [Source:HGNC Symbol;Acc:372]	2.856165807	2.17994355
NCRNA00158	uncharacterized	2.388044792	2.17863794
FAM20A	family with sequence similarity 20, member A [Source:HGNC Symbol;Acc:23015]	2.176183979	2.758230688
FAM189A2	family with sequence similarity 189, member A2 [Source:HGNC Symbol;Acc:24820]	2.174781241	4.471164725
DCAF6	DDB1 and CUL4 associated factor 6 [Source:HGNC Symbol;Acc:30002]	2.170776283	2.254407007
WIPI1	WD repeat domain, phosphoinositide interacting 1 [Source:HGNC Symbol;Acc:25471]	2.168329191	3.431342244
LOC730755	keratin associated protein 2-4-like [Source:HGNC Symbol;Acc:18891]	2.184088013	2.167268265
COL4A4	collagen, type IV, alpha 4 [Source:HGNC Symbol;Acc:2206]	2.166842616	2.998745166
ARHGAP29	Rho GTPase activating protein 29 [Source:HGNC Symbol;Acc:30207]	2.671035273	2.164680363
DYNLT3	dynein, light chain, Tctex-type 3 [Source:HGNC Symbol;Acc:11694]	2.163982702	2.707940592
DHRS9	dehydrogenase/reductase (SDR family) member 9 [Source:HGNC Symbol;Acc:16888]	2.275631348	2.15956003
SLC7A7	solute carrier family 7 (amino acid transporter light chain, y+L system), member 7 [Source:HGNC Symbol;Acc:11065]	2.150410942	3.650493919
VWC2	von Willebrand factor C domain containing 2 [Source:HGNC Symbol;Acc:30200]	2.149940383	2.729170841
SLITRK6	SLIT and NTRK-like family, member 6 [Source:HGNC Symbol;Acc:23503]	2.639825079	2.149133322
PAM	peptidylglycine alpha-amidating monooxygenase [Source:HGNC Symbol;Acc:8596]	3.028789138	2.147368549
LMNA	lamin A/C [Source:HGNC Symbol;Acc:6636]	2.146124785	2.181333329
SLC25A4	solute carrier family 25 (mitochondrial carrier; adenine nucleotide translocator), member 4 [Source:HGNC Symbol;Acc:10990]	2.145499083	2.879252653
JAZF1	JAZF zinc finger 1 [Source:HGNC Symbol;Acc:28917]	2.14123603	2.266995089
USP2	ubiquitin specific peptidase 2 [Source:HGNC Symbol;Acc:12618]	2.138104919	2.953795768
FAM47E	family with sequence similarity 47, member E [Source:HGNC Symbol;Acc:34343]	2.137806377	2.916606692
CAMK2A	calcium/calmodulin-dependent protein kinase II alpha [Source:HGNC Symbol;Acc:1460]	2.444858163	2.136153848
HSPG2	heparan sulfate proteoglycan 2 [Source:HGNC Symbol;Acc:5273]	2.134351211	3.172611516
COL11A1	collagen, type XI, alpha 1 [Source:HGNC Symbol;Acc:2186]	2.131036355	2.423135945
MYADM	myeloid-associated differentiation marker [Source:HGNC Symbol;Acc:7544]	2.129387658	2.96665991
TEX2	testis expressed 2 [Source:HGNC Symbol;Acc:30884]	2.151042802	2.128754626
SP6	Sp6 transcription factor [Source:HGNC Symbol;Acc:14530]	2.127316535	2.636213505
ASB11	ankyrin repeat and SOCS box containing 11 [Source:HGNC Symbol;Acc:17186]	2.30666701	2.124774125
NID2	nidogen 2 (osteonidogen) [Source:HGNC Symbol;Acc:13389]	2.12380963	3.301707103
ARHGEF37	Rho guanine nucleotide exchange factor (GEF) 37 [Source:HGNC Symbol;Acc:34430]	2.129062188	2.122950075
EGF	epidermal growth factor [Source:HGNC Symbol;Acc:3229]	2.122317352	2.397806043
PRKAR1A	protein kinase, cAMP-dependent, regulatory, type I, alpha [Source:HGNC Symbol;Acc:9388]	2.44528867	2.11767231
C10orf54	chromosome 10 open reading frame 54 [Source:HGNC Symbol;Acc:30085]	2.177170187	2.116499535
AMBP	alpha-1-microglobulin/bikunin precursor [Source:HGNC Symbol;Acc:453]	2.114969834	2.497977547
TRAK2	trafficking protein, kinesin binding 2 [Source:HGNC Symbol;Acc:13206]	2.309900276	2.114774492
LAYN	layilin [Source:HGNC Symbol;Acc:29471]	2.112141387	3.232875442
FOXP1	forkhead box P1 [Source:HGNC Symbol;Acc:3823]	2.109816386	2.178936106
PTPRM	protein tyrosine phosphatase, receptor type, M [Source:HGNC Symbol;Acc:9675]	2.628340304	2.108680309
PLP2	proteolipid protein 2 (colonic epithelium-enriched) [Source:HGNC Symbol;Acc:9087]	2.102497981	4.145450962
PMEPA1	prostate transmembrane protein, androgen induced 1 [Source:HGNC Symbol;Acc:14107]	2.101951829	2.239031374
PION	pigeon homolog (Drosophila) [Source:HGNC Symbol;Acc:28042]	2.098167055	2.667599314
MALAT1	metastasis associated lung adenocarcinoma transcript 1 (non-protein coding) [Source:HGNC Symbol;Acc:29665]	3.76810076	2.095856878
MYOT	myotilin [Source:HGNC Symbol;Acc:12399]	2.31969309	2.095311407
SEMA3D	sema domain, immunoglobulin domain (Ig), short basic domain, secreted, (semaphorin) 3D [Source:HGNC Symbol;Acc:10726]	2.755605727	2.093991899
SORCS3	sortilin-related VPS10 domain containing receptor 3 [Source:HGNC Symbol;Acc:16699]	4.738306416	2.093959385
COPZ2	coatomer protein complex, subunit zeta 2 [Source:HGNC Symbol;Acc:19356]	2.265703995	2.090999397
TMEM178	transmembrane protein 178A [Source:HGNC Symbol;Acc:28517]	2.478827924	2.08935444
NEURL2	neuralized homolog 2 (Drosophila) [Source:HGNC Symbol;Acc:16156]	2.084726643	2.258787753
ME3	malic enzyme 3, NADP(+)-dependent, mitochondrial [Source:HGNC Symbol;Acc:6985]	2.080714637	2.33256751
NUDT4	nudix (nucleoside diphosphate linked moiety X)-type motif 4 [Source:HGNC Symbol;Acc:8051]	2.069832963	2.627299837
EFHD1	EF-hand domain family, member D1 [Source:HGNC Symbol;Acc:29556]	2.403761277	2.068563885
NOSTRIN	nitric oxide synthase trafficker [Source:HGNC Symbol;Acc:20203]	2.068551409	2.068792514
UACA	uveal autoantigen with coiled-coil domains and ankyrin repeats [Source:HGNC Symbol;Acc:15947]	2.50137594	2.065944243
CHRM2	cholinergic receptor, muscarinic 2 [Source:HGNC Symbol;Acc:1951]	2.070501125	2.061829668
CCPG1	cell cycle progression 1 [Source:HGNC Symbol;Acc:24227]	2.626527109	2.060469769
PROS1	protein S (alpha) [Source:HGNC Symbol;Acc:9456]	4.388856141	2.059171301
LOXL1	lysyl oxidase-like 1 [Source:HGNC Symbol;Acc:6665]	2.055112963	2.077978526
CPA4	carboxypeptidase A4 [Source:HGNC Symbol;Acc:15740]	3.5421202	2.04960982

SLC6A13	solute carrier family 6 (neurotransmitter transporter, GABA), member 13 [Source:HGNC Symbol;Acc:11046]	2.709643237	2.044170594
ROGDI	rogdi homolog (Drosophila) [Source:HGNC Symbol;Acc:29478]	2.051879251	2.041283918
C8orf49	chromosome 8 open reading frame 49 [Source:HGNC Symbol;Acc:32200]	2.157288026	2.040895542
ADAM9	ADAM metallopeptidase domain 9 [Source:HGNC Symbol;Acc:216]	2.539942579	2.040440527
SETD7	SET domain containing (lysine methyltransferase) 7 [Source:HGNC Symbol;Acc:30412]	3.511250581	2.039974523
ARHGAP31	Rho GTPase activating protein 31 [Source:HGNC Symbol;Acc:29216]	2.205351184	2.0382251
SSPN	sarcospan [Source:HGNC Symbol;Acc:11322]	2.597203235	2.038042524
TMEM88	transmembrane protein 88 [Source:HGNC Symbol;Acc:32371]	2.0360148	3.047439612
SERPINA3	serpin peptidase inhibitor, clade A (alpha-1 antiproteinase, antitrypsin), member 3 [Source:HGNC Symbol;Acc:16]	2.034981572	2.03900297
LAMC1	laminin, gamma 1 (formerly LAMB2) [Source:HGNC Symbol;Acc:6492]	2.034062537	2.958646804
ADAMTS12	ADAM metallopeptidase with thrombospondin type 1 motif, 12 [Source:HGNC Symbol;Acc:14605]	2.145003904	2.032026332
N4BP2L2	NEDD4 binding protein 2-like 2 [Source:HGNC Symbol;Acc:26916]	3.587936473	2.030876613
CYR61	cysteine-rich, angiogenic inducer, 61 [Source:HGNC Symbol;Acc:2654]	2.854504257	2.029073797
TFAP2A	transcription factor AP-2 alpha (activating enhancer binding protein 2 alpha) [Source:HGNC Symbol;Acc:11742]	2.86417953	2.024668214
SLC20A2	solute carrier family 20 (phosphate transporter), member 2 [Source:HGNC Symbol;Acc:10947]	2.660372946	2.020731869
ARHGEF9	Cdc42 guanine nucleotide exchange factor (GEF) 9 [Source:HGNC Symbol;Acc:14561]	2.044891936	2.020660997
CRIP1	cysteine-rich protein 1 (intestinal) [Source:HGNC Symbol;Acc:2360]	2.020100094	2.971726462
DUSP3	dual specificity phosphatase 3 [Source:HGNC Symbol;Acc:3069]	2.725743002	2.019618218
ST8SIA5	ST8 alpha-N-acetyl-neuraminide alpha-2,8-sialyltransferase 5 [Source:HGNC Symbol;Acc:17827]	2.015553998	2.493493772
AK2	adenylate kinase 2 [Source:HGNC Symbol;Acc:362]	2.383000821	2.014802141
PPFIBP1	PTPRF interacting protein, binding protein 1 (liprin beta 1) [Source:HGNC Symbol;Acc:9249]	2.014107575	2.333078879
MCC	mutated in colorectal cancers [Source:HGNC Symbol;Acc:6935]	2.587639009	2.013572712
SGMS1	sphingomyelin synthase 1 [Source:HGNC Symbol;Acc:29799]	2.012310517	2.696522529
MSX2	msh homeobox 2 [Source:HGNC Symbol;Acc:7392]	4.015808835	2.011075612
ANXA2	annexin A2 [Source:HGNC Symbol;Acc:537]	2.720696227	2.00845674
PGPEP1	pyroglutamyl-peptidase I [Source:HGNC Symbol;Acc:13568]	2.005562033	2.96953967
ADCY6	adenylate cyclase 6 [Source:HGNC Symbol;Acc:237]	2.179293559	2.004790283
LIMS1	LIM and senescent cell antigen-like domains 1 [Source:HGNC Symbol;Acc:6616]	2.308084787	2.004035749
IL13RA1	interleukin 13 receptor, alpha 1 [Source:HGNC Symbol;Acc:5974]	2.374856357	2.001877374
ABTB2	ankyrin repeat and BTB (POZ) domain containing 2 [Source:HGNC Symbol;Acc:23842]	2.018842402	2.001410566
