# Supplementary material for: Global DNA methylation and transcriptional analyses of human ESC-derived cardiomyocytes
Source: Protein Cell. 2014 Jan 29;5(1):59–68. doi: 10.1007/s13238-013-0016-x (PMC3938846; doi:10.1007/s13238-013-0016-x)
Supplement: Supplementary file 9 — Table S6: GO analysis of hCM differentially down-regulated genes-Biological Process. This file contains the list of GO terms in the category of biological process that were significantly (p<0.05) overrepresented among the 401 hCM differentially downregulatedgenes [file 13238_2013_16_MOESM9_ESM.pdf]

| Gene | Protein | Diseases | Mutations in DNA or protein seqence | References |
|---|---|---|---|---|
| PLN | Phospholamban | HCM | -42C>G, -77A>G | 1, 2 |
| | | DCM | R9C, R14del | 3, 4 |
| MYH6 | myosin-6 | HCM or DCM | R795Q, P830L, A1004S, Q1065H, E1457K | 5, 6 |
| | | ASD | I820N | 7 |
| MYOZ2 | myozenin-2 | HCM | S48P, I246M | 8 |
| MYL2 | myosin regulatory light chain 2, ventricular/cardiac muscle isoform | HCM | A13T, F18L, E22K, N47K, R58Q, K103E, IVS6-1G>C | 9, 10 |
| CSRP3 | cysteine and glycine-rich protein 3 | HCM | L44P, S54R/E55G, C58G, 172T>G | 11, 12 |
| | | DCM | W4R | 13 |
| TTN | titin | HCM | R740L, S3799Y | 14 |
| | | DCM | V54M, A743V, W930R, R25618Q, Q4053ter, S4465D, 2-bp insertion in exon 326 | 14-16, |
| LDB3 | LIM domain binding 3 | DCM | D117N, K136M, S196L, T213I, I352M, D626N | 17, 18 |
| | | LVNC | D117N, K136M, S196L | 17 |
| MYH7 | myosin-7 | HCM | P211L, L390V, R403W, R403Q, R453C, Y501C, H576R, R663C, Q734P, I736T, G741W, A797T, R870H, A901G, D928N, E930K, R1053Q, E1356K, A1379T, A1454T, R1500W, S1776G | 19-24 |
| | | DCM | I201T, T412N, A550V, T1019N, R1193S, E1426K, R1634S | 25 |
| | | LVNC | M531R | 26 |
| ANKRD1 | Ankyrin repeat domain-containing protein 1 | TAPVR | 10;21 translocation | 27 |
| NPPB | natriuretic peptides B preproprotein | HCM | D175N | 28 |
| | | EH | Variable number of tandem repeat of 5'-flanking region | 29 |
| TNNT2 | troponin T type 2 | DCM | I79N, R141W, K210del | 25, 30-32 |
| | | HCM | I79N, I90M, R92C, R92W, F110I, K124N, R130C, R278C, G287A, Δexon 14, a five-base (CTTCT) insertion/deletion in intron 3 | 19, 23, 32-37 |
| | | RCM | I79N, Q96del | 32, 38 |

| | | | | |
|---|---|---|---|---|
| NPPA | natriuretic peptides A preproprotein | Ischemic Stroke | G664A | 39, 40 |
| | | AF | c.456–457delAA | 41 |
| | | EH | V7M, –C664G, G1837A, T2238C | 42, 43 |
| | | LVH | -2843A>G | 44 |
| TNNC1 | Troponin C type 1 | HCM | A8V, L29Q, A31S, C84Y, E122A, E134D, D145E | 45-47 |
| | | DCM | Y5H, M103I, D145E, I148V | 48 |
| ACTN2 | alpha-actinin-2 | DCM | Q9R | 49 |
| MYL3 | myosin light chain 3 | HCM | D778E | 50 |
| GATA4 | transcription factor GATA-4 | CHD | A6V, S52F, P163S, E216D, E359K, S377G, P407Q, S429T, A442V, IVS4+55C>A, c.1074delC | 51-55 |
| RYR2 | ryanodine receptor 2 | CPVT | R414C, E1724K, A2254V, R2267H, A2394G, V2475F, F4020L, E4076K, N4104I, N4104K, H4108N, H4108Q, R4497C, S4565R, G4662S, H4762P, V4771I, P4902S, genomic deletion involved RYR2 exon-3 | 56-60 |
| MYOCD | myocardin | CHD | K259R | 61 |
| TRIM63 | E3 ubiquitin-protei n ligase TRIM63 | HCM | A48V, I130M, Q247del | 62 |

DCM Dilated Cardiomyopathy
HCM Hypertrophic Cardiomyopathy
RCM Restrictive Cardiomyopathy
ASD Atrial Septal Defect
LVNC Left Ventricular Non-Compaction
TAPVR Total Anomalous Pulmonary Venous Return
EH Essential Hypertension
AF Atrial Fibrillation
LVH Left Ventricular Hypertrophy
CPVT Catecholaminergic Polymorphic Ventricular Tachycardia
CHD Congenital Heart Disease
ARVC Arrhythmogenic Right Ventricular Cardiomyopathy

**References**:

1 Minamisawa S, Sato Y, Tatsuguchi Y *et al.* Mutation of the phospholamban promoter associated with hypertrophic cardiomyopathy. *Biochemical and biophysical research communications* 2003;

**304**:1-4.
2 Medin M, Hermida-Prieto M, Monserrat L *et al.* Mutational screening of phospholamban gene in hypertrophic and idiopathic dilated cardiomyopathy and functional study of the PLN -42 C>G mutation. *European journal of heart failure* 2007; **9**:37-43.
3 Schmitt JP, Kamisago M, Asahi M *et al.* Dilated cardiomyopathy and heart failure caused by a mutation in phospholamban. *Science* 2003; **299**:1410-1413.
4 Haghighi K, Kolokathis F, Gramolini AO *et al.* A mutation in the human phospholamban gene, deleting arginine 14, results in lethal, hereditary cardiomyopathy. *Proceedings of the National Academy of Sciences of the United States of America* 2006; **103**:1388-1393.
5 Niimura H, Patton KK, McKenna WJ *et al.* Sarcomere protein gene mutations in hypertrophic cardiomyopathy of the elderly. *Circulation* 2002; **105**:446-451.
6 Carniel E, Taylor MR, Sinagra G *et al.* Alpha-myosin heavy chain: a sarcomeric gene associated with dilated and hypertrophic phenotypes of cardiomyopathy. *Circulation* 2005; **112**:54-59.
7 Ching YH, Ghosh TK, Cross SJ *et al.* Mutation in myosin heavy chain 6 causes atrial septal defect. *Nature genetics* 2005; **37**:423-428.
8 Osio A, Tan L, Chen SN *et al.* Myozenin 2 is a novel gene for human hypertrophic cardiomyopathy. *Circulation research* 2007; **100**:766-768.
9 Flavigny J, Richard P, Isnard R *et al.* Identification of two novel mutations in the ventricular regulatory myosin light chain gene (MYL2) associated with familial and classical forms of hypertrophic cardiomyopathy. *Journal of molecular medicine* 1998; **76**:208-214.
10 Morner S, Richard P, Kazzam E *et al.* Identification of the genotypes causing hypertrophic cardiomyopathy in northern Sweden. *Journal of molecular and cellular cardiology* 2003; **35**:841-849.
11 Geier C, Gehmlich K, Ehler E *et al.* Beyond the sarcomere: CSRP3 mutations cause hypertrophic cardiomyopathy. *Human molecular genetics* 2008; **17**:2753-2765.
12 Geier C, Perrot A, Ozcelik C *et al.* Mutations in the human muscle LIM protein gene in families with hypertrophic cardiomyopathy. *Circulation* 2003; **107**:1390-1395.
13 Knoll R, Hoshijima M, Hoffman HM *et al.* The cardiac mechanical stretch sensor machinery involves a Z disc complex that is defective in a subset of human dilated cardiomyopathy. *Cell* 2002; **111**:943-955.
14 Matsumoto Y, Hayashi T, Inagaki N *et al.* Functional analysis of titin/connectin N2-B mutations found in cardiomyopathy. *Journal of muscle research and cell motility* 2005; **26**:367-374.
15 Itoh-Satoh M, Hayashi T, Nishi H *et al.* Titin mutations as the molecular basis for dilated cardiomyopathy. *Biochemical and biophysical research communications* 2002; **291**:385-393.
16 Gerull B, Gramlich M, Atherton J *et al.* Mutations of TTN, encoding the giant muscle filament titin, cause familial dilated cardiomyopathy. *Nature genetics* 2002; **30**:201-204.
17 Vatta M, Mohapatra B, Jimenez S *et al.* Mutations in Cypher/ZASP in patients with dilated cardiomyopathy and left ventricular non-compaction. *Journal of the American College of Cardiology* 2003; **42**:2014-2027.
18 Arimura T, Hayashi T, Terada H *et al.* A Cypher/ZASP mutation associated with dilated cardiomyopathy alters the binding affinity to protein kinase C. *The Journal of biological chemistry* 2004; **279**:6746-6752.
19 Revera M, van der Merwe L, Heradien M *et al.* Troponin T and beta-myosin mutations have distinct cardiac functional effects in hypertrophic cardiomyopathy patients without hypertrophy. *Cardiovascular research* 2008; **77**:687-694.
20 Havndrup O, Bundgaard H, Andersen PS *et al.* A novel missense mutation, Leu390Val, in the cardiac beta-myosin heavy chain associated with pronounced septal hypertrophy in two families

with hypertrophic cardiomyopathy. *Scandinavian cardiovascular journal : SCJ* 2000; **34**:558-563.
21 Blair E, Redwood C, de Jesus Oliveira M *et al.* Mutations of the light meromyosin domain of the beta-myosin heavy chain rod in hypertrophic cardiomyopathy. *Circulation research* 2002; **90**:263-269.
22 Perrot A, Schmidt-Traub H, Hoffmann B *et al.* Prevalence of cardiac beta-myosin heavy chain gene mutations in patients with hypertrophic cardiomyopathy. *Journal of molecular medicine* 2005; **83**:468-477.
23 Song L, Zou Y, Wang J *et al.* Mutations profile in Chinese patients with hypertrophic cardiomyopathy. *Clinica chimica acta; international journal of clinical chemistry* 2005; **351**:209-216.
24 Bashyam MD, Savithri GR, Gopikrishna M, Narasimhan C. A p.R870H mutation in the beta-cardiac myosin heavy chain 7 gene causes familial hypertrophic cardiomyopathy in several members of an Indian family. *The Canadian journal of cardiology* 2007; **23**:788-790.
25 Villard E, Duboscq-Bidot L, Charron P *et al.* Mutation screening in dilated cardiomyopathy: prominent role of the beta myosin heavy chain gene. *European heart journal* 2005; **26**:794-803.
26 Kaneda T, Naruse C, Kawashima A *et al.* A novel beta-myosin heavy chain gene mutation, p.Met531Arg, identified in isolated left ventricular non-compaction in humans, results in left ventricular hypertrophy that progresses to dilation in a mouse model. *Clinical science* 2008; **114**:431-440.
27 Cinquetti R, Badi I, Campione M *et al.* Transcriptional deregulation and a missense mutation define ANKRD1 as a candidate gene for total anomalous pulmonary venous return. *Human mutation* 2008; **29**:468-474.
28 Magga J, Sipola P, Vuolteenaho O *et al.* Significance of plasma levels of N-terminal Pro-B-type natriuretic peptide on left ventricular remodeling in non-obstructive hypertrophic cardiomyopathy attributable to the Asp175Asn mutation in the alpha-tropomyosin gene. *The American journal of cardiology* 2008; **101**:1185-1190.
29 Kosuge K, Soma M, Nakayama T *et al.* A novel variable number of tandem repeat of the natriuretic peptide precursor B gene's 5'-flanking region is associated with essential hypertension among Japanese females. *International journal of medical sciences* 2007; **4**:146-152.
30 Li D, Czernuszewicz GZ, Gonzalez O *et al.* Novel cardiac troponin T mutation as a cause of familial dilated cardiomyopathy. *Circulation* 2001; **104**:2188-2193.
31 Martins E, Silva-Cardoso J, Alves C *et al.* Familial dilated cardiomyopathy with troponin T K210del mutation. *Revista portuguesa de cardiologia : orgao oficial da Sociedade Portuguesa de Cardiologia = Portuguese journal of cardiology : an official journal of the Portuguese Society of Cardiology* 2006; **25**:295-300.
32 Menon SC, Michels VV, Pellikka PA *et al.* Cardiac troponin T mutation in familial cardiomyopathy with variable remodeling and restrictive physiology. *Clinical genetics* 2008; **74**:445-454.
33 Cuda G, Mussari A, Concolino D, Costanzo FS, Strisciuglio P. Co-existence of frataxin and cardiac troponin T gene mutations in a child with Friedreich Ataxia and familial hypertrophic cardiomyopathy. *Human mutation* 2002; **19**:309-310.
34 An FS, Zhang Y, Li DQ *et al.* [A novel missense mutation, K124N, in the troponin T gene of Chinese populations with hypertrophic cardiomyopathy]. *Zhonghua yi xue za zhi* 2004; **84**:1340-1343.
35 Gafurov B, Fredricksen S, Cai A, Brenner B, Chase PB, Chalovich JM. The Delta 14 mutation of human cardiac troponin T enhances ATPase activity and alters the cooperative binding of S1-ADP to regulated actin. *Biochemistry* 2004; **43**:15276-15285.

36 Garcia-Castro M, Reguero JR, Moris C *et al.* Prevalence and spectrum of mutations in the sarcomeric troponin T and I genes in a cohort of Spanish cardiac hypertrophy patients. *International journal of cardiology* 2007; **121**:115-116.
37 Xu C, Wei M, Su B *et al.* Ile90Met, a novel mutation in the cardiac troponin T gene for familial hypertrophic cardiomyopathy in a Chinese pedigree. *Genetics research* 2008; **90**:445-450.
38 Peddy SB, Vricella LA, Crosson JE *et al.* Infantile restrictive cardiomyopathy resulting from a mutation in the cardiac troponin T gene. *Pediatrics* 2006; **117**:1830-1833.
39 Gruchala M, Ciecwierz D, Wasag B *et al.* Association of the ScaI atrial natriuretic peptide gene polymorphism with nonfatal myocardial infarction and extent of coronary artery disease. *American heart journal* 2003; **145**:125-131.
40 Rubattu S, Stanzione R, Di Angelantonio E *et al.* Atrial natriuretic peptide gene polymorphisms and risk of ischemic stroke in humans. *Stroke; a journal of cerebral circulation* 2004; **35**:814-818.
41 Hodgson-Zingman DM, Karst ML, Zingman LV *et al.* Atrial natriuretic peptide frameshift mutation in familial atrial fibrillation. *The New England journal of medicine* 2008; **359**:158-165.
42 Zhang S, Mao G, Zhang Y *et al.* Association between human atrial natriuretic peptide Val7Met polymorphism and baseline blood pressure, plasma trough irbesartan concentrations, and the antihypertensive efficacy of irbesartan in rural Chinese patients with essential hypertension. *Clinical therapeutics* 2005; **27**:1774-1784.
43 Rubattu S, Bigatti G, Evangelista A *et al.* Association of atrial natriuretic peptide and type a natriuretic peptide receptor gene polymorphisms with left ventricular mass in human essential hypertension. *Journal of the American College of Cardiology* 2006; **48**:499-505.
44 Xue H, Wang S, Wang H *et al.* Atrial natriuretic peptide gene promoter polymorphism is associated with left ventricular hypertrophy in hypertension. *Clinical science* 2008; **114**:131-137.
45 Landstrom AP, Parvatiyar MS, Pinto JR *et al.* Molecular and functional characterization of novel hypertrophic cardiomyopathy susceptibility mutations in TNNC1-encoded troponin C. *Journal of molecular and cellular cardiology* 2008; **45**:281-288.
46 Chung WK, Kitner C, Maron BJ. Novel frameshift mutation in Troponin C ( TNNC1) associated with hypertrophic cardiomyopathy and sudden death. *Cardiology in the young* 2011; **21**:345-348.
47 Dong WJ, Xing J, Ouyang Y, An J, Cheung HC. Structural kinetics of cardiac troponin C mutants linked to familial hypertrophic and dilated cardiomyopathy in troponin complexes. *The Journal of biological chemistry* 2008; **283**:3424-3432.
48 Pinto JR, Siegfried JD, Parvatiyar MS *et al.* Functional characterization of TNNC1 rare variants identified in dilated cardiomyopathy. *The Journal of biological chemistry* 2011; **286**:34404-34412.
49 Mohapatra B, Jimenez S, Lin JH *et al.* Mutations in the muscle LIM protein and alpha-actinin-2 genes in dilated cardiomyopathy and endocardial fibroelastosis. *Molecular genetics and metabolism* 2003; **80**:207-215.
50 Andersen PS, Havndrup O, Bundgaard H *et al.* Myosin light chain mutations in familial hypertrophic cardiomyopathy: phenotypic presentation and frequency in Danish and South African populations. *Journal of medical genetics* 2001; **38**:E43.
51 Okubo A, Miyoshi O, Baba K *et al.* A novel GATA4 mutation completely segregated with atrial septal defect in a large Japanese family. *Journal of medical genetics* 2004; **41**:e97.
52 Zhang L, Tumer Z, Jacobsen JR, Andersen PS, Tommerup N, Larsen LA. Screening of 99 Danish patients with congenital heart disease for GATA4 mutations. *Genetic testing* 2006; **10**:277-280.
53 Zhang W, Li X, Shen A, Jiao W, Guan X, Li Z. GATA4 mutations in 486 Chinese patients with congenital heart disease. *European journal of medical genetics* 2008; **51**:527-535.
54 Nemer G, Fadlalah F, Usta J *et al.* A novel mutation in the GATA4 gene in patients with

Tetralogy of Fallot. *Human mutation* 2006; **27**:293-294.
55 Schluterman MK, Krysiak AE, Kathiriya IS *et al.* Screening and biochemical analysis of GATA4 sequence variations identified in patients with congenital heart disease. *American journal of medical genetics Part A* 2007; **143A**:817-823.
56 George CH, Higgs GV, Lai FA. Ryanodine receptor mutations associated with stress-induced ventricular tachycardia mediate increased calcium release in stimulated cardiomyocytes. *Circulation research* 2003; **93**:531-540.
57 Tester DJ, Kopplin LJ, Will ML, Ackerman MJ. Spectrum and prevalence of cardiac ryanodine receptor (RyR2) mutations in a cohort of unrelated patients referred explicitly for long QT syndrome genetic testing. *Heart rhythm : the official journal of the Heart Rhythm Society* 2005; **2**:1099-1105.
58 Tester DJ, Dura M, Carturan E *et al.* A mechanism for sudden infant death syndrome (SIDS): stress-induced leak via ryanodine receptors. *Heart rhythm : the official journal of the Heart Rhythm Society* 2007; **4**:733-739.
59 Postma AV, Denjoy I, Kamblock J *et al.* Catecholaminergic polymorphic ventricular tachycardia: RYR2 mutations, bradycardia, and follow up of the patients. *Journal of medical genetics* 2005; **42**:863-870.
60 Bhuiyan ZA, van den Berg MP, van Tintelen JP *et al.* Expanding spectrum of human RYR2-related disease: new electrocardiographic, structural, and genetic features. *Circulation* 2007; **116**:1569-1576.
61 Ransom JF, King IN, Garg V, Srivastava D. A rare human sequence variant reveals myocardin autoinhibition. *The Journal of biological chemistry* 2008; **283**:35845-35852.
62 Chen SN, Czernuszewicz G, Tan Y *et al.* Human molecular genetic and functional studies identify TRIM63, encoding Muscle RING Finger Protein 1, as a novel gene for human hypertrophic cardiomyopathy. *Circulation research* 2012; **111**:907-919.
