# Supplementary material for: Global DNA methylation and transcriptional analyses of human ESC-derived cardiomyocytes
Source: Protein Cell. 2014 Jan 29;5(1):59–68. doi: 10.1007/s13238-013-0016-x (PMC3938846; doi:10.1007/s13238-013-0016-x)
Supplement: Supplementary file 10 — Table S7: GO analysis of hCM differentially down-regulated genes-Molecular Function. This file contains the list of GO terms in the category of molecular function that were significantly (p<0.05) overrepresented among the 401 hCM differentiallydown-regulated genes [file 13238_2013_16_MOESM10_ESM.pdf]

Gene Name	Description and ID	DNA methylation		
		ES-mCpG	NSC-mCpG	CM-mCpG
NANOG	Nanog homeobox [Source:HGNC Symbol;Acc:20857]	0.04	0.06	0.63
SERPINB1	serpin peptidase inhibitor, clade B (ovalbumin), member 1 [Source:HGNC Symbol;Acc:3311]	0.16	0.13	0.64
CBR1	carbonyl reductase 1 [Source:HGNC Symbol;Acc:1548]	0.02	0.15	0.53
PM20D2	peptidase M20 domain containing 2 [Source:HGNC Symbol;Acc:21408]	0.19	0.29	0.66
SKAP1	src kinase associated phosphoprotein 1 [Source:HGNC Symbol;Acc:15605]	0.32	0.26	0.68
ABCB4	ATP-binding cassette, sub-family B (MDR/TAP), member 4 [Source:HGNC Symbol;Acc:45]	0.08	0.02	0.43
TUSC3	tumor suppressor candidate 3 [Source:HGNC Symbol;Acc:30242]	0.52	0.38	0.87
HORMAD2	HORMA domain containing 2 [Source:HGNC Symbol;Acc:28383]	0.47	0.39	0.81
LOXL3	lysyl oxidase-like 3 [Source:HGNC Symbol;Acc:13869]	0.18	0.17	0.51
UAP1	UDP-N-acteylglucosamine pyrophosphorylase 1 [Source:HGNC Symbol;Acc:12457]	0.53	0.26	0.84
CPLX2	complexin 2 [Source:HGNC Symbol;Acc:2310]	0.39	0.23	0.69
CCDC81	coiled-coil domain containing 81 [Source:HGNC Symbol;Acc:26281]	0.3	0.16	0.59
CREB5	cAMP responsive element binding protein 5 [Source:HGNC Symbol;Acc:16844]	0.13	0.07	0.4
GPR113	G protein-coupled receptor 113 [Source:HGNC Symbol;Acc:18989]	0.37	0.41	0.67
AMN	amnion associated transmembrane protein [Source:HGNC Symbol;Acc:14604]	0.03	0.03	0.29
PRELID2	PRELI domain containing 2 [Source:HGNC Symbol;Acc:28306]	0.01	0.02	0.28
PTPLAD2	protein tyrosine phosphatase-like A domain containing 2 [Source:HGNC Symbol;Acc:20920]	0.22	0.43	0.68
HSBP1L1	heat shock factor binding protein 1-like 1 [Source:HGNC Symbol;Acc:37243]	0.57	0.53	0.82
ATP2B2	ATPase, Ca++ transporting, plasma membrane 2 [Source:HGNC Symbol;Acc:815]	0.37	0.33	0.62
PRKAR2B	protein kinase, cAMP-dependent, regulatory, type II, beta [Source:HGNC Symbol;Acc:9392]	0.4	0.52	0.77
GDF15	growth differentiation factor 15 [Source:HGNC Symbol;Acc:30142]	0.35	0.21	0.6
A4GNT	alpha-1,4-N-acetylglucosaminyltransferase [Source:HGNC Symbol;Acc:17968]	0.67	0.5	0.92
MFSD2B	major facilitator superfamily domain containing 2B [Source:HGNC Symbol;Acc:37207]	0.27	0.5	0.75
CHD7	chromodomain helicase DNA binding protein 7 [Source:HGNC Symbol;Acc:20626]	0.16	0.13	0.41
LDHD	lactate dehydrogenase D [Source:HGNC Symbol;Acc:19708]	0.39	0.43	0.67
PTPDC1	protein tyrosine phosphatase domain containing 1 [Source:HGNC Symbol;Acc:30184]	0.4	0.39	0.64
TIFA	TRAF-interacting protein with forkhead-associated domain [Source:HGNC Symbol;Acc:19075]	0.27	0.21	0.51
FOXD2	forkhead box D2 [Source:HGNC Symbol;Acc:3803]	0.26	0.29	0.52
PASK	PAS domain containing serine/threonine kinase [Source:HGNC Symbol;Acc:17270]	0.44	0.4	0.67
HOXB2	homeobox B2 [Source:HGNC Symbol;Acc:5113]	0.13	0.15	0.38
RBM43	RNA binding motif protein 43 [Source:HGNC Symbol;Acc:24790]	0.2	0.24	0.47
PDCL3	phosducin-like 3 [Source:HGNC Symbol;Acc:28860]	0.47	0.29	0.7
SERHL2	serine hydrolase-like 2 [Source:HGNC Symbol;Acc:29446]	0.02	0.12	0.34
SERPINA11	serpin peptidase inhibitor, clade A (alpha-1 antiproteinase, antitrypsin), member 11 [Source:HGNC Symbol;Acc:19193]	0.09	0.15	0.37
ARHGAP9	Rho GTPase activating protein 9 [Source:HGNC Symbol;Acc:14130]	0.78	0.76	1
OR10A5	olfactory receptor, family 10, subfamily A, member 5 [Source:HGNC Symbol;Acc:15131]	0.72	0.59	0.94
PRAF2	PRA1 domain family, member 2 [Source:HGNC Symbol;Acc:28911]	0.44	0.46	0.67
PLCL2	phospholipase C-like 2 [Source:HGNC Symbol;Acc:9064]	0.15	0.25	0.46
MAT1A	methionine adenosyltransferase I, alpha [Source:HGNC Symbol;Acc:6903]	0.27	0.41	0.62
HLA-H	major histocompatibility complex, class I, H (pseudogene) [Source:HGNC Symbol;Acc:4965]	0.09	0.17	0.38
C16orf54	chromosome 16 open reading frame 54 [Source:HGNC Symbol;Acc:26649]	0.64	0.65	0.86
OBP2B	odorant binding protein 2B [Source:HGNC Symbol;Acc:23381]	0.62	0.64	0.85
ZNF239	zinc finger protein 239 [Source:HGNC Symbol;Acc:13031]	0.6	0.69	0.89
HCK	hemopoietic cell kinase [Source:HGNC Symbol;Acc:4840]	0.23	0.35	0.55
SPATA13	spermatogenesis associated 13 [Source:HGNC Symbol;Acc:23222]	0.13	0.07	0.33
GRASP	GRP1 (general receptor for phosphoinositides 1)-associated scaffold protein [Source:HGNC Symbol;Acc:18707]	0.41	0.29	0.61
C1QTNF1	C1q and tumor necrosis factor related protein 1 [Source:HGNC Symbol;Acc:14324]	0.33	0.12	0.53
SLIT1	slit homolog 1 (Drosophila) [Source:HGNC Symbol;Acc:11085]	0.17	0.17	0.37
DGAT2	diacylglycerol O-acyltransferase 2 [Source:HGNC Symbol;Acc:16940]	0.11	0.15	0.35
NT5DC1	5'-nucleotidase domain containing 1 [Source:HGNC Symbol;Acc:21556]	0.45	0.47	0.66
CYS1	cystin 1 [Source:HGNC Symbol;Acc:18525]	0.37	0.35	0.56
FEZ1	fasciculation and elongation protein zeta 1 (zygin I) [Source:HGNC Symbol;Acc:3659]	0.51	0.58	0.77
BACH2	BTB and CNC homology 1, basic leucine zipper transcription factor 2 [Source:HGNC Symbol;Acc:14078]	0.51	0.58	0.77
LRRTM3	leucine rich repeat transmembrane neuronal 3 [Source:HGNC Symbol;Acc:19410]	0.59	0.74	0.93

MFSD6L	major facilitator superfamily domain containing 6-like [Source:HGNC Symbol;Acc:26656]	0.42	0.58	0.77
LRRC4	leucine rich repeat containing 4 [Source:HGNC Symbol;Acc:15586]	0.21	0.21	0.4
SLC26A4	solute carrier family 26, member 4 [Source:HGNC Symbol;Acc:8818]	0.42	0.41	0.61
SSFA2	sperm specific antigen 2 [Source:HGNC Symbol;Acc:11319]	0.3	0.26	0.49
ESRRG	estrogen-related receptor gamma [Source:HGNC Symbol;Acc:3474]	0.01	0.1	0.29
CDKN1B	cyclin-dependent kinase inhibitor 1B (p27, Kip1) [Source:HGNC Symbol;Acc:1785]	0.16	0.06	0.35
STXBP5L	syntaxin binding protein 5-like [Source:HGNC Symbol;Acc:30757]	0.11	0.23	0.42
KCNJ12	potassium inwardly-rectifying channel, subfamily J, member 12 [Source:HGNC Symbol;Acc:6258]	0.64	0.67	0.86
TCIRG1	T-cell, immune regulator 1, ATPase, H+ transporting, lysosomal V0 subunit A3 [Source:HGNC Symbol;Acc:11647]	0.78	0.68	0.97
CXCL5	chemokine (C-X-C motif) ligand 5 [Source:HGNC Symbol;Acc:10642]	0.44	0.55	0.74
ST3GAL4	ST3 beta-galactoside alpha-2,3-sialyltransferase 4 [Source:HGNC Symbol;Acc:10864]	0.73	0.71	0.91
NOXO1	NADPH oxidase organizer 1 [Source:HGNC Symbol;Acc:19404]	0.72	0.67	0.9
SLC46A3	solute carrier family 46, member 3 [Source:HGNC Symbol;Acc:27501]	0.65	0.72	0.9
SYN2	synapsin II [Source:HGNC Symbol;Acc:11495]	0.36	0.47	0.65
MYH2	myosin, heavy chain 2, skeletal muscle, adult [Source:HGNC Symbol;Acc:7572]	0.47	0.32	0.65
TMEM171	transmembrane protein 171 [Source:HGNC Symbol;Acc:27031]	0.4	0.63	0.81
PITX2	paired-like homeodomain 2 [Source:HGNC Symbol;Acc:9005]	0.13	0.15	0.33
CLDN9	claudin 9 [Source:HGNC Symbol;Acc:2051]	0.02	0.21	0.39
GOLGA1	golgin A1 [Source:HGNC Symbol;Acc:4424]	0.12	0.13	0.31
HPS1	Hermansky-Pudlak syndrome 1 [Source:HGNC Symbol;Acc:5163]	0.42	0.39	0.6
NEURL	neuralized homolog (Drosophila) [Source:HGNC Symbol;Acc:7761]	0.37	0.41	0.59
TP53BP1	tumor protein p53 binding protein 1 [Source:HGNC Symbol;Acc:11999]	0.09	0.13	0.31
DSP	desmoplakin [Source:HGNC Symbol;Acc:3052]	0.14	0.2	0.38
KCTD17	potassium channel tetramerisation domain containing 17 [Source:HGNC Symbol;Acc:25705]	0.24	0.18	0.42
TM4SF18	transmembrane 4 L six family member 18 [Source:HGNC Symbol;Acc:25181]	0.1	0.24	0.42
ZMAT1	zinc finger, matrin-type 1 [Source:HGNC Symbol;Acc:29377]	0.54	0.54	0.72
RBP4	retinol binding protein 4, plasma [Source:HGNC Symbol;Acc:9922]	0.44	0.51	0.69
HLA-DPA1	major histocompatibility complex, class II, DP alpha 1 [Source:HGNC Symbol;Acc:4938]	0.69	0.7	0.87
AQP9	aquaporin 9 [Source:HGNC Symbol;Acc:643]	0.61	0.55	0.78
RUSC1	RUN and SH3 domain containing 1 [Source:HGNC Symbol;Acc:17153]	0.44	0.51	0.68
LYPD6	LY6/PLAUR domain containing 6 [Source:HGNC Symbol;Acc:28751]	0.34	0.48	0.65
ACPL2	acid phosphatase-like 2 [Source:HGNC Symbol;Acc:26303]	0.35	0.61	0.78
ATP8A1	ATPase, aminophospholipid transporter (APLT), class I, type 8A, member 1 [Source:HGNC Symbol;Acc:13531]	0	0	0.17
CNKSR2	connector enhancer of kinase suppressor of Ras 2 [Source:HGNC Symbol;Acc:19701]	0.15	0.11	0.32
NUDT19	nudix (nucleoside diphosphate linked moiety X)-type motif 19 [Source:HGNC Symbol;Acc:32036]	0.07	0.15	0.32
TMEM30B	transmembrane protein 30B [Source:HGNC Symbol;Acc:27254]	0.04	0.15	0.32
DDX60L	DEAD (Asp-Glu-Ala-Asp) box polypeptide 60-like [Source:HGNC Symbol;Acc:26429]	0.37	0.45	0.62
CACNG4	calcium channel, voltage-dependent, gamma subunit 4 [Source:HGNC Symbol;Acc:1408]	0.13	0.24	0.41
SHISA5	shisa homolog 5 (Xenopus laevis) [Source:HGNC Symbol;Acc:30376]	0.76	0.77	0.94
HLA-DRA	major histocompatibility complex, class II, DR alpha [Source:HGNC Symbol;Acc:4947]	0.47	0.52	0.69
PPP3CC	protein phosphatase 3, catalytic subunit, gamma isozyme [Source:HGNC Symbol;Acc:9316]	0.56	0.67	0.84
ACVR1C	activin A receptor, type IC [Source:HGNC Symbol;Acc:18123]	0.4	0.4	0.56
TNNI3	troponin I type 3 (cardiac) [Source:HGNC Symbol;Acc:11947]	0.72	0.71	0.88
NCKAP1L	NCK-associated protein 1-like [Source:HGNC Symbol;Acc:4862]	0.75	0.74	0.91
TNFRSF8	tumor necrosis factor receptor superfamily, member 8 [Source:HGNC Symbol;Acc:11923]	0.1	0.12	0.28
GABRA5	gamma-aminobutyric acid (GABA) A receptor, alpha 5 [Source:HGNC Symbol;Acc:4079]	0.59	0.63	0.79
VPREB3	pre-B lymphocyte 3 [Source:HGNC Symbol;Acc:12710]	0.69	0.73	0.89
MLKL	mixed lineage kinase domain-like [Source:HGNC Symbol;Acc:26617]	0.03	0.11	0.27
GCC2	GRIP and coiled-coil domain containing 2 [Source:HGNC Symbol;Acc:23218]	0.49	0.41	0.65
C9orf172	chromosome 9 open reading frame 172 [Source:HGNC Symbol;Acc:37284]	0.7	0.61	0.86
PKDREJ	polycystic kidney disease (polycystin) and REJ homolog (sperm receptor for egg jelly homolog, sea urchin) [Source:HGNC Symbol;Acc:9015]	0.38	0.52	0.68
SPINT3	serine peptidase inhibitor, Kunitz type, 3 [Source:HGNC Symbol;Acc:11248]	0.72	0.52	0.88
INHBE	inhibin, beta E [Source:HGNC Symbol;Acc:24029]	0.28	0.51	0.67
NMI	N-myc (and STAT) interactor [Source:HGNC Symbol;Acc:7854]	0.04	0.38	0.54
AKAP2	A kinase (PRKA) anchor protein 2 [Source:HGNC Symbol;Acc:372]	0.03	0.04	0.2
SYT7	synaptotagmin VII [Source:HGNC Symbol;Acc:11514]	0.22	0.13	0.38
TACC2	transforming, acidic coiled-coil containing protein 2 [Source:HGNC Symbol;Acc:11523]	0.19	0.14	0.35
FCHO2	FCH domain only 2 [Source:HGNC Symbol;Acc:25180]	0.37	0.42	0.58

PCDHB16	protocadherin beta 16 [Source:HGNC Symbol;Acc:14546]	0	0.05	0.21
PRICKLE2	prickle homolog 2 (Drosophila) [Source:HGNC Symbol;Acc:20340]	0.32	0.25	0.48
RNASEL	ribonuclease L (2',5'-oligoisoadenylate synthetase-dependent) [Source:HGNC Symbol;Acc:10050]	0.43	0.31	0.59
GPBAR1	G protein-coupled bile acid receptor 1 [Source:HGNC Symbol;Acc:19680]	0.81	0.66	0.97
CTGF	connective tissue growth factor [Source:HGNC Symbol;Acc:2500]	0.18	0.17	0.33
KCNH3	potassium voltage-gated channel, subfamily H (eag-related), member 3 [Source:HGNC Symbol;Acc:6252]	0.3	0.29	0.45
BEGAIN	brain-enriched guanylate kinase-associated [Source:HGNC Symbol;Acc:24163]	0.6	0.61	0.76
PYCARD	PYD and CARD domain containing [Source:HGNC Symbol;Acc:16608]	0.62	0.63	0.78
OTUB2	OTU domain, ubiquitin aldehyde binding 2 [Source:HGNC Symbol;Acc:20351]	0.64	0.62	0.79
LSMD1	LSM domain containing 1 [Source:HGNC Symbol;Acc:28212]	0.05	0.08	0.23
IGLL1	immunoglobulin lambda-like polypeptide 1 [Source:HGNC Symbol;Acc:5870]	0.72	0.68	0.87
SOX15	SRY (sex determining region Y)-box 15 [Source:HGNC Symbol;Acc:11196]	0.57	0.61	0.76
GJB4	gap junction protein, beta 4, 30.3kDa [Source:HGNC Symbol;Acc:4286]	0.74	0.67	0.89
OR4C11	olfactory receptor, family 4, subfamily C, member 11 [Source:HGNC Symbol;Acc:15167]	0.85	0.67	1
GPR85	G protein-coupled receptor 85 [Source:HGNC Symbol;Acc:4536]	0.09	0.09	0.24
TBX5	T-box 5 [Source:HGNC Symbol;Acc:11604]	0.03	0.02	0.18
DLEU7	deleted in lymphocytic leukemia, 7 [Source:HGNC Symbol;Acc:17567]	0.06	0.04	0.21
HOXB3	homeobox B3 [Source:HGNC Symbol;Acc:5114]	0.06	0.03	0.21
PEX7	peroxisomal biogenesis factor 7 [Source:HGNC Symbol;Acc:8860]	0.3	0.34	0.49
GUCA2B	guanylate cyclase activator 2B (uroguanylin) [Source:HGNC Symbol;Acc:4683]	0.79	0.76	0.94
PDE1B	phosphodiesterase 1B, calmodulin-dependent [Source:HGNC Symbol;Acc:8775]	0.5	0.55	0.7
ZNF674	zinc finger protein 674 [Source:HGNC Symbol;Acc:17625]	0.55	0.49	0.7
CNPY1	canopy 1 homolog (zebrafish) [Source:HGNC Symbol;Acc:27786]	0.42	0.36	0.56
NES	nestin [Source:HGNC Symbol;Acc:7756]	0.02	0.01	0.16
ZNF700	zinc finger protein 700 [Source:HGNC Symbol;Acc:25292]	0.08	0.07	0.22
SLC3A1	solute carrier family 3 (cystine, dibasic and neutral amino acid transporters, activator of cystine, dibasic and neutral amino acid transport), member 1 [Source:HGNC Symbol;Acc:11025]	0.86	0.85	1
SLC7A10	solute carrier family 7 (neutral amino acid transporter light chain, asc system), member 10 [Source:HGNC Symbol;Acc:11058]	0.7	0.68	0.84
LEMD3	LEM domain containing 3 [Source:HGNC Symbol;Acc:28887]	0.66	0.64	0.8
IRF2	interferon regulatory factor 2 [Source:HGNC Symbol;Acc:6117]	0.08	0.05	0.22
C14orf93	chromosome 14 open reading frame 93 [Source:HGNC Symbol;Acc:20162]	0.1	0.13	0.27
CABP7	calcium binding protein 7 [Source:HGNC Symbol;Acc:20834]	0.64	0.61	0.78
CHKA	choline kinase alpha [Source:HGNC Symbol;Acc:1937]	0.36	0.39	0.53
COLQ	collagen-like tail subunit (single strand of homotrimer) of asymmetric acetylcholinesterase [Source:HGNC Symbol;Acc:2226]	0.86	0.82	1
APOA2	apolipoprotein A-II [Source:HGNC Symbol;Acc:601]	0.82	0.86	1
DLEC1	deleted in lung and esophageal cancer 1 [Source:HGNC Symbol;Acc:2899]	0.48	0.52	0.66
SLC17A7	solute carrier family 17 (sodium-dependent inorganic phosphate cotransporter), member 7 [Source:HGNC Symbol;Acc:16704]	0.68	0.73	0.87
ADAM8	ADAM metallopeptidase domain 8 [Source:HGNC Symbol;Acc:215]	0.66	0.6	0.8
IQCF2	IQ motif containing F2 [Source:HGNC Symbol;Acc:31815]	0.86	0.79	1
TCF7L1	transcription factor 7-like 1 (T-cell specific, HMG-box) [Source:HGNC Symbol;Acc:11640]	0.58	0.66	0.8
PRRT1	proline-rich transmembrane protein 1 [Source:HGNC Symbol;Acc:13943]	0.08	0.19	0.33
EMP3	epithelial membrane protein 3 [Source:HGNC Symbol;Acc:3335]	0.29	0.1	0.43
AHNAK2	AHNAK nucleoprotein 2 [Source:HGNC Symbol;Acc:20125]	0.19	0.38	0.52
RBM24	RNA binding motif protein 24 [Source:HGNC Symbol;Acc:21539]	0.66	0.03	0.8
APCDD1	adenomatosis polyposis coli down-regulated 1 [Source:HGNC Symbol;Acc:15718]	0.21	0.19	0.35
TNFSF11	tumor necrosis factor (ligand) superfamily, member 11 [Source:HGNC Symbol;Acc:11926]	0.13	0.17	0.31
ACADL	acyl-CoA dehydrogenase, long chain [Source:HGNC Symbol;Acc:88]	0.14	0.22	0.36
RAB21	RAB21, member RAS oncogene family [Source:HGNC Symbol;Acc:18263]	0.17	0.04	0.31
RIPK3	receptor-interacting serine-threonine kinase 3 [Source:HGNC Symbol;Acc:10021]	0.33	0.34	0.48
C11orf70	chromosome 11 open reading frame 70 [Source:HGNC Symbol;Acc:28188]	0.22	0.28	0.42
TAPBP	TAP binding protein (tapasin) [Source:HGNC Symbol;Acc:11566]	0.17	0.28	0.42
TRIM21	tripartite motif containing 21 [Source:HGNC Symbol;Acc:11312]	0.78	0.78	0.91
INPP4A	inositol polyphosphate-4-phosphatase, type I, 107kDa [Source:HGNC Symbol;Acc:6074]	0.06	0.06	0.19
POLE4	polymerase (DNA-directed), epsilon 4, accessory subunit [Source:HGNC Symbol;Acc:18755]	0.04	0.04	0.17
ARID3C	AT rich interactive domain 3C (BRIGHT-like) [Source:HGNC Symbol;Acc:21209]	0.71	0.71	0.84
CNTN2	contactin 2 (axonal) [Source:HGNC Symbol;Acc:2172]	0.61	0.62	0.75
TICAM2	toll-like receptor adaptor molecule 2 [Source:HGNC Symbol;Acc:21354]	0.08	0.06	0.21
TICAM1	toll-like receptor adaptor molecule 1 [Source:HGNC Symbol;Acc:18348]	0.04	0.06	0.19
LRRC32	leucine rich repeat containing 32 [Source:HGNC Symbol;Acc:4161]	0.2	0.17	0.33
DNLZ	DNL-type zinc finger [Source:HGNC Symbol;Acc:33879]	0.32	0.36	0.49
FRMPD4	FERM and PDZ domain containing 4 [Source:HGNC Symbol;Acc:29007]	0.36	0.4	0.53
THPO	thrombopoietin [Source:HGNC Symbol;Acc:11795]	0.75	0.7	0.88
EFNA2	ephrin-A2 [Source:HGNC Symbol;Acc:3222]	0.14	0.2	0.33

ADCY1	adenylate cyclase 1 (brain) [Source:HGNC Symbol;Acc:232]	0.62	0.68	0.81
FMOD	fibromodulin [Source:HGNC Symbol;Acc:3774]	0.69	0.62	0.82
OR5V1	olfactory receptor, family 5, subfamily V, member 1 [Source:HGNC Symbol;Acc:13972]	0.78	0.71	0.91
FAIM2	Fas apoptotic inhibitory molecule 2 [Source:HGNC Symbol;Acc:17067]	0.74	0.65	0.87
SNRNP48	small nuclear ribonucleoprotein 48kDa (U11/U12) [Source:HGNC Symbol;Acc:21368]	0.36	0.27	0.49
ADAMTS8	ADAM metallopeptidase with thrombospondin type 1 motif, 8 [Source:HGNC Symbol;Acc:224]	0.68	0.59	0.81
ANP32B	acidic (leucine-rich) nuclear phosphoprotein 32 family, member B [Source:HGNC Symbol;Acc:16677]	0.08	0.18	0.31
ABCC3	ATP-binding cassette, sub-family C (CFTR/MRP), member 3 [Source:HGNC Symbol;Acc:54]	0.27	0.17	0.4
C15orf56	chromosome 15 open reading frame 56 [Source:HGNC Symbol;Acc:33868]	0.53	0.65	0.78
ADO	2-aminoethanethiol (cysteamine) dioxygenase [Source:HGNC Symbol;Acc:23506]	0.71	0.56	0.84
ADAMTS2	ADAM metallopeptidase with thrombospondin type 1 motif, 2 [Source:HGNC Symbol;Acc:218]	0.31	0.48	0.61
CD72	CD72 molecule [Source:HGNC Symbol;Acc:1696]	0.87	0.69	1
CTDSPL	CTD (carboxy-terminal domain, RNA polymerase II, polypeptide A) small phosphatase-like [Source:HGNC Symbol;Acc:16890]	0.52	0.29	0.65
TNFSF10	tumor necrosis factor (ligand) superfamily, member 10 [Source:HGNC Symbol;Acc:11925]	0.54	0.28	0.67
ABCF2	ATP-binding cassette, sub-family F (GCN20), member 2 [Source:HGNC Symbol;Acc:71]	0.28	0.28	0.41
C11orf93	chromosome 11 open reading frame 93 [Source:HGNC Symbol;Acc:26978]	0.19	0.34	0.47
HOXB1	homeobox B1 [Source:HGNC Symbol;Acc:5111]	0.19	0.34	0.47
PAX5	paired box 5 [Source:HGNC Symbol;Acc:8619]	0.56	0.53	0.69
RASSF1	Ras association (RalGDS/AF-6) domain family member 1 [Source:HGNC Symbol;Acc:9882]	0.17	0.42	0.54
STC1	stanniocalcin 1 [Source:HGNC Symbol;Acc:11373]	0.02	0.02	0.14
PPP1R16B	protein phosphatase 1, regulatory subunit 16B [Source:HGNC Symbol;Acc:15850]	0.01	0.02	0.14
PATZ1	POZ (BTB) and AT hook containing zinc finger 1 [Source:HGNC Symbol;Acc:13071]	0.04	0.05	0.17
ACP2	acid phosphatase 2, lysosomal [Source:HGNC Symbol;Acc:123]	0.55	0.55	0.67
GLIPR1L2	GLI pathogenesis-related 1 like 2 [Source:HGNC Symbol;Acc:28592]	0.6	0.6	0.72
SULT1C2	sulfotransferase family, cytosolic, 1C, member 2 [Source:HGNC Symbol;Acc:11456]	0.19	0.18	0.31
ESYT3	extended synaptotagmin-like protein 3 [Source:HGNC Symbol;Acc:24295]	0.6	0.59	0.72
SLC36A2	solute carrier family 36 (proton/amino acid symporter), member 2 [Source:HGNC Symbol;Acc:18762]	0.78	0.77	0.9
NRIP2	nuclear receptor interacting protein 2 [Source:HGNC Symbol;Acc:23078]	0.49	0.5	0.62
IL6	interleukin 6 (interferon, beta 2) [Source:HGNC Symbol;Acc:6018]	0.59	0.6	0.72
ATRX	alpha thalassemia/mental retardation syndrome X-linked [Source:HGNC Symbol;Acc:886]	0.35	0.33	0.47
CYB5D1	cytochrome b5 domain containing 1 [Source:HGNC Symbol;Acc:26516]	0.04	0.06	0.18
BTBD18	BTB (POZ) domain containing 18 [Source:HGNC Symbol;Acc:37214]	0.54	0.52	0.66
RCN3	reticulocalbin 3, EF-hand calcium binding domain [Source:HGNC Symbol;Acc:21145]	0.74	0.76	0.88
MYT1L	myelin transcription factor 1-like [Source:HGNC Symbol;Acc:7623]	0.6	0.62	0.74
PACS2	phosphofurin acidic cluster sorting protein 2 [Source:HGNC Symbol;Acc:23794]	0.36	0.33	0.48
FYCO1	FYVE and coiled-coil domain containing 1 [Source:HGNC Symbol;Acc:14673]	0.09	0.06	0.21
FAM155B	family with sequence similarity 155, member B [Source:HGNC Symbol;Acc:30701]	0.53	0.5	0.65
ENTPD2	ectonucleoside triphosphate diphosphohydrolase 2 [Source:HGNC Symbol;Acc:3364]	0.55	0.59	0.71
ATPBD4	ATP binding domain 4 [Source:HGNC Symbol;Acc:30543]	0.06	0.02	0.18
STARD8	StAR-related lipid transfer (START) domain containing 8 [Source:HGNC Symbol;Acc:19161]	0.6	0.55	0.72
MPZL2	myelin protein zero-like 2 [Source:HGNC Symbol;Acc:3496]	0.5	0.45	0.62
BCL6	B-cell CLL/lymphoma 6 [Source:HGNC Symbol;Acc:1001]	0.74	0.79	0.91
DFNA5	deafness, autosomal dominant 5 [Source:HGNC Symbol;Acc:2810]	0.27	0.33	0.45
RAET1E	retinoic acid early transcript 1E [Source:HGNC Symbol;Acc:16793]	0.88	0.82	1
SPAG17	sperm associated antigen 17 [Source:HGNC Symbol;Acc:26620]	0.61	0.54	0.73
C1orf116	chromosome 1 open reading frame 116 [Source:HGNC Symbol;Acc:28667]	0.77	0.7	0.89
PAK3	p21 protein (Cdc42/Rac)-activated kinase 3 [Source:HGNC Symbol;Acc:8592]	0.25	0.33	0.45
LRRTM1	leucine rich repeat transmembrane neuronal 1 [Source:HGNC Symbol;Acc:19408]	0.2	0.29	0.41
FNBP1	formin binding protein 1 [Source:HGNC Symbol;Acc:17069]	0.23	0.32	0.44
KCNA4	potassium voltage-gated channel, shaker-related subfamily, member 4 [Source:HGNC Symbol;Acc:6222]	0.78	0.65	0.9
LPO	lactoperoxidase [Source:HGNC Symbol;Acc:6678]	0.83	0.69	0.95
RAG1	recombination activating gene 1 [Source:HGNC Symbol;Acc:9831]	0.82	0.67	0.94
ZNF211	zinc finger protein 211 [Source:HGNC Symbol;Acc:13003]	0.62	0.46	0.74

CD79A	CD79a molecule, immunoglobulin-associated alpha [Source:HGNC Symbol;Acc:1698]	0.74	0.56	0.86
PXK	PX domain containing serine/threonine kinase [Source:HGNC Symbol;Acc:23326]	0.88	0.7	1
ARHGAP32	Rho GTPase activating protein 32 [Source:HGNC Symbol;Acc:17399]	0.84	0.65	0.96
PALM3	paralemmin 3 [Source:HGNC Symbol;Acc:33274]	0.48	0.29	0.6
REM2	RAS (RAD and GEM)-like GTP binding 2 [Source:HGNC Symbol;Acc:20248]	0.59	0.39	0.71
ANTXR1	anthrax toxin receptor 1 [Source:HGNC Symbol;Acc:21014]	0.54	0.34	0.66
VARS2	valyl-tRNA synthetase 2, mitochondrial [Source:HGNC Symbol;Acc:21642]	0.32	0.52	0.64
BCAS4	breast carcinoma amplified sequence 4 [Source:HGNC Symbol;Acc:14367]	0.18	0.61	0.73
CCL8	chemokine (C-C motif) ligand 8 [Source:HGNC Symbol;Acc:10635]	0.69	0.49	0.8
MRGPRF	MAS-related GPR, member F [Source:HGNC Symbol;Acc:24828]	0.82	0.53	0.93
TFAP2E	transcription factor AP-2 epsilon (activating enhancer binding protein 2 epsilon) [Source:HGNC Symbol;Acc:30774]	0.34	0.35	0.46
NOSIP	nitric oxide synthase interacting protein [Source:HGNC Symbol;Acc:17946]	0.35	0.41	0.52
PRRG2	proline rich Gla (G-carboxyglutamic acid) 2 [Source:HGNC Symbol;Acc:9470]	0.35	0.41	0.52
DNPEP	aspartyl aminopeptidase [Source:HGNC Symbol;Acc:2981]	0.45	0.38	0.56
MAP6D1	MAP6 domain containing 1 [Source:HGNC Symbol;Acc:25753]	0.16	0.12	0.27
SHFM1	split hand/foot malformation (ectrodactyly) type 1 [Source:HGNC Symbol;Acc:10845]	0.01	0.01	0.12
HEBP2	heme binding protein 2 [Source:HGNC Symbol;Acc:15716]	0.05	0.04	0.16
IL20	interleukin 20 [Source:HGNC Symbol;Acc:6002]	0.53	0.53	0.64
KRTAP4-7	keratin associated protein 4-7 [Source:HGNC Symbol;Acc:18898]	0.84	0.84	0.95
ACAT2	acetyl-CoA acetyltransferase 2 [Source:HGNC Symbol;Acc:94]	0.47	0.46	0.58
TC2N	tandem C2 domains, nuclear [Source:HGNC Symbol;Acc:19859]	0.37	0.36	0.48
ZNF502	zinc finger protein 502 [Source:HGNC Symbol;Acc:23718]	0.53	0.55	0.66
FCRL2	Fc receptor-like 2 [Source:HGNC Symbol;Acc:14875]	0.86	0.83	0.97
ZACN	zinc activated ligand-gated ion channel [Source:HGNC Symbol;Acc:29504]	0.79	0.76	0.9
HEXB	hexosaminidase B (beta polypeptide) [Source:HGNC Symbol;Acc:4879]	0.53	0.49	0.64
OMD	osteomodulin [Source:HGNC Symbol;Acc:8134]	0.67	0.63	0.78
MT1H	metallothionein 1H [Source:HGNC Symbol;Acc:7400]	0.14	0.19	0.3
MAGEE1	melanoma antigen family E, 1 [Source:HGNC Symbol;Acc:24934]	0.34	0.39	0.5
FAM174B	family with sequence similarity 174, member B [Source:HGNC Symbol;Acc:34339]	0.72	0.66	0.83
SLC11A1	solute carrier family 11 (proton-coupled divalent metal ion transporters), member 1 [Source:HGNC Symbol;Acc:10907]	0.84	0.77	0.95
TRIOBP	TRIO and F-actin binding protein [Source:HGNC Symbol;Acc:17009]	0.18	0.11	0.29
TBC1D23	TBC1 domain family, member 23 [Source:HGNC Symbol;Acc:25622]	0.76	0.84	0.95
STX1B	syntaxin 1B [Source:HGNC Symbol;Acc:18539]	0.5	0.42	0.61
TP53INP1	tumor protein p53 inducible nuclear protein 1 [Source:HGNC Symbol;Acc:18022]	0.28	0.2	0.39
GGT6	gamma-glutamyltransferase 6 [Source:HGNC Symbol;Acc:26891]	0.6	0.68	0.79
PFKP	phosphofructokinase, platelet [Source:HGNC Symbol;Acc:8878]	0.38	0.28	0.49
TSLP	thymic stromal lymphopoietin [Source:HGNC Symbol;Acc:30743]	0.83	0.72	0.94
TMEM158	transmembrane protein 158 (gene/pseudogene) [Source:HGNC Symbol;Acc:30293]	0.49	0.6	0.71
SNRK	SNF related kinase [Source:HGNC Symbol;Acc:30598]	0.38	0.5	0.61
C9orf9	chromosome 9 open reading frame 9 [Source:HGNC Symbol;Acc:1367]	0.48	0.6	0.71
LPGAT1	lysophosphatidylglycerol acyltransferase 1 [Source:HGNC Symbol;Acc:28985]	0.13	0	0.24
C19orf26	chromosome 19 open reading frame 26 [Source:HGNC Symbol;Acc:28617]	0.56	0.42	0.67
MFSD4	major facilitator superfamily domain containing 4 [Source:HGNC Symbol;Acc:25433]	0.79	0.57	0.9
CDX4	caudal type homeobox 4 [Source:HGNC Symbol;Acc:1808]	0.43	0.66	0.77
PCBP4	poly(rC) binding protein 4 [Source:HGNC Symbol;Acc:8652]	0.31	0.06	0.42
NPB	neuropeptide B [Source:HGNC Symbol;Acc:30099]	0.22	0.49	0.6
AKR7A3	aldo-keto reductase family 7, member A3 (aflatoxin aldehyde reductase) [Source:HGNC Symbol;Acc:390]	0.19	0.55	0.66
KRTAP19-8	keratin associated protein 19-8 [Source:HGNC Symbol;Acc:33898]	0.83	0.38	0.94
NHSL1	NHS-like 1 [Source:HGNC Symbol;Acc:21021]	0.79	0.27	0.9
TNNI2	troponin I type 2 (skeletal, fast) [Source:HGNC Symbol;Acc:11946]	0.83	0.81	0.93
HRC	histidine rich calcium binding protein [Source:HGNC Symbol;Acc:5178]	0.8	0.83	0.93
NPY5R	neuropeptide Y receptor Y5 [Source:HGNC Symbol;Acc:7958]	0.46	0.58	0.68
PTGR1	prostaglandin reductase 1 [Source:HGNC Symbol;Acc:18429]	0.42	0.41	0.52
MOSPD1	motile sperm domain containing 1 [Source:HGNC Symbol;Acc:25235]	0.22	0.24	0.34
CRB2	crumbs homolog 2 (Drosophila) [Source:HGNC Symbol;Acc:18688]	0.45	0.14	0.55
ANGPT1	angiopoietin 1 [Source:HGNC Symbol;Acc:484]	0.07	0.05	0.17
PARP4	poly (ADP-ribose) polymerase family, member 4 [Source:HGNC Symbol;Acc:271]	0.21	0.18	0.31
SEMA3F	sema domain, immunoglobulin domain (Ig), short basic domain, secreted, (semaphorin) 3F [Source:HGNC Symbol;Acc:10728]	0.09	0.12	0.22
GLUD2	glutamate dehydrogenase 2 [Source:HGNC Symbol;Acc:4336]	0.17	0.12	0.27
FAM160B1	family with sequence similarity 160, member B1 [Source:HGNC Symbol;Acc:29320]	0.09	0.03	0.19
MRC2	mannose receptor, C type 2 [Source:HGNC Symbol;Acc:16875]	0.12	0.04	0.22
DUOX1	dual oxidase 1 [Source:HGNC Symbol;Acc:3062]	0.07	0.15	0.25

GCNT2	glucosaminyl (N-acetyl) transferase 2, I-branching enzyme (I blood group) [Source:HGNC Symbol;Acc:4204]	0.04	0.16	0.26
BCL6B	B-cell CLL/lymphoma 6, member B [Source:HGNC Symbol;Acc:1002]	0.06	0.21	0.31
CFTR	cystic fibrosis transmembrane conductance regulator (ATP-binding cassette sub-family C, member 7) [Source:HGNC Symbol;Acc:1884]	0.06	0.23	0.33
NBLA00301	RP11-471J12.1	0.08	0.05	0.18
BEST4	bestrophin 4 [Source:HGNC Symbol;Acc:17106]	0.9	0.9	1
BDKRB1	bradykinin receptor B1 [Source:HGNC Symbol;Acc:1029]	0.87	0.87	0.97
DEF8	differentially expressed in FDCP 8 homolog (mouse) [Source:HGNC Symbol;Acc:25969]	0.37	0.37	0.47
KISS1	KiSS-1 metastasis-suppressor [Source:HGNC Symbol;Acc:6341]	0.84	0.83	0.94
B3GNT5	UDP-GlcNAc:betaGal beta-1,3-N-acetylglucosaminyltransferase 5 [Source:HGNC Symbol;Acc:15684]	0.77	0.76	0.87
CMA1	chymase 1, mast cell [Source:HGNC Symbol;Acc:2097]	0.89	0.9	1
ZDHHC23	zinc finger, DHHC-type containing 23 [Source:HGNC Symbol;Acc:28654]	0.25	0.26	0.36
IQCH	IQ motif containing H [Source:HGNC Symbol;Acc:25721]	0.84	0.82	0.94
C7orf34	chromosome 7 open reading frame 34 [Source:HGNC Symbol;Acc:21750]	0.64	0.62	0.74
NALCN	sodium leak channel, non-selective [Source:HGNC Symbol;Acc:19082]	0.62	0.64	0.74
C15orf27	chromosome 15 open reading frame 27 [Source:HGNC Symbol;Acc:26763]	0.24	0.26	0.36
LCTL	lactase-like [Source:HGNC Symbol;Acc:15583]	0.63	0.65	0.75
ADAMTSL3	ADAMTS-like 3 [Source:HGNC Symbol;Acc:14633]	0.26	0.28	0.38
GPC2	glypican 2 [Source:HGNC Symbol;Acc:4450]	0.12	0.14	0.24
GAS2	growth arrest-specific 2 [Source:HGNC Symbol;Acc:4167]	0.66	0.63	0.76
TEP1	telomerase-associated protein 1 [Source:HGNC Symbol;Acc:11726]	0.34	0.31	0.44
IFITM5	interferon induced transmembrane protein 5 [Source:HGNC Symbol;Acc:16644]	0.8	0.84	0.94
ALOX15	arachidonate 15-lipoxygenase [Source:HGNC Symbol;Acc:433]	0.32	0.28	0.42
ELAVL4	ELAV (embryonic lethal, abnormal vision, Drosophila)-like 4 [Source:HGNC Symbol;Acc:3315]	0.67	0.63	0.77
KLF5	Kruppel-like factor 5 (intestinal) [Source:HGNC Symbol;Acc:6349]	0.54	0.49	0.64
SLC35C1	solute carrier family 35, member C1 [Source:HGNC Symbol;Acc:20197]	0.9	0.85	1
SP7	Sp7 transcription factor [Source:HGNC Symbol;Acc:17321]	0.76	0.71	0.86
C7orf65	chromosome 7 open reading frame 65 [Source:HGNC Symbol;Acc:34432]	0.78	0.72	0.88
STARD13	StAR-related lipid transfer (START) domain containing 13 [Source:HGNC Symbol;Acc:19164]	0.67	0.74	0.84
CRYGD	crystallin, gamma D [Source:HGNC Symbol;Acc:2411]	0.86	0.77	0.96
SLC12A8	solute carrier family 12 (potassium/chloride transporters), member 8 [Source:HGNC Symbol;Acc:15595]	0.78	0.87	0.97
SLC13A4	solute carrier family 13 (sodium/sulfate symporters), member 4 [Source:HGNC Symbol;Acc:15827]	0.52	0.61	0.71
GSTM2	glutathione S-transferase mu 2 (muscle) [Source:HGNC Symbol;Acc:4634]	0.6	0.72	0.82
CD200R1	CD200 receptor 1 [Source:HGNC Symbol;Acc:24235]	0.9	0.77	1
FXYD5	FXYD domain containing ion transport regulator 5 [Source:HGNC Symbol;Acc:4029]	0.74	0.59	0.84
CDH15	cadherin 15, type 1, M-cadherin (myotubule) [Source:HGNC Symbol;Acc:1754]	0.09	0.27	0.37
FAM71F2	family with sequence similarity 71, member F2 [Source:HGNC Symbol;Acc:27998]	0.9	0.69	1
KCNIP2	Kv channel interacting protein 2 [Source:HGNC Symbol;Acc:15522]	0.4	0.64	0.74
MTMR8	myotubularin related protein 8 [Source:HGNC Symbol;Acc:16825]	0.67	0.42	0.77
PYGO1	pygopus homolog 1 (Drosophila) [Source:HGNC Symbol;Acc:30256]	0.83	0.84	0.93
DCTD	dCMP deaminase [Source:HGNC Symbol;Acc:2710]	0.82	0.8	0.91
S100A4	S100 calcium binding protein A4 [Source:HGNC Symbol;Acc:10494]	0.59	0.72	0.81
RCSD1	RCSD domain containing 1 [Source:HGNC Symbol;Acc:28310]	0.42	0.58	0.67
LDLRAD1	low density lipoprotein receptor class A domain containing 1 [Source:HGNC Symbol;Acc:32069]	0.72	0.55	0.81
MLC1	megalencephalic leukoencephalopathy with subcortical cysts 1 [Source:HGNC Symbol;Acc:17082]	0.71	0.54	0.8
KLHDC8A	kelch domain containing 8A [Source:HGNC Symbol;Acc:25573]	0.71	0.25	0.8
ZDHHC15	zinc finger, DHHC-type containing 15 [Source:HGNC Symbol;Acc:20342]	0.46	0.46	0.55
GUCY1B3	guanylate cyclase 1, soluble, beta 3 [Source:HGNC Symbol;Acc:4687]	0.35	0.37	0.46
WDYHV1	WDYHV motif containing 1 [Source:HGNC Symbol;Acc:25490]	0.41	0.38	0.5
CYHR1	cysteine/histidine-rich 1 [Source:HGNC Symbol;Acc:17806]	0.41	0.38	0.5
LOC554223	histocompatibility antigen-related	0.18	0.15	0.27
SLC5A8	solute carrier family 5 (iodide transporter), member 8 [Source:HGNC Symbol;Acc:19119]	0.33	0.37	0.46
EPHA8	EPH receptor A8 [Source:HGNC Symbol;Acc:3391]	0.19	0.14	0.28
KIF21B	kinesin family member 21B [Source:HGNC Symbol;Acc:29442]	0.3	0.24	0.39
DUSP9	dual specificity phosphatase 9 [Source:HGNC Symbol;Acc:3076]	0.46	0.21	0.55
CDHR2	cadherin-related family member 2 [Source:HGNC Symbol;Acc:18231]	0.04	0.08	0.17
IFFO1	intermediate filament family orphan 1 [Source:HGNC Symbol;Acc:24970]	0.08	0.01	0.17
EML2	echinoderm microtubule associated protein like 2 [Source:HGNC Symbol;Acc:18035]	0.02	0.02	0.11
IRF2BP1	interferon regulatory factor 2 binding protein 1 [Source:HGNC Symbol;Acc:21728]	0	0	0.09
BOK	BCL2-related ovarian killer [Source:HGNC Symbol;Acc:1087]	0.02	0.02	0.11
CXCL2	chemokine (C-X-C motif) ligand 2 [Source:HGNC Symbol;Acc:4603]	0.05	0.06	0.15
CKAP2	cytoskeleton associated protein 2 [Source:HGNC Symbol;Acc:1990]	0.02	0.01	0.11

OTX2	orthodenticle homeobox 2 [Source:HGNC Symbol;Acc:8522]	0.01	0.02	0.11
COL4A1	collagen, type IV, alpha 1 [Source:HGNC Symbol;Acc:2202]	0.09	0.07	0.18
COL4A2	collagen, type IV, alpha 2 [Source:HGNC Symbol;Acc:2203]	0.09	0.07	0.18
ZNF672	zinc finger protein 672 [Source:HGNC Symbol;Acc:26179]	0.09	0.07	0.18
PRSS35	protease, serine, 35 [Source:HGNC Symbol;Acc:21387]	0	0.02	0.11
RFTN2	raftlin family member 2 [Source:HGNC Symbol;Acc:26402]	0.21	0.23	0.32
SH3GLB1	SH3-domain GRB2-like endophilin B1 [Source:HGNC Symbol;Acc:10833]	0.12	0.15	0.24
CYR61	cysteine-rich, angiogenic inducer, 61 [Source:HGNC Symbol;Acc:2654]	0.03	0.07	0.16
PPP2R3A	protein phosphatase 2, regulatory subunit B'', alpha [Source:HGNC Symbol;Acc:9307]	0.15	0.1	0.24
DUOXA1	dual oxidase maturation factor 1 [Source:HGNC Symbol;Acc:26507]	0.13	0.23	0.32
CHIT1	chitinase 1 (chitotriosidase) [Source:HGNC Symbol;Acc:1936]	0.87	0.87	0.96
C1orf100	chromosome 1 open reading frame 100 [Source:HGNC Symbol;Acc:30435]	0.78	0.78	0.87
TMEM132A	transmembrane protein 132A [Source:HGNC Symbol;Acc:31092]	0.79	0.79	0.88
LAMP2	lysosomal-associated membrane protein 2 [Source:HGNC Symbol;Acc:6501]	0.38	0.38	0.47
FAM123B	APC membrane recruitment protein 1 [Source:HGNC Symbol;Acc:26837]	0.33	0.33	0.42
DPT	dermatopontin [Source:HGNC Symbol;Acc:3011]	0.91	0.9	1
DNAJB8	DnaJ (Hsp40) homolog, subfamily B, member 8 [Source:HGNC Symbol;Acc:23699]	0.75	0.74	0.84
LYL1	lymphoblastic leukemia derived sequence 1 [Source:HGNC Symbol;Acc:6734]	0.67	0.68	0.77
CCDC152	coiled-coil domain containing 152 [Source:HGNC Symbol;Acc:34438]	0.53	0.54	0.63
MAL2	mal, T-cell differentiation protein 2 (gene/pseudogene) [Source:HGNC Symbol;Acc:13634]	0.66	0.67	0.76
SYT14	synaptotagmin XIV [Source:HGNC Symbol;Acc:23143]	0.39	0.37	0.48
KRT73	keratin 73 [Source:HGNC Symbol;Acc:28928]	0.85	0.83	0.94
CYP1A2	cytochrome P450, family 1, subfamily A, polypeptide 2 [Source:HGNC Symbol;Acc:2596]	0.87	0.85	0.96
NRBF2	nuclear receptor binding factor 2 [Source:HGNC Symbol;Acc:19692]	0.62	0.64	0.73
MMP10	matrix metallopeptidase 10 (stromelysin 2) [Source:HGNC Symbol;Acc:7156]	0.86	0.83	0.95
RHBDL2	rhomboid, veinlet-like 2 (Drosophila) [Source:HGNC Symbol;Acc:16083]	0.75	0.72	0.84
PDZK1IP1	PDZK1 interacting protein 1 [Source:HGNC Symbol;Acc:16887]	0.81	0.78	0.9
TTLL10	tubulin tyrosine ligase-like family, member 10 [Source:HGNC Symbol;Acc:26693]	0.27	0.24	0.36
SEMG1	semenogelin I [Source:HGNC Symbol;Acc:10742]	0.86	0.83	0.95
CTLA4	cytotoxic T-lymphocyte-associated protein 4 [Source:HGNC Symbol;Acc:2505]	0.88	0.91	1
RNF133	ring finger protein 133 [Source:HGNC Symbol;Acc:21154]	0.87	0.9	0.99
LTB	lymphotoxin beta (TNF superfamily, member 3) [Source:HGNC Symbol;Acc:6711]	0.85	0.81	0.94
PXDNL	peroxidasin homolog (Drosophila)-like [Source:HGNC Symbol;Acc:26359]	0.56	0.6	0.69
ACOT9	acyl-CoA thioesterase 9 [Source:HGNC Symbol;Acc:17152]	0.49	0.45	0.58
CSK	c-src tyrosine kinase [Source:HGNC Symbol;Acc:2444]	0.22	0.26	0.35
APEX2	APEX nuclease (apurinic/apyrimidinic endonuclease) 2 [Source:HGNC Symbol;Acc:17889]	0.39	0.35	0.48
GEMIN4	gem (nuclear organelle) associated protein 4 [Source:HGNC Symbol;Acc:15717]	0.69	0.73	0.82
PIN1	peptidylprolyl cis/trans isomerase, NIMA-interacting 1 [Source:HGNC Symbol;Acc:8988]	0.82	0.86	0.95
GTF2A1L	general transcription factor IIA, 1-like [Source:HGNC Symbol;Acc:30727]	0.7	0.74	0.83
MT1B	metallothionein 1B [Source:HGNC Symbol;Acc:7394]	0.86	0.81	0.95
PLS3	plastin 3 [Source:HGNC Symbol;Acc:9091]	0.52	0.47	0.61
TNS4	tensin 4 [Source:HGNC Symbol;Acc:24352]	0.7	0.75	0.84
IL13RA1	interleukin 13 receptor, alpha 1 [Source:HGNC Symbol;Acc:5974]	0.62	0.56	0.71
TMSB15B	thymosin beta 15B [Source:HGNC Symbol;Acc:28612]	0.49	0.43	0.58
CXCL6	chemokine (C-X-C motif) ligand 6 [Source:HGNC Symbol;Acc:10643]	0.42	0.48	0.57
SSTR4	somatostatin receptor 4 [Source:HGNC Symbol;Acc:11333]	0.81	0.75	0.9
DUSP28	dual specificity phosphatase 28 [Source:HGNC Symbol;Acc:33237]	0.7	0.76	0.85
OXT	oxytocin/neurophysin I prepropeptide [Source:HGNC Symbol;Acc:8528]	0.85	0.91	1
PLEK	pleckstrin [Source:HGNC Symbol;Acc:9070]	0.88	0.8	0.97
GSTK1	glutathione S-transferase kappa 1 [Source:HGNC Symbol;Acc:16906]	0.62	0.53	0.71
RASAL1	RAS protein activator like 1 (GAP1 like) [Source:HGNC Symbol;Acc:9873]	0.11	0.2	0.29
ENPP3	ectonucleotide pyrophosphatase/phosphodiesterase 3 [Source:HGNC Symbol;Acc:3358]	0.81	0.72	0.9
THY1	Thy-1 cell surface antigen [Source:HGNC Symbol;Acc:11801]	0.55	0.65	0.74
DNM1P35	DNM1 pseudogene 35 [Source:HGNC Symbol;Acc:35182]	0.91	0.8	1
DEFB132	defensin, beta 132 [Source:HGNC Symbol;Acc:33806]	0.62	0.51	0.71
SLC44A2	solute carrier family 44, member 2 [Source:HGNC Symbol;Acc:17292]	0.38	0.23	0.47
SLC19A1	solute carrier family 19 (folate transporter), member 1 [Source:HGNC Symbol;Acc:10937]	0.52	0.32	0.61
AZI1	5-azacytidine induced 1 [Source:HGNC Symbol;Acc:29511]	0.77	0.57	0.86
ABCA6	ATP-binding cassette, sub-family A (ABC1), member 6 [Source:HGNC Symbol;Acc:36]	0.71	0.91	1
IL8	interleukin 8 [Source:HGNC Symbol;Acc:6025]	0.91	0.7	1
CNTN5	contactin 5 [Source:HGNC Symbol;Acc:2175]	0.32	0.09	0.41
ZFP42	ZFP42 zinc finger protein [Source:HGNC Symbol;Acc:30949]	0.41	0.76	0.85
IL17B	interleukin 17B [Source:HGNC Symbol;Acc:5982]	0.85	0.44	0.94
DSG3	desmoglein 3 [Source:HGNC Symbol;Acc:3050]	0.85	0.85	0.93
TMEM63C	transmembrane protein 63C [Source:HGNC Symbol;Acc:23787]	0.47	0.46	0.55

CD34	CD34 molecule [Source:HGNC Symbol;Acc:1662]	0.46	0.48	0.56
IKZF3	IKAROS family zinc finger 3 (Aiolos) [Source:HGNC Symbol;Acc:13178]	0.6	0.58	0.68
C19orf35	chromosome 19 open reading frame 35 [Source:HGNC Symbol;Acc:24793]	0.85	0.83	0.93
BOLL	bol, boule-like (Drosophila) [Source:HGNC Symbol;Acc:14273]	0.69	0.71	0.79
ARRB2	arrestin, beta 2 [Source:HGNC Symbol;Acc:712]	0.56	0.59	0.67
TAS2R60	taste receptor, type 2, member 60 [Source:HGNC Symbol;Acc:20639]	0.79	0.82	0.9
TBX10	T-box 10 [Source:HGNC Symbol;Acc:11593]	0.58	0.54	0.66
LMO3	LIM domain only 3 (rhombotin-like 2) [Source:HGNC Symbol;Acc:6643]	0.53	0.57	0.65
PHYHIP	phytanoyl-CoA 2-hydroxylase interacting protein [Source:HGNC Symbol;Acc:16865]	0.55	0.6	0.68
SYCP1	synaptonemal complex protein 1 [Source:HGNC Symbol;Acc:11487]	0.52	0.58	0.66
C22orf32	chromosome 22 open reading frame 32 [Source:HGNC Symbol;Acc:25055]	0.6	0.53	0.68
ZBTB4	zinc finger and BTB domain containing 4 [Source:HGNC Symbol;Acc:23847]	0.82	0.73	0.9
GLT25D2	glycosyltransferase 25 domain containing 2 [Source:HGNC Symbol;Acc:16790]	0.73	0.61	0.81
SLC34A3	solute carrier family 34 (sodium phosphate), member 3 [Source:HGNC Symbol;Acc:20305]	0.82	0.7	0.9
TIAM2	T-cell lymphoma invasion and metastasis 2 [Source:HGNC Symbol;Acc:11806]	0.6	0.47	0.68
RAB17	RAB17, member RAS oncogene family [Source:HGNC Symbol;Acc:16523]	0.45	0.59	0.67
CD1D	CD1d molecule [Source:HGNC Symbol;Acc:1637]	0.53	0.7	0.78
VSIG8	chromosome 1 open reading frame 204 [Source:HGNC Symbol;Acc:27647]	0.82	0.55	0.9
CDRT1	CMT duplicated region transcript 1; Uncharacterized protein [Source:UniProtKB/TrEMBL;Acc:Q9BXD7]	0.58	0.24	0.66
ZC3HAV1L	zinc finger CCCH-type, antiviral 1-like [Source:HGNC Symbol;Acc:22423]	0.37	0.73	0.81
TLX1	T-cell leukemia homeobox 1 [Source:HGNC Symbol;Acc:5056]	0.32	0.32	0.4
TLX1NB	TLX1 neighbor [Source:HGNC Symbol;Acc:37183]	0.32	0.32	0.4
FRMD4A	FERM domain containing 4A [Source:HGNC Symbol;Acc:25491]	0.41	0.41	0.49
NFKBIZ	nuclear factor of kappa light polypeptide gene enhancer in B-cells inhibitor, zeta [Source:HGNC Symbol;Acc:29805]	0.17	0.18	0.26
FNIP2	folliculin interacting protein 2 [Source:HGNC Symbol;Acc:29280]	0.31	0.32	0.4
TMEM164	transmembrane protein 164 [Source:HGNC Symbol;Acc:26217]	0.41	0.43	0.51
RARA	retinoic acid receptor, alpha [Source:HGNC Symbol;Acc:9864]	0.03	0.06	0.14
ABHD14A	abhydrolase domain containing 14A [Source:HGNC Symbol;Acc:24538]	0.09	0.06	0.17
USH1C	Usher syndrome 1C (autosomal recessive, severe) [Source:HGNC Symbol;Acc:12597]	0.17	0.2	0.28
LPAR1	lysophosphatidic acid receptor 1 [Source:HGNC Symbol;Acc:3166]	0.15	0.18	0.26
RESP18	regulated endocrine-specific protein 18 [Source:HGNC Symbol;Acc:33762]	0.12	0.08	0.2
A4GALT	alpha 1,4-galactosyltransferase [Source:HGNC Symbol;Acc:18149]	0.02	0.06	0.14
ASS1	argininosuccinate synthase 1 [Source:HGNC Symbol;Acc:758]	0.41	0.46	0.54
NDUFV3	NADH dehydrogenase (ubiquinone) flavoprotein 3, 10kDa [Source:HGNC Symbol;Acc:7719]	0.12	0.03	0.2
LDHC	lactate dehydrogenase C [Source:HGNC Symbol;Acc:6544]	0.19	0.29	0.37
PDE4B	phosphodiesterase 4B, cAMP-specific [Source:HGNC Symbol;Acc:8781]	0.19	0.08	0.27
CLEC14A	C-type lectin domain family 14, member A [Source:HGNC Symbol;Acc:19832]	0.12	0.25	0.33
CELF3	CUGBP, Elav-like family member 3 [Source:HGNC Symbol;Acc:11967]	0.32	0.11	0.4
LIN54	lin-54 homolog (C. elegans) [Source:HGNC Symbol;Acc:25397]	0.02	0	0.1
FBXL14	F-box and leucine-rich repeat protein 14 [Source:HGNC Symbol;Acc:28624]	0.11	0.07	0.19
MPDU1	mannose-P-dolichol utilization defect 1 [Source:HGNC Symbol;Acc:7207]	0.2	0.22	0.3
EFHD2	EF-hand domain family, member D2 [Source:HGNC Symbol;Acc:28670]	0.13	0.11	0.21
TMEM179	transmembrane protein 179 [Source:HGNC Symbol;Acc:20137]	0.23	0.16	0.31
NTAN1	N-terminal asparagine amidase [Source:HGNC Symbol;Acc:29909]	0.17	0.07	0.25
NME3	NME/NM23 nucleoside diphosphate kinase 3 [Source:HGNC Symbol;Acc:7851]	0.06	0.16	0.24
HEPH	hephaestin [Source:HGNC Symbol;Acc:4866]	0.33	0.33	0.41
NMUR1	neuromedin U receptor 1 [Source:HGNC Symbol;Acc:4518]	0.56	0.56	0.64
ITLN2	intelectin 2 [Source:HGNC Symbol;Acc:20599]	0.91	0.9	0.99
C20orf152	N/A	0.79	0.78	0.87
AOC3	amine oxidase, copper containing 3 (vascular adhesion protein 1) [Source:HGNC Symbol;Acc:550]	0.85	0.86	0.94
GFRA4	GDNF family receptor alpha 4 [Source:HGNC Symbol;Acc:13821]	0.61	0.62	0.7
UGT2A3	UDP glucuronosyltransferase 2 family, polypeptide A3 [Source:HGNC Symbol;Acc:28528]	0.85	0.86	0.94
PPFIA4	protein tyrosine phosphatase, receptor type, f polypeptide (PTPRF), interacting protein (liprin), alpha 4 [Source:HGNC Symbol;Acc:9248]	0.9	0.88	0.98
LMO1	LIM domain only 1 (rhombotin 1) [Source:HGNC Symbol;Acc:6641]	0.63	0.61	0.71
LRRC33	leucine rich repeat containing 33 [Source:HGNC Symbol;Acc:24613]	0.66	0.64	0.74
RLTPR	RGD motif, leucine rich repeats, tropomodulin domain and proline-rich containing [Source:HGNC Symbol;Acc:27089]	0.9	0.92	1
IRF8	interferon regulatory factor 8 [Source:HGNC Symbol;Acc:5358]	0.59	0.61	0.69
NEB	nebulin [Source:HGNC Symbol;Acc:7720]	0.86	0.88	0.96
MYL7	myosin, light chain 7, regulatory [Source:HGNC Symbol;Acc:21719]	0.87	0.9	0.98
COL6A3	collagen, type VI, alpha 3 [Source:HGNC Symbol;Acc:2213]	0.89	0.86	0.97
RGAG4	retrotransposon gag domain containing 4 [Source:HGNC Symbol;Acc:29430]	0.56	0.53	0.64
RORC	RAR-related orphan receptor C [Source:HGNC Symbol;Acc:10260]	0.52	0.55	0.63
TRPV2	transient receptor potential cation channel, subfamily V, member 2 [Source:HGNC Symbol;Acc:18082]	0.62	0.65	0.73
SCN5A	sodium channel, voltage-gated, type V, alpha subunit [Source:HGNC Symbol;Acc:10593]	0.61	0.65	0.73

C1R	complement component 1, r subcomponent [Source:HGNC Symbol;Acc:1246]	0.78	0.74	0.86
AAGAB	alpha- and gamma-adaptin binding protein [Source:HGNC Symbol;Acc:25662]	0.88	0.92	1
OGT	O-linked N-acetylglucosamine (GlcNAc) transferase [Source:HGNC Symbol;Acc:8127]	0.51	0.46	0.59
TSPO	translocator protein (18kDa) [Source:HGNC Symbol;Acc:1158]	0.34	0.39	0.47
DOK3	docking protein 3 [Source:HGNC Symbol;Acc:24583]	0.89	0.84	0.97
RSPO4	R-spondin 4 [Source:HGNC Symbol;Acc:16175]	0.63	0.68	0.76
SNAPC1	small nuclear RNA activating complex, polypeptide 1, 43kDa [Source:HGNC Symbol;Acc:11134]	0.51	0.45	0.59
CSTA	cystatin A (stefin A) [Source:HGNC Symbol;Acc:2481]	0.86	0.92	1
TNFSF8	tumor necrosis factor (ligand) superfamily, member 8 [Source:HGNC Symbol;Acc:11938]	0.74	0.8	0.88
GAL	galanin/GMAP prepropeptide [Source:HGNC Symbol;Acc:4114]	0.43	0.5	0.58
ALX3	ALX homeobox 3 [Source:HGNC Symbol;Acc:449]	0.79	0.71	0.87
CHRNA2	cholinergic receptor, nicotinic, alpha 2 (neuronal) [Source:HGNC Symbol;Acc:1956]	0.66	0.58	0.74
SNX32	sorting nexin 32 [Source:HGNC Symbol;Acc:26423]	0.47	0.55	0.63
ADCY9	adenylate cyclase 9 [Source:HGNC Symbol;Acc:240]	0.57	0.65	0.73
CSMD1	CUB and Sushi multiple domains 1 [Source:HGNC Symbol;Acc:14026]	0.77	0.68	0.85
PHOSPHO1	phosphatase, orphan 1 [Source:HGNC Symbol;Acc:16815]	0.52	0.43	0.6
SLC47A1	solute carrier family 47, member 1 [Source:HGNC Symbol;Acc:25588]	0.28	0.19	0.36
LOC150568	uncharacterized	0.3	0.4	0.48
C3orf72	chromosome 3 open reading frame 72 [Source:HGNC Symbol;Acc:34428]	0.3	0.4	0.48
FOXL2	forkhead box L2 [Source:HGNC Symbol;Acc:1092]	0.3	0.4	0.48
C7	complement component 7 [Source:HGNC Symbol;Acc:1346]	0.8	0.92	1
UPB1	ureidopropionase, beta [Source:HGNC Symbol;Acc:16297]	0.4	0.52	0.6
ENPP4	ectonucleotide pyrophosphatase/phosphodiesterase 4 (putative) [Source:HGNC Symbol;Acc:3359]	0.27	0.14	0.35
ZMYM3	zinc finger, MYM-type 3 [Source:HGNC Symbol;Acc:13054]	0.56	0.42	0.64
KAT2A	K(lysine) acetyltransferase 2A [Source:HGNC Symbol;Acc:4201]	0.88	0.71	0.96
AQPEP	Aminopeptidase Q [Source:UniProtKB/Swiss-Prot;Acc:Q6Q4G3]	0.44	0.62	0.7
ASPA	aspartoacylase [Source:HGNC Symbol;Acc:756]	0.87	0.68	0.95
DNAH1	dynein, axonemal, heavy chain 1 [Source:HGNC Symbol;Acc:2940]	0.77	0.58	0.85
GNRH1	gonadotropin-releasing hormone 1 (luteinizing-releasing hormone) [Source:HGNC Symbol;Acc:4419]	0.7	0.92	1
SCG2	secretogranin II [Source:HGNC Symbol;Acc:10575]	0.5	0.14	0.58
GRIK5	glutamate receptor, ionotropic, kainate 5 [Source:HGNC Symbol;Acc:4583]	0.68	0.29	0.76
TRANK1	tetratricopeptide repeat and ankyrin repeat containing 1 [Source:HGNC Symbol;Acc:29011]	0.11	0.51	0.59
RGMA	RGM domain family, member A [Source:HGNC Symbol;Acc:30308]	0.9	0.33	0.98
ZBTB44	zinc finger and BTB domain containing 44 [Source:HGNC Symbol;Acc:25001]	0.83	0.83	0.9
RNF40	ring finger protein 40, E3 ubiquitin protein ligase [Source:HGNC Symbol;Acc:16867]	0.86	0.86	0.93
CLEC5A	C-type lectin domain family 5, member A [Source:HGNC Symbol;Acc:2054]	0.82	0.82	0.89
GYS2	glycogen synthase 2 (liver) [Source:HGNC Symbol;Acc:4707]	0.7	0.69	0.77
HHLA2	HERV-H LTR-associating 2 [Source:HGNC Symbol;Acc:4905]	0.85	0.84	0.92
CCL28	chemokine (C-C motif) ligand 28 [Source:HGNC Symbol;Acc:17700]	0.84	0.83	0.91
C6orf10	chromosome 6 open reading frame 10 [Source:HGNC Symbol;Acc:13922]	0.84	0.83	0.91
GIPC2	GIPC PDZ domain containing family, member 2 [Source:HGNC Symbol;Acc:18177]	0.84	0.85	0.92
C11orf68	chromosome 11 open reading frame 68 [Source:HGNC Symbol;Acc:28801]	0.85	0.86	0.93
SFTPB	surfactant protein B [Source:HGNC Symbol;Acc:10801]	0.85	0.86	0.93
FAM165B	small integral membrane protein 11 [Source:HGNC Symbol;Acc:1293]	0.82	0.83	0.9
ZNF773	zinc finger protein 773 [Source:HGNC Symbol;Acc:30487]	0.81	0.83	0.9
CLEC9A	C-type lectin domain family 9, member A [Source:HGNC Symbol;Acc:26705]	0.83	0.85	0.92
ABP1	amiloride binding protein 1 (amine oxidase (copper-containing)) [Source:HGNC Symbol;Acc:80]	0.83	0.8	0.9
COL20A1	collagen, type XX, alpha 1 [Source:HGNC Symbol;Acc:14670]	0.8	0.83	0.9
FAM120C	family with sequence similarity 120C [Source:HGNC Symbol;Acc:16949]	0.48	0.45	0.55
BZRAP1	benzodiazapine receptor (peripheral) associated protein 1 [Source:HGNC Symbol;Acc:16831]	0.72	0.69	0.79
MAP2K4	mitogen-activated protein kinase kinase 4 [Source:HGNC Symbol;Acc:6844]	0.58	0.61	0.68
C10orf108	proline rich 26 [Source:HGNC Symbol;Acc:30724]	0.81	0.86	0.93
MYEOV	myeloma overexpressed (in a subset of t(11;14) positive multiple myelomas) [Source:HGNC Symbol;Acc:7563]	0.77	0.82	0.89
CD300E	CD300e molecule [Source:HGNC Symbol;Acc:28874]	0.83	0.76	0.9
GNA14	guanine nucleotide binding protein (G protein), alpha 14 [Source:HGNC Symbol;Acc:4382]	0.66	0.73	0.8
PPM1M	protein phosphatase, Mg2+/Mn2+ dependent, 1M [Source:HGNC Symbol;Acc:26506]	0.75	0.83	0.9
MTA1	metastasis associated 1 [Source:HGNC Symbol;Acc:7410]	0.83	0.71	0.9
VAMP1	vesicle-associated membrane protein 1 (synaptobrevin 1) [Source:HGNC Symbol;Acc:12642]	0.74	0.61	0.81
KRTAP10-4	keratin associated protein 10-4 [Source:HGNC Symbol;Acc:20521]	0.83	0.69	0.9
CAMK1	calcium/calmodulin-dependent protein kinase I [Source:HGNC Symbol;Acc:1459]	0.42	0.58	0.65

SVOP	SV2 related protein homolog (rat) [Source:HGNC Symbol;Acc:25417]	0.85	0.67	0.92
CD36	CD36 molecule (thrombospondin receptor) [Source:HGNC Symbol;Acc:1663]	0.86	0.66	0.93
CTHRC1	collagen triple helix repeat containing 1 [Source:HGNC Symbol;Acc:18831]	0.85	0.6	0.92
ATP9A	ATPase, class II, type 9A [Source:HGNC Symbol;Acc:13540]	0.72	0.45	0.79
HOXC13	homeobox C13 [Source:HGNC Symbol;Acc:5125]	0.13	0.13	0.2
ARPC2	actin related protein 2/3 complex, subunit 2, 34kDa [Source:HGNC Symbol;Acc:705]	0	0	0.07
NFE2L2	nuclear factor (erythroid-derived 2)-like 2 [Source:HGNC Symbol;Acc:7782]	0	0	0.07
CLVS1	clavesin 1 [Source:HGNC Symbol;Acc:23139]	0.01	0.01	0.08
C1QTNF2	C1q and tumor necrosis factor related protein 2 [Source:HGNC Symbol;Acc:14325]	0.28	0.29	0.36
C3orf70	chromosome 3 open reading frame 70 [Source:HGNC Symbol;Acc:33731]	0.01	0	0.08
MSH5	mutS homolog 5 (E. coli) [Source:HGNC Symbol;Acc:7328]	0.04	0.03	0.11
IZUMO1	izumo sperm-egg fusion 1 [Source:HGNC Symbol;Acc:28539]	0.03	0.04	0.11
ST3GAL3	ST3 beta-galactoside alpha-2,3-sialyltransferase 3 [Source:HGNC Symbol;Acc:10866]	0.16	0.15	0.23
FJX1	four jointed box 1 (Drosophila) [Source:HGNC Symbol;Acc:17166]	0.27	0.26	0.34
C3orf37	chromosome 3 open reading frame 37 [Source:HGNC Symbol;Acc:24446]	0.39	0.38	0.46
ANGPTL2	angiopoietin-like 2 [Source:HGNC Symbol;Acc:490]	0.06	0.07	0.14
IL23A	interleukin 23, alpha subunit p19 [Source:HGNC Symbol;Acc:15488]	0.41	0.39	0.48
TUBGCP5	tubulin, gamma complex associated protein 5 [Source:HGNC Symbol;Acc:18600]	0.06	0.04	0.13
SLC26A5	solute carrier family 26, member 5 (prestin) [Source:HGNC Symbol;Acc:9359]	0.15	0.13	0.22
GPR19	G protein-coupled receptor 19 [Source:HGNC Symbol;Acc:4473]	0.27	0.3	0.37
SYTL4	synaptotagmin-like 4 [Source:HGNC Symbol;Acc:15588]	0.4	0.43	0.5
ALDH1A2	aldehyde dehydrogenase 1 family, member A2 [Source:HGNC Symbol;Acc:15472]	0.13	0.1	0.2
NFKB1	nuclear factor of kappa light polypeptide gene enhancer in B-cells 1 [Source:HGNC Symbol;Acc:7794]	0.06	0.03	0.13
METTL7B	methyltransferase like 7B [Source:HGNC Symbol;Acc:28276]	0.16	0.19	0.26
CXCL14	chemokine (C-X-C motif) ligand 14 [Source:HGNC Symbol;Acc:10640]	0.04	0.07	0.14
GFI1B	growth factor independent 1B transcription repressor [Source:HGNC Symbol;Acc:4238]	0.22	0.25	0.32
PPDPF	pancreatic progenitor cell differentiation and proliferation factor homolog (zebrafish) [Source:HGNC Symbol;Acc:16142]	0.32	0.36	0.43
ADH1B	alcohol dehydrogenase 1B (class I), beta polypeptide [Source:HGNC Symbol;Acc:250]	0.27	0.31	0.38
KLHL14	kelch-like family member 14 [Source:HGNC Symbol;Acc:29266]	0	0.04	0.11
SNHG12	small nucleolar RNA host gene 12 (non-protein coding) [Source:HGNC Symbol;Acc:30062]	0.07	0.02	0.14
FLYWCH2	FLYWCH family member 2 [Source:HGNC Symbol;Acc:25178]	0.16	0.1	0.23
ABCA7	ATP-binding cassette, sub-family A (ABC1), member 7 [Source:HGNC Symbol;Acc:37]	0.18	0.1	0.25
TSPAN17	tetraspanin 17 [Source:HGNC Symbol;Acc:13594]	0.39	0.31	0.46
C15orf38	chromosome 15 open reading frame 38 [Source:HGNC Symbol;Acc:28782]	0.38	0.46	0.53
POMT1	protein-O-mannosyltransferase 1 [Source:HGNC Symbol;Acc:9202]	0.12	0.03	0.19
ZFPM2	zinc finger protein, FOG family member 2 [Source:HGNC Symbol;Acc:16700]	0.09	0.19	0.26
PID1	phosphotyrosine interaction domain containing 1 [Source:HGNC Symbol;Acc:26084]	0.16	0.26	0.33
ANKLE1	ankyrin repeat and LEM domain containing 1 [Source:HGNC Symbol;Acc:26812]	0.24	0.35	0.42
TASP1	taspase, threonine aspartase, 1 [Source:HGNC Symbol;Acc:15859]	0.06	0.18	0.25
SUMO3	SMT3 suppressor of mif two 3 homolog 3 (S. cerevisiae) [Source:HGNC Symbol;Acc:11124]	0.13	0.3	0.37
HK1	hexokinase 1 [Source:HGNC Symbol;Acc:4922]	0.25	0.43	0.5
CBX2	chromobox homolog 2 [Source:HGNC Symbol;Acc:1552]	0.02	0.01	0.09
TMEM17	transmembrane protein 17 [Source:HGNC Symbol;Acc:26623]	0.03	0.05	0.12
TCF3	transcription factor 3 (E2A immunoglobulin enhancer binding factors E12/E47) [Source:HGNC Symbol;Acc:11633]	0.08	0.11	0.18
RTN3	reticulon 3 [Source:HGNC Symbol;Acc:10469]	0.11	0.07	0.18
DPP6	dipeptidyl-peptidase 6 [Source:HGNC Symbol;Acc:3010]	0.05	0	0.12
TMEM154	transmembrane protein 154 [Source:HGNC Symbol;Acc:26489]	0.11	0.05	0.18
RRM2	ribonucleotide reductase M2 [Source:HGNC Symbol;Acc:10452]	0.11	0.03	0.18
CD80	CD80 molecule [Source:HGNC Symbol;Acc:1700]	0.13	0.14	0.21
DTX3L	deltex 3-like (Drosophila) [Source:HGNC Symbol;Acc:30323]	0.11	0.22	0.29
PARP9	poly (ADP-ribose) polymerase family, member 9 [Source:HGNC Symbol;Acc:24118]	0.11	0.22	0.29
LAYN	layilin [Source:HGNC Symbol;Acc:29471]	0.87	0.87	0.94
LAX1	lymphocyte transmembrane adaptor 1 [Source:HGNC Symbol;Acc:26005]	0.89	0.89	0.96
HTN3	histatin 3 [Source:HGNC Symbol;Acc:5284]	0.93	0.93	1
C9orf66	chromosome 9 open reading frame 66 [Source:HGNC Symbol;Acc:26436]	0.77	0.77	0.84
GPR84	G protein-coupled receptor 84 [Source:HGNC Symbol;Acc:4535]	0.87	0.86	0.94
RBP5	retinol binding protein 5, cellular [Source:HGNC Symbol;Acc:15847]	0.52	0.51	0.59
EVPLL	envoplakin-like [Source:HGNC Symbol;Acc:35236]	0.87	0.86	0.94
SNORD17	small nucleolar RNA, C/D box 17 [Source:HGNC Symbol;Acc:32713]	0.88	0.87	0.95
PCDHB13	protocadherin beta 13 [Source:HGNC Symbol;Acc:8684]	0.62	0.61	0.69
COL15A1	collagen, type XV, alpha 1 [Source:HGNC Symbol;Acc:2192]	0.89	0.88	0.96

SLC12A1	solute carrier family 12 (sodium/potassium/chloride transporters), member 1 [Source:HGNC Symbol;Acc:10910]	0.87	0.88	0.95
BTBD2	BTB (POZ) domain containing 2 [Source:HGNC Symbol;Acc:15504]	0.87	0.88	0.95
CCL26	chemokine (C-C motif) ligand 26 [Source:HGNC Symbol;Acc:10625]	0.89	0.9	0.97
STAG3	stromal antigen 3 [Source:HGNC Symbol;Acc:11356]	0.39	0.4	0.47
THEM5	thioesterase superfamily member 5 [Source:HGNC Symbol;Acc:26755]	0.88	0.86	0.95
XPNPEP1	X-prolyl aminopeptidase (aminopeptidase P) 1, soluble [Source:HGNC Symbol;Acc:12822]	0.62	0.6	0.69
ATL1	atlastin GTPase 1 [Source:HGNC Symbol;Acc:11231]	0.93	0.91	1
TRAPPC5	trafficking protein particle complex 5 [Source:HGNC Symbol;Acc:23067]	0.6	0.62	0.69
APOA1	apolipoprotein A-I [Source:HGNC Symbol;Acc:600]	0.9	0.87	0.97
JOSD2	Josephin domain containing 2 [Source:HGNC Symbol;Acc:28853]	0.76	0.73	0.83
UGT2B28	UDP glucuronosyltransferase 2 family, polypeptide B28 [Source:HGNC Symbol;Acc:13479]	0.86	0.89	0.96
C1S	complement component 1, s subcomponent [Source:HGNC Symbol;Acc:1247]	0.87	0.83	0.94
FLJ34503	uncharacterized	0.88	0.84	0.95
OR2B3	olfactory receptor, family 2, subfamily B, member 3 [Source:HGNC Symbol;Acc:8238]	0.9	0.84	0.97
CHGB	chromogranin B (secretogranin 1) [Source:HGNC Symbol;Acc:1930]	0.28	0.21	0.35
YWHAH	tyrosine 3-monooxygenase/tryptophan 5-monooxygenase activation protein, eta polypeptide [Source:HGNC Symbol;Acc:12853]	0.8	0.89	0.96
FAM131B	family with sequence similarity 131, member B [Source:HGNC Symbol;Acc:22202]	0.4	0.5	0.57
ABCD2	ATP-binding cassette, sub-family D (ALD), member 2 [Source:HGNC Symbol;Acc:66]	0.34	0.24	0.41
ANO2	anoctamin 2 [Source:HGNC Symbol;Acc:1183]	0.67	0.78	0.85
OR2G2	olfactory receptor, family 2, subfamily G, member 2 [Source:HGNC Symbol;Acc:15007]	0.88	0.75	0.95
DGKA	diacylglycerol kinase, alpha 80kDa [Source:HGNC Symbol;Acc:2849]	0.54	0.67	0.74
GKN2	gastrokine 2 [Source:HGNC Symbol;Acc:24588]	0.64	0.5	0.71
MOGAT3	monoacylglycerol O-acyltransferase 3 [Source:HGNC Symbol;Acc:23249]	0.56	0.42	0.63
RAB27A	RAB27A, member RAS oncogene family [Source:HGNC Symbol;Acc:9766]	0.91	0.74	0.98
TYMP	thymidine phosphorylase [Source:HGNC Symbol;Acc:3148]	0.49	0.66	0.73
GRM2	glutamate receptor, metabotropic 2 [Source:HGNC Symbol;Acc:4594]	0.79	0.53	0.86
CELA1	chymotrypsin-like elastase family, member 1 [Source:HGNC Symbol;Acc:3308]	0.75	0.48	0.82
GMFG	glia maturation factor, gamma [Source:HGNC Symbol;Acc:4374]	0.75	0.46	0.82
ZNF208	zinc finger protein 208 [Source:HGNC Symbol;Acc:12999]	0.72	0.72	0.78
TMEM184B	transmembrane protein 184B [Source:HGNC Symbol;Acc:1310]	0.5	0.5	0.56
EPS8L3	EPS8-like 3 [Source:HGNC Symbol;Acc:21297]	0.82	0.81	0.88
CLNK	cytokine-dependent hematopoietic cell linker [Source:HGNC Symbol;Acc:17438]	0.93	0.94	1
TSPAN32	tetraspanin 32 [Source:HGNC Symbol;Acc:13410]	0.87	0.86	0.93
C11orf21	chromosome 11 open reading frame 21 [Source:HGNC Symbol;Acc:13231]	0.87	0.86	0.93
SLC14A1	solute carrier family 14 (urea transporter), member 1 (Kidd blood group) [Source:HGNC Symbol;Acc:10918]	0.83	0.82	0.89
LAD1	ladinin 1 [Source:HGNC Symbol;Acc:6472]	0.86	0.87	0.93
VGLL3	vestigial like 3 (Drosophila) [Source:HGNC Symbol;Acc:24327]	0.47	0.48	0.54
PABPC4L	poly(A) binding protein, cytoplasmic 4-like [Source:HGNC Symbol;Acc:31955]	0.74	0.75	0.81
OR8H1	olfactory receptor, family 8, subfamily H, member 1 [Source:HGNC Symbol;Acc:14824]	0.94	0.92	1
ISLR	immunoglobulin superfamily containing leucine-rich repeat [Source:HGNC Symbol;Acc:6133]	0.83	0.81	0.89
HCFC2	host cell factor C2 [Source:HGNC Symbol;Acc:24972]	0.92	0.94	1
BCS1L	BC1 (ubiquinol-cytochrome c reductase) synthesis-like [Source:HGNC Symbol;Acc:1020]	0.49	0.47	0.55
TCF15	transcription factor 15 (basic helix-loop-helix) [Source:HGNC Symbol;Acc:11627]	0.84	0.82	0.9
IFIT2	interferon-induced protein with tetratricopeptide repeats 2 [Source:HGNC Symbol;Acc:5409]	0.83	0.85	0.91
BEX5	brain expressed, X-linked 5 [Source:HGNC Symbol;Acc:27990]	0.59	0.61	0.67
DHH	desert hedgehog [Source:HGNC Symbol;Acc:2865]	0.84	0.81	0.9
C20orf141	transmembrane protein 239 [Source:HGNC Symbol;Acc:40044]	0.82	0.79	0.88
CST9	cystatin 9 (testatin) [Source:HGNC Symbol;Acc:13261]	0.83	0.8	0.89
TLR9	TLR9 [Source:UniProtKB/TrEMBL;Acc:C3W5P5]	0.94	0.91	1
TNNC2	troponin C type 2 (fast) [Source:HGNC Symbol;Acc:11944]	0.91	0.94	1
BFSP2	beaded filament structural protein 2, phakinin [Source:HGNC Symbol;Acc:1041]	0.81	0.84	0.9
C9orf117	chromosome 9 open reading frame 117 [Source:HGNC Symbol;Acc:27843]	0.79	0.82	0.88
HSD3B1	hydroxy-delta-5-steroid dehydrogenase, 3 beta- and steroid delta-isomerase 1 [Source:HGNC Symbol;Acc:5217]	0.87	0.84	0.93
TAC4	tachykinin 4 (hemokinin) [Source:HGNC Symbol;Acc:16641]	0.74	0.71	0.8
KLK4	kallikrein-related peptidase 4 [Source:HGNC Symbol;Acc:6365]	0.87	0.84	0.93
REEP5	receptor accessory protein 5 [Source:HGNC Symbol;Acc:30077]	0.78	0.82	0.88
SERPINA5	serpin peptidase inhibitor, clade A (alpha-1 antiproteinase, antitrypsin), member 5 [Source:HGNC Symbol;Acc:8723]	0.71	0.67	0.77
CD247	CD247 molecule [Source:HGNC Symbol;Acc:1677]	0.87	0.83	0.93
ICAM3	intercellular adhesion molecule 3 [Source:HGNC Symbol;Acc:5346]	0.68	0.73	0.79

PMP22	peripheral myelin protein 22 [Source:HGNC Symbol;Acc:9118]	0.72	0.66	0.78
FCRLB	Fc receptor-like B [Source:HGNC Symbol;Acc:26431]	0.83	0.77	0.89
CHRM1	cholinergic receptor, muscarinic 1 [Source:HGNC Symbol;Acc:1950]	0.84	0.78	0.9
EFHB	EF-hand domain family, member B [Source:HGNC Symbol;Acc:26330]	0.67	0.73	0.79
C19orf69	chromosome 19 open reading frame 69 [Source:HGNC Symbol;Acc:34497]	0.69	0.61	0.75
SUSD3	sushi domain containing 3 [Source:HGNC Symbol;Acc:28391]	0.5	0.58	0.64
CRYAA	crystallin, alpha A [Source:HGNC Symbol;Acc:2388]	0.86	0.77	0.92
GNAT1	guanine nucleotide binding protein (G protein), alpha transducing activity polypeptide 1 [Source:HGNC Symbol;Acc:4393]	0.87	0.78	0.93
GCK	glucokinase (hexokinase 4) [Source:HGNC Symbol;Acc:4195]	0.86	0.76	0.92
PAX8	paired box 8 [Source:HGNC Symbol;Acc:8622]	0.75	0.86	0.92
TRIM58	tripartite motif containing 58 [Source:HGNC Symbol;Acc:24150]	0.83	0.7	0.89
HAVCR2	hepatitis A virus cellular receptor 2 [Source:HGNC Symbol;Acc:18437]	0.84	0.71	0.9
COL9A2	collagen, type IX, alpha 2 [Source:HGNC Symbol;Acc:2218]	0.59	0.72	0.78
CXCL13	chemokine (C-X-C motif) ligand 13 [Source:HGNC Symbol;Acc:10639]	0.87	0.73	0.93
OCA2	oculocutaneous albinism II [Source:HGNC Symbol;Acc:8101]	0.71	0.86	0.92
FAM65A	family with sequence similarity 65, member A [Source:HGNC Symbol;Acc:25836]	0.59	0.74	0.8
HIST1H4L	histone cluster 1, H4a [Source:HGNC Symbol;Acc:4781]	0.58	0.73	0.79
SLC24A5	solute carrier family 24, member 5 [Source:HGNC Symbol;Acc:20611]	0.86	0.64	0.92
FAM114A1	family with sequence similarity 114, member A1 [Source:HGNC Symbol;Acc:25087]	0.27	0.49	0.55
ME3	malic enzyme 3, NADP(+)-dependent, mitochondrial [Source:HGNC Symbol;Acc:6985]	0.53	0.82	0.88
GRIP1	glutamate receptor interacting protein 1 [Source:HGNC Symbol;Acc:18708]	0.83	0.3	0.89
KIAA0922	KIAA0922 [Source:HGNC Symbol;Acc:29146]	0.21	0.21	0.27
ALPL	alkaline phosphatase, liver/bone/kidney [Source:HGNC Symbol;Acc:438]	0.21	0.2	0.27
SYT3	synaptotagmin III [Source:HGNC Symbol;Acc:11511]	0.22	0.21	0.28
RBM15B	RNA binding motif protein 15B [Source:HGNC Symbol;Acc:24303]	0.21	0.19	0.27
MPRIP	myosin phosphatase Rho interacting protein [Source:HGNC Symbol;Acc:30321]	0.21	0.18	0.27
TRAM1L1	translocation associated membrane protein 1-like 1 [Source:HGNC Symbol;Acc:28371]	0.18	0.21	0.27
NKX3-1	NK3 homeobox 1 [Source:HGNC Symbol;Acc:7838]	0.08	0.11	0.17
SLCO2A1	solute carrier organic anion transporter family, member 2A1 [Source:HGNC Symbol;Acc:10955]	0.04	0.08	0.14
FAM196B	family with sequence similarity 196, member B [Source:HGNC Symbol;Acc:37271]	0.08	0.02	0.14
CCDC147	coiled-coil domain containing 147 [Source:HGNC Symbol;Acc:26676]	0.01	0.01	0.07
GDF6	growth differentiation factor 6 [Source:HGNC Symbol;Acc:4221]	0	0	0.06
DAAM2	dishevelled associated activator of morphogenesis 2 [Source:HGNC Symbol;Acc:18143]	0.45	0.45	0.51
FGF8	fibroblast growth factor 8 (androgen-induced) [Source:HGNC Symbol;Acc:3686]	0.02	0.02	0.08
CLPB	ClpB caseinolytic peptidase B homolog (E. coli) [Source:HGNC Symbol;Acc:30664]	0.33	0.33	0.39
KIAA1755	KIAA1755 [Source:HGNC Symbol;Acc:29372]	0	0	0.06
SNORD12B	small nucleolar RNA, C/D box 12B [Source:HGNC Symbol;Acc:33573]	0	0	0.06
HPGD	hydroxyprostaglandin dehydrogenase 15-(NAD) [Source:HGNC Symbol;Acc:5154]	0	0	0.06
C4orf33	chromosome 4 open reading frame 33 [Source:HGNC Symbol;Acc:27025]	0	0	0.06
SLC35B2	solute carrier family 35, member B2 [Source:HGNC Symbol;Acc:16872]	0.07	0.07	0.13
ACTL6B	actin-like 6B [Source:HGNC Symbol;Acc:160]	0.35	0.35	0.41
HKR1	HKR1, GLI-Kruppel zinc finger family member [Source:HGNC Symbol;Acc:4928]	0.17	0.18	0.24
KLHL32	kelch-like 32 (Drosophila) [Source:HGNC Symbol;Acc:21221]	0.23	0.24	0.3
TMCO4	transmembrane and coiled-coil domains 4 [Source:HGNC Symbol;Acc:27393]	0.06	0.05	0.12
LRRC3	leucine rich repeat containing 3 [Source:HGNC Symbol;Acc:14965]	0.06	0.05	0.12
GYPC	glycophorin C (Gerbich blood group) [Source:HGNC Symbol;Acc:4704]	0.02	0.01	0.08
CSNK1G3	casein kinase 1, gamma 3 [Source:HGNC Symbol;Acc:2456]	0.14	0.13	0.2
ENTPD3	ectonucleoside triphosphate diphosphohydrolase 3 [Source:HGNC Symbol;Acc:3365]	0.31	0.32	0.38
RLIM	ring finger protein, LIM domain interacting [Source:HGNC Symbol;Acc:13429]	0.37	0.38	0.44
CHODL	chondrolectin [Source:HGNC Symbol;Acc:17807]	0.24	0.22	0.3
RPL39L	ribosomal protein L39-like [Source:HGNC Symbol;Acc:17094]	0.06	0.04	0.12
STK32C	serine/threonine kinase 32C [Source:HGNC Symbol;Acc:21332]	0.13	0.11	0.19
ZEB2	zinc finger E-box binding homeobox 2 [Source:HGNC Symbol;Acc:14881]	0.03	0.01	0.09
MARK1	MAP/microtubule affinity-regulating kinase 1 [Source:HGNC Symbol;Acc:6896]	0.01	0.03	0.09
FAHD2A	fumarylacetoacetate hydrolase domain containing 2A [Source:HGNC Symbol;Acc:24252]	0.31	0.33	0.39
STX17	syntaxin 17 [Source:HGNC Symbol;Acc:11432]	0.12	0.14	0.2
LAS1L	LAS1-like (S. cerevisiae) [Source:HGNC Symbol;Acc:25726]	0.38	0.4	0.46
C11orf35	chromosome 11 open reading frame 35 [Source:HGNC Symbol;Acc:28561]	0.15	0.12	0.21
RASSF7	Ras association (RalGDS/AF-6) domain family (N-terminal) member 7 [Source:HGNC Symbol;Acc:1166]	0.15	0.12	0.21
SNORA26	Small nucleolar RNA SNORA26 [Source:RFAM;Acc:RF00568]	0.06	0.03	0.12
PTPRZ1	protein tyrosine phosphatase, receptor-type, Z polypeptide 1 [Source:HGNC Symbol;Acc:9685]	0.06	0.03	0.12
ABR	active BCR-related [Source:HGNC Symbol;Acc:81]	0.13	0.09	0.19

GRM1	glutamate receptor, metabotropic 1 [Source:HGNC Symbol;Acc:4593]	0.26	0.22	0.32
CCS	copper chaperone for superoxide dismutase [Source:HGNC Symbol;Acc:1613]	0.3	0.34	0.4
CXorf22	chromosome X open reading frame 22 [Source:HGNC Symbol;Acc:28546]	0.41	0.45	0.51
NKAIN1	Na+/K+ transporting ATPase interacting 1 [Source:HGNC Symbol;Acc:25743]	0.07	0.02	0.13
C1orf131	chromosome 1 open reading frame 131 [Source:HGNC Symbol;Acc:25332]	0.12	0.06	0.18
GNPAT	glyceronephosphate O-acyltransferase [Source:HGNC Symbol;Acc:4416]	0.12	0.06	0.18
DCHS1	dachsous 1 (Drosophila) [Source:HGNC Symbol;Acc:13681]	0.09	0.03	0.15
SUPT3H	suppressor of Ty 3 homolog (S. cerevisiae) [Source:HGNC Symbol;Acc:11466]	0.1	0.04	0.16
TGFB2	transforming growth factor, beta 2 [Source:HGNC Symbol;Acc:11768]	0.04	0.1	0.16
HOXA6	homeobox A6 [Source:HGNC Symbol;Acc:5107]	0.14	0.2	0.26
KIF12	kinesin family member 12 [Source:HGNC Symbol;Acc:21495]	0.11	0.17	0.23
RPL18A	Small nucleolar RNA SNORA68 [Source:RFAM;Acc:RF00263]	0.28	0.21	0.34
USH1G	Usher syndrome 1G (autosomal recessive) [Source:HGNC Symbol;Acc:16356]	0.25	0.17	0.31
MT1G	metallothionein 1G [Source:HGNC Symbol;Acc:7399]	0.11	0.19	0.25
LDB2	LIM domain binding 2 [Source:HGNC Symbol;Acc:6533]	0.25	0.34	0.4
VDAC2	voltage-dependent anion channel 2 [Source:HGNC Symbol;Acc:12672]	0.15	0.04	0.21
COL1A1	collagen, type I, alpha 1 [Source:HGNC Symbol;Acc:2197]	0.07	0.19	0.25
S100A10	S100 calcium binding protein A10 [Source:HGNC Symbol;Acc:10487]	0.01	0.17	0.23
PITPNM3	PITPNM family member 3 [Source:HGNC Symbol;Acc:21043]	0.15	0.38	0.44
HSF1	heat shock transcription factor 1 [Source:HGNC Symbol;Acc:5224]	0.32	0.06	0.38
BTBD17	BTB (POZ) domain containing 17 [Source:HGNC Symbol;Acc:33758]	0.39	0.12	0.45
PLCXD3	phosphatidylinositol-specific phospholipase C, X domain containing 3 [Source:HGNC Symbol;Acc:31822]	0.21	0.23	0.29
PKD1L1	polycystic kidney disease 1 like 1 [Source:HGNC Symbol;Acc:18053]	0.91	0.91	0.97
LCN1	lipocalin 1 [Source:HGNC Symbol;Acc:6525]	0.9	0.9	0.96
ZNF735	zinc finger protein 679 [Source:HGNC Symbol;Acc:28650]	0.9	0.89	0.96
LHX8	LIM homeobox 8 [Source:HGNC Symbol;Acc:28838]	0.89	0.9	0.96
AOC2	amine oxidase, copper containing 2 (retina-specific) [Source:HGNC Symbol;Acc:549]	0.91	0.92	0.98
TGM2	transglutaminase 2 (C polypeptide, protein-glutamine-gamma-glutamyltransferase) [Source:HGNC Symbol;Acc:11778]	0.88	0.86	0.94
NR1H4	nuclear receptor subfamily 1, group H, member 4 [Source:HGNC Symbol;Acc:7967]	0.88	0.86	0.94
ELANE	elastase, neutrophil expressed [Source:HGNC Symbol;Acc:3309]	0.76	0.74	0.82
IL24	interleukin 24 [Source:HGNC Symbol;Acc:11346]	0.9	0.92	0.98
WFDC9	WAP four-disulfide core domain 9 [Source:HGNC Symbol;Acc:20380]	0.9	0.87	0.96
DIXDC1	DIX domain containing 1 [Source:HGNC Symbol;Acc:23695]	0.76	0.79	0.85
BEST3	bestrophin 3 [Source:HGNC Symbol;Acc:17105]	0.88	0.91	0.97
MSMB	microseminoprotein, beta- [Source:HGNC Symbol;Acc:7372]	0.91	0.87	0.97
NFE2L3	nuclear factor (erythroid-derived 2)-like 3 [Source:HGNC Symbol;Acc:7783]	0.91	0.87	0.97
OR9A2	olfactory receptor, family 9, subfamily A, member 2 [Source:HGNC Symbol;Acc:15093]	0.93	0.89	0.99
TRPA1	transient receptor potential cation channel, subfamily A, member 1 [Source:HGNC Symbol;Acc:497]	0.61	0.65	0.71
C9orf24	chromosome 9 open reading frame 24 [Source:HGNC Symbol;Acc:19919]	0.76	0.8	0.86
CORO1A	coronin, actin binding protein, 1A [Source:HGNC Symbol;Acc:2252]	0.79	0.74	0.85
FBXO41	F-box protein 41 [Source:HGNC Symbol;Acc:29409]	0.91	0.86	0.97
ERG	v-ets erythroblastosis virus E26 oncogene homolog (avian) [Source:HGNC Symbol;Acc:3446]	0.73	0.78	0.84
TMEM194B	transmembrane protein 194B [Source:HGNC Symbol;Acc:33700]	0.82	0.88	0.94
ARHGDIB	Rho GDP dissociation inhibitor (GDI) beta [Source:HGNC Symbol;Acc:679]	0.7	0.77	0.83
RASAL3	RAS protein activator like 3 [Source:HGNC Symbol;Acc:26129]	0.78	0.71	0.84
KRT85	keratin 85 [Source:HGNC Symbol;Acc:6462]	0.89	0.81	0.95
APOF	apolipoprotein F [Source:HGNC Symbol;Acc:615]	0.9	0.82	0.96
C3orf18	chromosome 3 open reading frame 18 [Source:HGNC Symbol;Acc:24837]	0.71	0.79	0.85
C9orf152	chromosome 9 open reading frame 152 [Source:HGNC Symbol;Acc:31455]	0.88	0.78	0.94
CCBE1	collagen and calcium binding EGF domains 1 [Source:HGNC Symbol;Acc:29426]	0.41	0.51	0.57
FHL1	four and a half LIM domains 1 [Source:HGNC Symbol;Acc:3702]	0.81	0.71	0.87
THBS4	thrombospondin 4 [Source:HGNC Symbol;Acc:11788]	0.41	0.52	0.58
TBC1D8B	TBC1 domain family, member 8B (with GRAM domain) [Source:HGNC Symbol;Acc:24715]	0.68	0.56	0.74
GPR173	G protein-coupled receptor 173 [Source:HGNC Symbol;Acc:18186]	0.52	0.4	0.58
COL8A2	collagen, type VIII, alpha 2 [Source:HGNC Symbol;Acc:2216]	0.41	0.53	0.59
ITGA2B	integrin, alpha 2b (platelet glycoprotein IIb of IIb/IIIa complex, antigen CD41) [Source:HGNC Symbol;Acc:6138]	0.75	0.88	0.94
KIF3C	kinesin family member 3C [Source:HGNC Symbol;Acc:6321]	0.53	0.66	0.72
KNG1	kininogen 1 [Source:HGNC Symbol;Acc:6383]	0.56	0.43	0.62
UGT2B4	UDP glucuronosyltransferase 2 family, polypeptide B4 [Source:HGNC Symbol;Acc:12553]	0.92	0.75	0.98
DENND1C	DENN/MADD domain containing 1C [Source:HGNC Symbol;Acc:26225]	0.56	0.36	0.62
SYTL3	synaptotagmin-like 3 [Source:HGNC Symbol;Acc:15587]	0.67	0.45	0.73
ULBP3	UL16 binding protein 3 [Source:HGNC Symbol;Acc:14895]	0.77	0.53	0.83
ECHDC2	enoyl CoA hydratase domain containing 2 [Source:HGNC Symbol;Acc:23408]	0.51	0.78	0.84
FGG	fibrinogen gamma chain [Source:HGNC Symbol;Acc:3694]	0.92	0.63	0.98
FAM26E	family with sequence similarity 26, member E [Source:HGNC Symbol;Acc:21568]	0.79	0.45	0.85

CPZ	carboxypeptidase Z [Source:HGNC Symbol;Acc:2333]	0.67	0.33	0.73
ADAD2	adenosine deaminase domain containing 2 [Source:HGNC Symbol;Acc:30714]	0.87	0.87	0.92
SLC10A4	solute carrier family 10 (sodium/bile acid cotransporter family), member 4 [Source:HGNC Symbol;Acc:22980]	0.7	0.7	0.75
PRKG2	protein kinase, cGMP-dependent, type II [Source:HGNC Symbol;Acc:9416]	0.88	0.88	0.93
ADRB2	adrenoceptor beta 2, surface [Source:HGNC Symbol;Acc:286]	0.85	0.86	0.91
RPS6KA1	ribosomal protein S6 kinase, 90kDa, polypeptide 1 [Source:HGNC Symbol;Acc:10430]	0.87	0.86	0.92
BCAS1	breast carcinoma amplified sequence 1 [Source:HGNC Symbol;Acc:974]	0.95	0.94	1
KCTD6	potassium channel tetramerisation domain containing 6 [Source:HGNC Symbol;Acc:22235]	0.84	0.83	0.89
BTBD16	BTB (POZ) domain containing 16 [Source:HGNC Symbol;Acc:26340]	0.85	0.86	0.91
IFIT1B	interferon-induced protein with tetratricopeptide repeats 1B [Source:HGNC Symbol;Acc:23442]	0.75	0.76	0.81
TPD52	tumor protein D52 [Source:HGNC Symbol;Acc:12005]	0.7	0.71	0.76
TIGIT	T cell immunoreceptor with Ig and ITIM domains [Source:HGNC Symbol;Acc:26838]	0.84	0.82	0.89
AMOT	angiomotin [Source:HGNC Symbol;Acc:17810]	0.86	0.88	0.93
CALHM2	calcium homeostasis modulator 2 [Source:HGNC Symbol;Acc:23493]	0.7	0.67	0.75
ZNF713	zinc finger protein 713 [Source:HGNC Symbol;Acc:22043]	0.95	0.92	1
BAALC	brain and acute leukemia, cytoplasmic [Source:HGNC Symbol;Acc:14333]	0.81	0.84	0.89
GLA	galactosidase, alpha [Source:HGNC Symbol;Acc:4296]	0.49	0.46	0.54
HNRNPH2	heterogeneous nuclear ribonucleoprotein H2 (H') [Source:HGNC Symbol;Acc:5042]	0.49	0.46	0.54
PDZD4	PDZ domain containing 4 [Source:HGNC Symbol;Acc:21167]	0.69	0.72	0.77
SNCG	synuclein, gamma (breast cancer-specific protein 1) [Source:HGNC Symbol;Acc:11141]	0.85	0.81	0.9
STARD3NL	STARD3 N-terminal like [Source:HGNC Symbol;Acc:19169]	0.91	0.95	1
MMP12	matrix metallopeptidase 12 (macrophage elastase) [Source:HGNC Symbol;Acc:7158]	0.86	0.82	0.91
SULT1A1	sulfotransferase family, cytosolic, 1A, phenol-preferring, member 1 [Source:HGNC Symbol;Acc:11453]	0.83	0.78	0.88
TRIM38	tripartite motif containing 38 [Source:HGNC Symbol;Acc:10059]	0.85	0.8	0.9
TMC4	transmembrane channel-like 4 [Source:HGNC Symbol;Acc:22998]	0.54	0.59	0.64
PROM2	prominin 2 [Source:HGNC Symbol;Acc:20685]	0.66	0.71	0.76
RTP3	receptor (chemosensory) transporter protein 3 [Source:HGNC Symbol;Acc:15572]	0.74	0.69	0.79
RHD	Rh blood group, CcEe antigens [Source:HGNC Symbol;Acc:10008]	0.7	0.75	0.8
GPA33	glycoprotein A33 (transmembrane) [Source:HGNC Symbol;Acc:4445]	0.94	0.88	0.99
FAM186B	family with sequence similarity 186, member B [Source:HGNC Symbol;Acc:25296]	0.83	0.77	0.88
TTC22	tetratricopeptide repeat domain 22 [Source:HGNC Symbol;Acc:26067]	0.77	0.83	0.88
SPOCD1	SPOC domain containing 1 [Source:HGNC Symbol;Acc:26338]	0.51	0.57	0.62
PPP2R5B	protein phosphatase 2, regulatory subunit B', beta [Source:HGNC Symbol;Acc:9310]	0.41	0.35	0.46
KRTAP10-2	keratin associated protein 10-2 [Source:HGNC Symbol;Acc:22967]	0.73	0.66	0.78
C1orf189	chromosome 1 open reading frame 189 [Source:HGNC Symbol;Acc:32305]	0.87	0.79	0.92
TSPAN2	tetraspanin 2 [Source:HGNC Symbol;Acc:20659]	0.88	0.8	0.93
XAF1	XIAP associated factor 1 [Source:HGNC Symbol;Acc:30932]	0.84	0.76	0.89
GPT	glutamic-pyruvate transaminase (alanine aminotransferase) [Source:HGNC Symbol;Acc:4552]	0.85	0.77	0.9
NPHS2	nephrosis 2, idiopathic, steroid-resistant (podocin) [Source:HGNC Symbol;Acc:13394]	0.86	0.94	0.99
NUDT18	nudix (nucleoside diphosphate linked moiety X)-type motif 18 [Source:HGNC Symbol;Acc:26194]	0.55	0.63	0.68
C10orf11	chromosome 10 open reading frame 11 [Source:HGNC Symbol;Acc:23405]	0.84	0.75	0.89
SIPA1L1	signal-induced proliferation-associated 1 like 1 [Source:HGNC Symbol;Acc:20284]	0.95	0.86	1
C20orf202	chromosome 20 open reading frame 202 [Source:HGNC Symbol;Acc:37254]	0.82	0.73	0.87
OIT3	oncoprotein induced transcript 3 [Source:HGNC Symbol;Acc:29953]	0.86	0.95	1
ECSCR	endothelial cell surface expressed chemotaxis and apoptosis regulator [Source:HGNC Symbol;Acc:35454]	0.67	0.76	0.81
KIAA0513	KIAA0513 [Source:HGNC Symbol;Acc:29058]	0.95	0.85	1
TNFSF4	tumor necrosis factor (ligand) superfamily, member 4 [Source:HGNC Symbol;Acc:11934]	0.85	0.72	0.9
KRT71	keratin 71 [Source:HGNC Symbol;Acc:28927]	0.83	0.69	0.88
RNASE13	ribonuclease, RNase A family, 13 (non-active) [Source:HGNC Symbol;Acc:25285]	0.83	0.69	0.88
C5orf20	chromosome 5 open reading frame 20 [Source:HGNC Symbol;Acc:24459]	0.86	0.71	0.91
TAS1R2	taste receptor, type 1, member 2 [Source:HGNC Symbol;Acc:14905]	0.62	0.46	0.67
FCRL6	Fc receptor-like 6 [Source:HGNC Symbol;Acc:31910]	0.88	0.72	0.93
PGAM2	phosphoglycerate mutase 2 (muscle) [Source:HGNC Symbol;Acc:8889]	0.51	0.7	0.75
DNAH2	dynein, axonemal, heavy chain 2 [Source:HGNC Symbol;Acc:2948]	0.73	0.51	0.78
IKZF1	IKAROS family zinc finger 1 (Ikaros) [Source:HGNC Symbol;Acc:13176]	0.58	0.36	0.63
TMBIM1	transmembrane BAX inhibitor motif containing 1 [Source:HGNC Symbol;Acc:23410]	0.58	0.82	0.87
MORC2	MORC family CW-type zinc finger 2 [Source:HGNC Symbol;Acc:23573]	0.47	0.71	0.76

OR52J3	olfactory receptor, family 52, subfamily J, member 3 [Source:HGNC Symbol;Acc:14799]	0.87	0.6	0.92
LRPAP1	low density lipoprotein receptor-related protein associated protein 1 [Source:HGNC Symbol;Acc:6701]	0.48	0.12	0.53
LRRC23	leucine rich repeat containing 23 [Source:HGNC Symbol;Acc:19138]	0.48	0.08	0.53
C11orf88	chromosome 11 open reading frame 88 [Source:HGNC Symbol;Acc:25061]	0.14	0.15	0.2
HAND2	heart and neural crest derivatives expressed 2 [Source:HGNC Symbol;Acc:4808]	0.09	0.07	0.14
WDR63	WD repeat domain 63 [Source:HGNC Symbol;Acc:30711]	0.12	0.1	0.17
GLIPR1L1	GLI pathogenesis-related 1 like 1 [Source:HGNC Symbol;Acc:28392]	0.15	0.13	0.2
RFX5	regulatory factor X, 5 (influences HLA class II expression) [Source:HGNC Symbol;Acc:9986]	0.09	0.06	0.14
SPEF1	sperm flagellar 1 [Source:HGNC Symbol;Acc:15874]	0.15	0.12	0.2
TUBA8	tubulin, alpha 8 [Source:HGNC Symbol;Acc:12410]	0.05	0.09	0.14
PHYH	phytanoyl-CoA 2-hydroxylase [Source:HGNC Symbol;Acc:8940]	0.12	0.07	0.17
SLC25A42	solute carrier family 25, member 42 [Source:HGNC Symbol;Acc:28380]	0.21	0.15	0.26
NKX2-3	NK2 homeobox 3 [Source:HGNC Symbol;Acc:7836]	0.16	0.22	0.27
SH2B3	SH2B adaptor protein 3 [Source:HGNC Symbol;Acc:29605]	0.04	0.12	0.17
KSR2	kinase suppressor of ras 2 [Source:HGNC Symbol;Acc:18610]	0.12	0.21	0.26
RSAD1	radical S-adenosyl methionine domain containing 1 [Source:HGNC Symbol;Acc:25634]	0.12	0.02	0.17
LBX2	ladybird homeobox 2 [Source:HGNC Symbol;Acc:15525]	0.07	0.18	0.23
PRDM13	PR domain containing 13 [Source:HGNC Symbol;Acc:13998]	0.18	0.06	0.23
C8orf73	maestro heat-like repeat family member 6 [Source:HGNC Symbol;Acc:27814]	0.09	0.22	0.27
SALL4	sal-like 4 (Drosophila) [Source:HGNC Symbol;Acc:15924]	0.08	0.23	0.28
SHE	Src homology 2 domain containing E [Source:HGNC Symbol;Acc:27004]	0.05	0.05	0.1
TDRD10	tudor domain containing 10 [Source:HGNC Symbol;Acc:25316]	0.05	0.05	0.1
LCOR	ligand dependent nuclear receptor corepressor [Source:HGNC Symbol;Acc:29503]	0.03	0.03	0.08
HHEX	hematopoietically expressed homeobox [Source:HGNC Symbol;Acc:4901]	0.02	0.02	0.07
GDF10	growth differentiation factor 10 [Source:HGNC Symbol;Acc:4215]	0	0	0.05
DLX4	distal-less homeobox 4 [Source:HGNC Symbol;Acc:2917]	0.05	0.05	0.1
NEURL3	neuralized homolog 3 (Drosophila) pseudogene [Source:HGNC Symbol;Acc:25162]	0.03	0.03	0.08
EVX1	even-skipped homeobox 1 [Source:HGNC Symbol;Acc:3506]	0.02	0.02	0.07
DECR1	2,4-dienoyl CoA reductase 1, mitochondrial [Source:HGNC Symbol;Acc:2753]	0.02	0.02	0.07
FAM129A	family with sequence similarity 129, member A [Source:HGNC Symbol;Acc:16784]	0.11	0.1	0.16
ANK3	ankyrin 3, node of Ranvier (ankyrin G) [Source:HGNC Symbol;Acc:494]	0.03	0.02	0.08
APOA1BP	apolipoprotein A-I binding protein [Source:HGNC Symbol;Acc:18453]	0.05	0.04	0.1
GPR135	G protein-coupled receptor 135 [Source:HGNC Symbol;Acc:19991]	0.01	0.02	0.07
FZD5	frizzled family receptor 5 [Source:HGNC Symbol;Acc:4043]	0.01	0.02	0.07
PPARA	peroxisome proliferator-activated receptor alpha [Source:HGNC Symbol;Acc:9232]	0.03	0.01	0.08
PRDM8	PR domain containing 8 [Source:HGNC Symbol;Acc:13993]	0.03	0.01	0.08
EBF4	early B-cell factor 4 [Source:HGNC Symbol;Acc:29278]	0.09	0.11	0.16
HMOX1	heme oxygenase (decycling) 1 [Source:HGNC Symbol;Acc:5013]	0.04	0.06	0.11
PPP1CC	protein phosphatase 1, catalytic subunit, gamma isozyme [Source:HGNC Symbol;Acc:9283]	0.06	0.03	0.11
PRKCH	protein kinase C, eta [Source:HGNC Symbol;Acc:9403]	0.05	0.11	0.16
FIBP	fibroblast growth factor (acidic) intracellular binding protein [Source:HGNC Symbol;Acc:3705]	0.01	0.01	0.06
BICD1	bicaudal D homolog 1 (Drosophila) [Source:HGNC Symbol;Acc:1049]	0.04	0.04	0.09
SRI	sorcin [Source:HGNC Symbol;Acc:11292]	0.01	0.01	0.06
CCDC88C	coiled-coil domain containing 88C [Source:HGNC Symbol;Acc:19967]	0.04	0.03	0.09
RASSF2	Ras association (RalGDS/AF-6) domain family member 2 [Source:HGNC Symbol;Acc:9883]	0	0.01	0.06
RRAD	Ras-related associated with diabetes [Source:HGNC Symbol;Acc:10446]	0.17	0.17	0.22
OSBPL5	oxysterol binding protein-like 5 [Source:HGNC Symbol;Acc:16392]	0.31	0.3	0.36
DFNB59	deafness, autosomal recessive 59 [Source:HGNC Symbol;Acc:29502]	0.13	0.12	0.18
COL23A1	collagen, type XXIII, alpha 1 [Source:HGNC Symbol;Acc:22990]	0.25	0.24	0.3
ESD	esterase D [Source:HGNC Symbol;Acc:3465]	0.06	0.07	0.12
EXOC3L2	exocyst complex component 3-like 2 [Source:HGNC Symbol;Acc:30162]	0.15	0.16	0.21
YIPF6	Yip1 domain family, member 6 [Source:HGNC Symbol;Acc:28304]	0.37	0.38	0.43
EVC	Ellis van Creveld syndrome [Source:HGNC Symbol;Acc:3497]	0.14	0.16	0.21
SQLE	squalene epoxidase [Source:HGNC Symbol;Acc:11279]	0.17	0.19	0.24
TMEM128	transmembrane protein 128 [Source:HGNC Symbol;Acc:28201]	0.28	0.26	0.33
ALS2CL	ALS2 C-terminal like [Source:HGNC Symbol;Acc:20605]	0.44	0.46	0.51
HECW1	HECT, C2 and WW domain containing E3 ubiquitin protein ligase 1 [Source:HGNC Symbol;Acc:22195]	0.24	0.21	0.29
MAPRE3	microtubule-associated protein, RP/EB family, member 3 [Source:HGNC Symbol;Acc:6892]	0.04	0.07	0.12
CLIC6	chloride intracellular channel 6 [Source:HGNC Symbol;Acc:2065]	0.13	0.16	0.21
AVPR1B	arginine vasopressin receptor 1B [Source:HGNC Symbol;Acc:896]	0.43	0.46	0.51
RASA2	RAS p21 protein activator 2 [Source:HGNC Symbol;Acc:9872]	0.17	0.13	0.22
CCDC87	coiled-coil domain containing 87 [Source:HGNC Symbol;Acc:25579]	0.32	0.37	0.42

FAM150B	family with sequence similarity 150, member B [Source:HGNC Symbol;Acc:27683]	0.02	0.07	0.12
MTA3	metastasis associated 1 family, member 3 [Source:HGNC Symbol;Acc:23784]	0.18	0.24	0.29
TRIB2	tribbles homolog 2 (Drosophila) [Source:HGNC Symbol;Acc:30809]	0.13	0.19	0.24
DUSP19	dual specificity phosphatase 19 [Source:HGNC Symbol;Acc:18894]	0.27	0.21	0.32
LGR6	leucine-rich repeat containing G protein-coupled receptor 6 [Source:HGNC Symbol;Acc:19719]	0.31	0.24	0.36
WNT10B	wingless-type MMTV integration site family, member 10B [Source:HGNC Symbol;Acc:12775]	0.16	0.24	0.29
MBNL1	muscleblind-like splicing regulator 1 [Source:HGNC Symbol;Acc:6923]	0.46	0.38	0.51
L3MBTL4	l(3)mbt-like 4 (Drosophila) [Source:HGNC Symbol;Acc:26677]	0.15	0.24	0.29
MATN2	matrilin 2 [Source:HGNC Symbol;Acc:6908]	0.05	0.14	0.19
EFNB3	ephrin-B3 [Source:HGNC Symbol;Acc:3228]	0.04	0.14	0.19
FBP1	fructose-1,6-bisphosphatase 1 [Source:HGNC Symbol;Acc:3606]	0.04	0.14	0.19
TRIM45	tripartite motif containing 45 [Source:HGNC Symbol;Acc:19018]	0.16	0.05	0.21
HTR7	5-hydroxytryptamine (serotonin) receptor 7, adenylate cyclase-coupled [Source:HGNC Symbol;Acc:5302]	0.19	0.33	0.38
SUCLG1	succinate-CoA ligase, alpha subunit [Source:HGNC Symbol;Acc:11449]	0.24	0.09	0.29
IBTK	inhibitor of Bruton agammaglobulinemia tyrosine kinase [Source:HGNC Symbol;Acc:17853]	0.25	0.1	0.3
EHHADH	enoyl-CoA, hydratase/3-hydroxyacyl CoA dehydrogenase [Source:HGNC Symbol;Acc:3247]	0.23	0.39	0.44
ABHD6	abhydrolase domain containing 6 [Source:HGNC Symbol;Acc:21398]	0.28	0.45	0.5
HSD17B8	hydroxysteroid (17-beta) dehydrogenase 8 [Source:HGNC Symbol;Acc:3554]	0.14	0.36	0.41
AFF3	AF4/FMR2 family, member 3 [Source:HGNC Symbol;Acc:6473]	0.13	0.38	0.43
TNNT3	troponin T type 3 (skeletal, fast) [Source:HGNC Symbol;Acc:11950]	0.89	0.89	0.94
REC8	REC8 homolog (yeast) [Source:HGNC Symbol;Acc:16879]	0.92	0.92	0.97
SAP30BP	SAP30 binding protein [Source:HGNC Symbol;Acc:30785]	0.78	0.78	0.83
RECQL5	RecQ protein-like 5 [Source:HGNC Symbol;Acc:9950]	0.79	0.79	0.84
ANKRD23	ankyrin repeat domain 23 [Source:HGNC Symbol;Acc:24470]	0.92	0.92	0.97
IL1A	interleukin 1, alpha [Source:HGNC Symbol;Acc:5991]	0.68	0.68	0.73
MUC4	mucin 4, cell surface associated [Source:HGNC Symbol;Acc:7514]	0.92	0.92	0.97
SLC36A3	solute carrier family 36 (proton/amino acid symporter), member 3 [Source:HGNC Symbol;Acc:19659]	0.92	0.92	0.97
OR14J1	olfactory receptor, family 14, subfamily J, member 1 [Source:HGNC Symbol;Acc:13971]	0.9	0.9	0.95
HEPHL1	hephaestin-like 1 [Source:HGNC Symbol;Acc:30477]	0.8	0.79	0.85
FUT8	fucosyltransferase 8 (alpha (1,6) fucosyltransferase) [Source:HGNC Symbol;Acc:4019]	0.9	0.89	0.95
CD300C	CD300c molecule [Source:HGNC Symbol;Acc:19320]	0.89	0.88	0.94
SGK494	uncharacterized serine/threonine-protein kinase SgK494 [Source:RefSeq peptide;Acc:NP_001167574]	0.9	0.89	0.95
CDH26	cadherin 26 [Source:HGNC Symbol;Acc:15902]	0.93	0.92	0.98
LTA	lymphotoxin alpha (TNF superfamily, member 1) [Source:HGNC Symbol;Acc:6709]	0.91	0.9	0.96
CYP3A5	cytochrome P450, family 3, subfamily A, polypeptide 5 [Source:HGNC Symbol;Acc:2638]	0.92	0.91	0.97
KCNN4	potassium intermediate/small conductance calcium-activated channel, subfamily N, member 4 [Source:HGNC Symbol;Acc:6293]	0.88	0.89	0.94
GNLY	granulysin [Source:HGNC Symbol;Acc:4414]	0.92	0.93	0.98
TTPAL	tocopherol (alpha) transfer protein-like [Source:HGNC Symbol;Acc:16114]	0.64	0.65	0.7
ZNF883	zinc finger protein 883 [Source:HGNC Symbol;Acc:27271]	0.67	0.68	0.73
ESRRB	estrogen-related receptor beta [Source:HGNC Symbol;Acc:3473]	0.9	0.88	0.95
EXOSC9	exosome component 9 [Source:HGNC Symbol;Acc:9137]	0.66	0.64	0.71
TDO2	tryptophan 2,3-dioxygenase [Source:HGNC Symbol;Acc:11708]	0.9	0.88	0.95
SLC6A11	solute carrier family 6 (neurotransmitter transporter, GABA), member 11 [Source:HGNC Symbol;Acc:11044]	0.87	0.89	0.94
GSTA3	glutathione S-transferase alpha 3 [Source:HGNC Symbol;Acc:4628]	0.87	0.89	0.94
NYNRIN	NYN domain and retroviral integrase containing [Source:HGNC Symbol;Acc:20165]	0.8	0.77	0.85
MAGEA5	melanoma antigen family A, 5 [Source:HGNC Symbol;Acc:6803]	0.93	0.9	0.98
ABCA12	ATP-binding cassette, sub-family A (ABC1), member 12 [Source:HGNC Symbol;Acc:14637]	0.87	0.9	0.95
LRCH1	leucine-rich repeats and calponin homology (CH) domain containing 1 [Source:HGNC Symbol;Acc:20309]	0.76	0.8	0.85
GLT1D1	glycosyltransferase 1 domain containing 1 [Source:HGNC Symbol;Acc:26483]	0.86	0.9	0.95
SPDEF	SAM pointed domain containing ets transcription factor [Source:HGNC Symbol;Acc:17257]	0.91	0.86	0.96
TMEM176A	transmembrane protein 176A [Source:HGNC Symbol;Acc:24930]	0.77	0.72	0.82
GLP2R	glucagon-like peptide 2 receptor [Source:HGNC Symbol;Acc:4325]	0.75	0.8	0.85
USP51	ubiquitin specific peptidase 51 [Source:HGNC Symbol;Acc:23086]	0.5	0.55	0.6
DHRS9	dehydrogenase/reductase (SDR family) member 9 [Source:HGNC Symbol;Acc:16888]	0.79	0.73	0.84
PTPN7	protein tyrosine phosphatase, non-receptor type 7 [Source:HGNC Symbol;Acc:9659]	0.92	0.86	0.97

ATP6V0A4	ATPase, H+ transporting, lysosomal V0 subunit a4 [Source:HGNC Symbol;Acc:866]	0.79	0.73	0.84
TMEM213	transmembrane protein 213 [Source:HGNC Symbol;Acc:27220]	0.79	0.73	0.84
GOT1L1	glutamic-oxaloacetic transaminase 1-like 1 [Source:HGNC Symbol;Acc:28487]	0.92	0.86	0.97
OTUD5	OTU domain containing 5 [Source:HGNC Symbol;Acc:25402]	0.89	0.83	0.94
OR6V1	olfactory receptor, family 6, subfamily V, member 1 [Source:HGNC Symbol;Acc:15090]	0.89	0.82	0.94
EPS15	epidermal growth factor receptor pathway substrate 15 [Source:HGNC Symbol;Acc:3419]	0.57	0.64	0.69
TFF2	trefoil factor 2 [Source:HGNC Symbol;Acc:11756]	0.93	0.85	0.98
OR5AS1	olfactory receptor, family 5, subfamily AS, member 1 [Source:HGNC Symbol;Acc:15261]	0.9	0.81	0.95
KCNJ10	potassium inwardly-rectifying channel, subfamily J, member 10 [Source:HGNC Symbol;Acc:6256]	0.92	0.83	0.97
C1QTNF8	C1q and tumor necrosis factor related protein 8 [Source:HGNC Symbol;Acc:31374]	0.9	0.8	0.95
OR10K1	olfactory receptor, family 10, subfamily K, member 1 [Source:HGNC Symbol;Acc:14693]	0.93	0.83	0.98
PTGER1	prostaglandin E receptor 1 (subtype EP1), 42kDa [Source:HGNC Symbol;Acc:9593]	0.77	0.66	0.82
CTSE	cathepsin E [Source:HGNC Symbol;Acc:2530]	0.93	0.82	0.98
CYP2B6	cytochrome P450, family 2, subfamily B, polypeptide 6 [Source:HGNC Symbol;Acc:2615]	0.92	0.8	0.97
CEACAM19	carcinoembryonic antigen-related cell adhesion molecule 19 [Source:HGNC Symbol;Acc:31951]	0.68	0.56	0.73
BTN3A3	butyrophilin, subfamily 3, member A3 [Source:HGNC Symbol;Acc:1140]	0.77	0.63	0.82
RXRB	retinoid X receptor, beta [Source:HGNC Symbol;Acc:10478]	0.68	0.54	0.73
ZSCAN1	zinc finger and SCAN domain containing 1 [Source:HGNC Symbol;Acc:23712]	0.53	0.67	0.72
PGLYRP4	peptidoglycan recognition protein 4 [Source:HGNC Symbol;Acc:30015]	0.8	0.65	0.85
ITGAX	integrin, alpha X (complement component 3 receptor 4 subunit) [Source:HGNC Symbol;Acc:6152]	0.89	0.72	0.94
EPHX4	epoxide hydrolase 4 [Source:HGNC Symbol;Acc:23758]	0.63	0.8	0.85
TPI1	triosephosphate isomerase 1 [Source:HGNC Symbol;Acc:12009]	0.4	0.66	0.71
C16orf3	chromosome 16 open reading frame 3 [Source:HGNC Symbol;Acc:1197]	0.67	0.38	0.72
DRP2	dystrophin related protein 2 [Source:HGNC Symbol;Acc:3032]	0.77	0.44	0.82
TUB	tubby homolog (mouse) [Source:HGNC Symbol;Acc:12406]	0.8	0.28	0.85
