# Supplementary material for: Global DNA methylation and transcriptional analyses of human ESC-derived cardiomyocytes
Source: Protein Cell. 2014 Jan 29;5(1):59–68. doi: 10.1007/s13238-013-0016-x (PMC3938846; doi:10.1007/s13238-013-0016-x)
Supplement: Supplementary file 11 — Table S8: GO analysis of hCM differentially down-regulated genes-Cellular Component. This file contains the list of GO terms in the category of cellular component that were significantly (p<0.05) overrepresented among the 401 hCM differentiallydown-regulated genes [file 13238_2013_16_MOESM11_ESM.pdf]

Gene Name	Description and ID	DNA methylation		
		ES-mCpG	NSC-mCpG	CM-mCpG
POPDC2	popeye domain containing 2 [Source:HGNC Symbol;Acc:17648]	0.93	0.94	0.53
ALPK2	alpha-kinase 2 [Source:HGNC Symbol;Acc:20565]	0.78	0.59	0.25
RHOBTB2	Rho-related BTB domain containing 2 [Source:HGNC Symbol;Acc:18756]	0.96	0.72	0.38
ATP1A4	ATPase, Na+/K+ transporting, alpha 4 polypeptide [Source:HGNC Symbol;Acc:14073]	0.84	0.89	0.5
TRIM55	tripartite motif containing 55 [Source:HGNC Symbol;Acc:14215]	0.89	0.67	0.34
SPINK4	serine peptidase inhibitor, Kazal type 4 [Source:HGNC Symbol;Acc:16646]	0.94	0.87	0.56
ASPH	aspartate beta-hydroxylase [Source:HGNC Symbol;Acc:757]	0.83	0.93	0.53
LOC100287036	uncharacterized	0.91	0.53	0.26
AKR1B10	aldo-keto reductase family 1, member B10 (aldose reductase) [Source:HGNC Symbol;Acc:382]	0.72	0.56	0.33
SAMD9	sterile alpha motif domain containing 9 [Source:HGNC Symbol;Acc:1348]	0.81	0.73	0.5
ECE2	endothelin converting enzyme 2 [Source:HGNC Symbol;Acc:13275]	0.92	0.95	0.71
DMBT1	deleted in malignant brain tumors 1 [Source:HGNC Symbol;Acc:2926]	0.88	0.69	0.48
HSPB7	heat shock 27kDa protein family, member 7 (cardiovascular) [Source:HGNC Symbol;Acc:5249]	0.91	0.74	0.53
FILIP1	filamin A interacting protein 1 [Source:HGNC Symbol;Acc:21015]	0.91	0.75	0.54
GBP1	guanylate binding protein 1, interferon-inducible [Source:HGNC Symbol;Acc:4182]	0.88	0.91	0.68
NPPA	natriuretic peptide A [Source:HGNC Symbol;Acc:7939]	0.93	0.91	0.73
KBTBD10	kelch-like 41 (Drosophila) [Source:HGNC Symbol;Acc:16905]	0.9	0.91	0.72
ACOT11	acyl-CoA thioesterase 11 [Source:HGNC Symbol;Acc:18156]	0.86	0.78	0.6
C2orf71	chromosome 2 open reading frame 71 [Source:HGNC Symbol;Acc:34383]	0.93	0.91	0.73
OR4C3	olfactory receptor, family 4, subfamily C, member 3 [Source:HGNC Symbol;Acc:14697]	0.43	0.37	0.19
P2RY14	purinergic receptor P2Y, G-protein coupled, 14 [Source:HGNC Symbol;Acc:16442]	0.94	0.84	0.66
BRD4	bromodomain containing 4 [Source:HGNC Symbol;Acc:13575]	0.92	0.86	0.68
CLIC4	chloride intracellular channel 4 [Source:HGNC Symbol;Acc:13518]	0.86	0.86	0.68
MYL4	myosin, light chain 4, alkali; atrial, embryonic [Source:HGNC Symbol;Acc:7585]	0.92	0.81	0.64
TMEM223	transmembrane protein 223 [Source:HGNC Symbol;Acc:28464]	0.82	0.61	0.44
CRYAB	crystallin, alpha B [Source:HGNC Symbol;Acc:2389]	0.94	0.85	0.68
VIT	vitrin [Source:HGNC Symbol;Acc:12697]	0.87	0.7	0.53
NR1I2	nuclear receptor subfamily 1, group I, member 2 [Source:HGNC Symbol;Acc:7968]	0.91	1	0.75
SNORA20	Small nucleolar RNA SNORA20 [Source:RFAM;Acc:RF00401]	1	1	0.84
CCL19	chemokine (C-C motif) ligand 19 [Source:HGNC Symbol;Acc:10617]	0.91	0.92	0.75
LRRN4	leucine rich repeat neuronal 4 [Source:HGNC Symbol;Acc:16208]	0.68	0.71	0.53
C10orf71	chromosome 10 open reading frame 71 [Source:HGNC Symbol;Acc:26973]	0.84	0.64	0.49
IL9	interleukin 9 [Source:HGNC Symbol;Acc:6029]	0.93	0.89	0.74
POF1B	premature ovarian failure, 1B [Source:HGNC Symbol;Acc:13711]	0.74	0.7	0.55
SNORA46	Small nucleolar RNA SNORA46 [Source:RFAM;Acc:RF00404]	0.82	0.85	0.67
SOD3	superoxide dismutase 3, extracellular [Source:HGNC Symbol;Acc:11181]	0.83	0.84	0.68
MYBPHL	myosin binding protein H-like [Source:HGNC Symbol;Acc:30434]	0.75	0.33	0.19
TMEM72	transmembrane protein 72 [Source:HGNC Symbol;Acc:31658]	0.64	0.7	0.5
HRG	histidine-rich glycoprotein [Source:HGNC Symbol;Acc:5181]	1	1	0.86
CRYGS	crystallin, gamma S [Source:HGNC Symbol;Acc:2417]	0.96	1	0.82
C3orf43	chromosome 3 open reading frame 43 [Source:HGNC Symbol;Acc:27407]	0.87	0.91	0.73
CXorf64	chromosome X open reading frame 64 [Source:HGNC Symbol;Acc:34498]	0.76	0.8	0.62
NCCRP1	non-specific cytotoxic cell receptor protein 1 homolog (zebrafish) [Source:HGNC Symbol;Acc:33739]	0.37	0.18	0.04
MYOZ2	myozenin 2 [Source:HGNC Symbol;Acc:1330]	0.97	0.96	0.83
OBSCN	obscurin, cytoskeletal calmodulin and titin-interacting RhoGEF [Source:HGNC Symbol;Acc:15719]	0.9	0.86	0.73
KLHL30	kelch-like 30 (Drosophila) [Source:HGNC Symbol;Acc:24770]	0.94	0.84	0.71
LOC440563	heterogeneous nuclear ribonucleoprotein C-like 1 [Source:HGNC Symbol;Acc:29295]	0.92	0.87	0.74
FLJ43860	uncharacterized protein LOC389690 [Source:RefSeq peptide;Acc:NP_997297]	0.88	0.84	0.71
RNF43	ring finger protein 43 [Source:HGNC Symbol;Acc:18505]	0.29	0.25	0.12
KNCN	kinocilin [Source:HGNC Symbol;Acc:26488]	0.88	0.87	0.74
SEMA4A	sema domain, immunoglobulin domain (Ig), transmembrane domain (TM) and short cytoplasmic domain, (semaphorin) 4A [Source:HGNC Symbol;Acc:10729]	0.89	0.88	0.75
MPG	N-methylpurine-DNA glycosylase [Source:HGNC Symbol;Acc:7211]	0.98	1	0.85
CGA	glycoprotein hormones, alpha polypeptide [Source:HGNC Symbol;Acc:1885]	0.91	0.92	0.78
ACSL4	acyl-CoA synthetase long-chain family member 4 [Source:HGNC Symbol;Acc:3571]	0.6	0.6	0.47
COL4A5	collagen, type IV, alpha 5 [Source:HGNC Symbol;Acc:2207]	0.33	0.36	0.21
COL4A6	collagen, type IV, alpha 6 [Source:HGNC Symbol;Acc:2208]	0.33	0.36	0.21
FBXL20	F-box and leucine-rich repeat protein 20 [Source:HGNC Symbol;Acc:24679]	0.2	0.17	0.05
SMPX	small muscle protein, X-linked [Source:HGNC Symbol;Acc:11122]	0.92	0.85	0.73
ELF3	E74-like factor 3 (ets domain transcription factor, epithelial-specific) [Source:HGNC Symbol;Acc:3318]	0.74	0.85	0.62

MURC	muscle-related coiled-coil protein [Source:HGNC Symbol;Acc:33742]	0.93	0.97	0.81
PNPLA4	patatin-like phospholipase domain containing 4 [Source:HGNC Symbol;Acc:24887]	0.46	0.6	0.35
NDST1	N-deacetylase/N-sulfotransferase (heparan glucosaminyl) 1 [Source:HGNC Symbol;Acc:7680]	1	0.98	0.87
MAGEB18	melanoma antigen family B, 18 [Source:HGNC Symbol;Acc:28515]	0.78	0.76	0.65
MXRA8	matrix-remodelling associated 8 [Source:HGNC Symbol;Acc:7542]	0.92	0.95	0.81
CLCA1	chloride channel accessory 1 [Source:HGNC Symbol;Acc:2015]	1	1	0.89
MED6	mediator complex subunit 6 [Source:HGNC Symbol;Acc:19970]	0.84	0.91	0.73
SLC44A4	solute carrier family 44, member 4 [Source:HGNC Symbol;Acc:13941]	0.98	0.92	0.82
TRPM3	transient receptor potential cation channel, subfamily M, member 3 [Source:HGNC Symbol;Acc:17992]	0.92	0.92	0.82
NRK	Nik related kinase [Source:HGNC Symbol;Acc:25391]	0.48	0.51	0.38
ABLIM1	actin binding LIM protein 1 [Source:HGNC Symbol;Acc:78]	0.83	0.83	0.73
MAOA	monoamine oxidase A [Source:HGNC Symbol;Acc:6833]	0.55	0.42	0.32
C14orf180	chromosome 14 open reading frame 180 [Source:HGNC Symbol;Acc:33795]	0.92	0.84	0.74
PHEX	phosphate regulating endopeptidase homolog, X-linked [Source:HGNC Symbol;Acc:8918]	0.83	0.75	0.65
OR4C45	olfactory receptor, family 4, subfamily C, member 45 [Source:HGNC Symbol;Acc:31270]	0.76	0.7	0.6
CLEC4E	C-type lectin domain family 4, member E [Source:HGNC Symbol;Acc:14555]	1	1	0.9
PNPO	pyridoxamine 5'-phosphate oxidase [Source:HGNC Symbol;Acc:30260]	0.42	0.47	0.32
OR2H1	olfactory receptor, family 2, subfamily H, member 1 [Source:HGNC Symbol;Acc:8252]	0.95	0.93	0.84
IFNK	interferon, kappa [Source:HGNC Symbol;Acc:21714]	0.94	0.93	0.84
HSP90AB2P	heat shock protein 90kDa alpha (cytosolic), class B member 2, pseudogene [Source:HGNC Symbol;Acc:32537]	0.93	0.95	0.84
FAM26D	family with sequence similarity 26, member D [Source:HGNC Symbol;Acc:21094]	0.91	0.92	0.82
ACSF2	acyl-CoA synthetase family member 2 [Source:HGNC Symbol;Acc:26101]	0.7	0.73	0.61
INSC	inscuteable homolog (Drosophila) [Source:HGNC Symbol;Acc:33116]	0.9	0.71	0.62
KLK12	kallikrein-related peptidase 12 [Source:HGNC Symbol;Acc:6360]	0.67	0.61	0.52
UTS2	urotensin 2 [Source:HGNC Symbol;Acc:12636]	0.93	0.9	0.81
UGT2B7	UDP glucuronosyltransferase 2 family, polypeptide B7 [Source:HGNC Symbol;Acc:12554]	0.76	0.75	0.66
MPZ	myelin protein zero [Source:HGNC Symbol;Acc:7225]	0.86	0.94	0.77
ATP1A2	ATPase, Na+/K+ transporting, alpha 2 polypeptide [Source:HGNC Symbol;Acc:800]	0.59	0.59	0.5
RPS6KA4	ribosomal protein S6 kinase, 90kDa, polypeptide 4 [Source:HGNC Symbol;Acc:10433]	0.36	0.38	0.27
KIAA1671	KIAA1671 [Source:HGNC Symbol;Acc:29345]	0.82	0.83	0.73
SYNPO2L	synaptopodin 2-like [Source:HGNC Symbol;Acc:23532]	0.9	0.93	0.82
GJD4	GJD4 protein [Source:UniProtKB/TrEMBL;Acc:Q8IV12]	0.89	0.81	0.73
IL21	interleukin 21 [Source:HGNC Symbol;Acc:6005]	0.95	0.9	0.82
KCNAB1	potassium voltage-gated channel, shaker-related subfamily, beta member 1 [Source:HGNC Symbol;Acc:6228]	0.95	0.93	0.85
C1orf106	chromosome 1 open reading frame 106 [Source:HGNC Symbol;Acc:25599]	0.78	0.81	0.7
TBXAS1	thromboxane A synthase 1 (platelet) [Source:HGNC Symbol;Acc:11609]	0.91	0.92	0.83
APOBEC3G	apolipoprotein B mRNA editing enzyme, catalytic polypeptide-like 3G [Source:HGNC Symbol;Acc:17357]	0.34	0.36	0.26
ANKRD1	ankyrin repeat domain 1 (cardiac muscle) [Source:HGNC Symbol;Acc:15819]	0.25	0.18	0.1
TNNI1	troponin I type 1 (skeletal, slow) [Source:HGNC Symbol;Acc:11945]	0.79	0.7	0.62
C10orf10	chromosome 10 open reading frame 10 [Source:HGNC Symbol;Acc:23355]	0.83	0.86	0.75
HSPB2	HSPB2-C11orf52 readthrough (non-protein coding) [Source:HGNC Symbol;Acc:41996]	0.86	0.76	0.68
CSF1R	colony stimulating factor 1 receptor [Source:HGNC Symbol;Acc:2433]	0.78	0.74	0.66
TMPRSS13	transmembrane protease, serine 13 [Source:HGNC Symbol;Acc:29808]	0.75	0.74	0.66
FLJ23867	uncharacterized	0.75	0.75	0.67
BSCL2	Berardinelli-Seip congenital lipodystrophy 2 (seipin) [Source:HGNC Symbol;Acc:15832]	0.88	0.97	0.8
GNG3	guanine nucleotide binding protein (G protein), gamma 3 [Source:HGNC Symbol;Acc:4405]	0.88	0.97	0.8
KRTAP1-5	keratin associated protein 1-5 [Source:HGNC Symbol;Acc:16777]	0.85	0.88	0.77
MYH6	myosin, heavy chain 6, cardiac muscle, alpha [Source:HGNC Symbol;Acc:7576]	0.87	0.76	0.69
ABRA	actin-binding Rho activating protein [Source:HGNC Symbol;Acc:30655]	0.97	0.92	0.85
TPM1	tropomyosin 1 (alpha) [Source:HGNC Symbol;Acc:12010]	0.77	0.88	0.7
ZNF793	zinc finger protein 793 [Source:HGNC Symbol;Acc:33115]	0.82	0.76	0.69
OR5D16	olfactory receptor, family 5, subfamily D, member 16 [Source:HGNC Symbol;Acc:15283]	0.97	0.93	0.86
AIPL1	aryl hydrocarbon receptor interacting protein-like 1 [Source:HGNC Symbol;Acc:359]	0.71	0.67	0.6
PLEKHG7	pleckstrin homology domain containing, family G (with RhoGef domain) member 7 [Source:HGNC Symbol;Acc:33829]	0.94	0.91	0.84
CRYGC	crystallin, gamma C [Source:HGNC Symbol;Acc:2410]	0.93	0.92	0.85

ABCA4	ATP-binding cassette, sub-family A (ABC1), member 4 [Source:HGNC Symbol;Acc:34]	0.9	0.91	0.83
PF4V1	platelet factor 4 variant 1 [Source:HGNC Symbol;Acc:8862]	0.68	0.78	0.61
GALNT5	UDP-N-acetyl-alpha-D-galactosamine:polypeptide N-acetylgalactosaminyltransferase 5 (GalNAc-T5) [Source:HGNC Symbol;Acc:4127]	0.32	0.27	0.2
SCAF1	SR-related CTD-associated factor 1 [Source:HGNC Symbol;Acc:30403]	0.88	0.4	0.33
MAGEL2	MAGE-like 2 [Source:HGNC Symbol;Acc:6814]	0.57	0.45	0.38
CASK	calcium/calmodulin-dependent serine protein kinase (MAGUK family) [Source:HGNC Symbol;Acc:1497]	0.56	0.49	0.42
L3MBTL1	l(3)mbt-like 1 (Drosophila) [Source:HGNC Symbol;Acc:15905]	0.43	0.39	0.32
NPHP1	nephronophthisis 1 (juvenile) [Source:HGNC Symbol;Acc:7905]	0.53	0.51	0.44
DNAJC16	DnaJ (Hsp40) homolog, subfamily C, member 16 [Source:HGNC Symbol;Acc:29157]	0.42	0.52	0.35
TEAD4	TEA domain family member 4 [Source:HGNC Symbol;Acc:11717]	0.08	0.1	0.01
TSPAN14	tetraspanin 14 [Source:HGNC Symbol;Acc:23303]	0.18	0.27	0.11
RAB20	RAB20, member RAS oncogene family [Source:HGNC Symbol;Acc:18260]	0.21	0.21	0.14
SPP1	secreted phosphoprotein 1 [Source:HGNC Symbol;Acc:11255]	0.84	0.88	0.77
SLC10A3	solute carrier family 10 (sodium/bile acid cotransporter family), member 3 [Source:HGNC Symbol;Acc:22979]	0.83	0.71	0.64
BBOX1	butyrobetaine (gamma), 2-oxoglutarate dioxygenase (gamma-butyrobetaine hydroxylase) 1 [Source:HGNC Symbol;Acc:964]	0.92	0.82	0.75
SPANXN5	SPANX family, member N5 [Source:HGNC Symbol;Acc:33178]	0.71	0.63	0.56
DAPL1	death associated protein-like 1 [Source:HGNC Symbol;Acc:21490]	0.48	0.41	0.34
CFHR4	complement factor H-related 4 [Source:HGNC Symbol;Acc:16979]	0.96	0.95	0.88
HNF1A	HNF1 homeobox A [Source:HGNC Symbol;Acc:11621]	0.99	0.98	0.91
CLCA2	chloride channel accessory 2 [Source:HGNC Symbol;Acc:2016]	1	1	0.93
SCGB2A1	secretoglobin, family 2A, member 1 [Source:HGNC Symbol;Acc:7051]	0.96	1	0.89
C15orf52	chromosome 15 open reading frame 52 [Source:HGNC Symbol;Acc:33488]	0.87	0.91	0.8
MIA	melanoma inhibitory activity [Source:HGNC Symbol;Acc:7076]	0.87	0.97	0.8
CECR2	cat eye syndrome chromosome region, candidate 2 [Source:HGNC Symbol;Acc:1840]	0.95	0.96	0.88
MYOM1	myomesin 1 [Source:HGNC Symbol;Acc:7613]	0.92	0.9	0.84
RAB15	RAB15, member RAS oncogene family [Source:HGNC Symbol;Acc:20150]	0.67	0.71	0.61
MYOZ3	myozenin 3 [Source:HGNC Symbol;Acc:18565]	0.73	0.64	0.58
ANXA9	annexin A9 [Source:HGNC Symbol;Acc:547]	0.82	0.76	0.7
HMHA1	histocompatibility (minor) HA-1 [Source:HGNC Symbol;Acc:17102]	0.73	0.67	0.61
ZNF366	zinc finger protein 366 [Source:HGNC Symbol;Acc:18316]	0.92	0.9	0.84
FAM65C	family with sequence similarity 65, member C [Source:HGNC Symbol;Acc:16168]	0.58	0.56	0.5
S100A3	S100 calcium binding protein A3 [Source:HGNC Symbol;Acc:10493]	0.9	0.91	0.84
PADI1	peptidyl arginine deiminase, type I [Source:HGNC Symbol;Acc:18367]	0.91	0.91	0.85
NFASC	neurofascin [Source:HGNC Symbol;Acc:29866]	0.92	0.98	0.86
HTR3B	5-hydroxytryptamine (serotonin) receptor 3B, ionotropic [Source:HGNC Symbol;Acc:5298]	0.68	0.73	0.62
GRM5	glutamate receptor, metabotropic 5 [Source:HGNC Symbol;Acc:4597]	0.63	0.92	0.57
CD69	CD69 molecule [Source:HGNC Symbol;Acc:1694]	1	1	0.94
DAPP1	dual adaptor of phosphotyrosine and 3-phosphoinositides [Source:HGNC Symbol;Acc:16500]	0.93	0.97	0.87
RAB7L1	RAB7, member RAS oncogene family-like 1 [Source:HGNC Symbol;Acc:9789]	0.14	0.18	0.08
IFIT1	interferon-induced protein with tetratricopeptide repeats 1 [Source:HGNC Symbol;Acc:5407]	0.49	0.44	0.38
PALLD	palladin, cytoskeletal associated protein [Source:HGNC Symbol;Acc:17068]	0.54	0.32	0.26
TMEM120B	transmembrane protein 120B [Source:HGNC Symbol;Acc:32008]	0.31	0.36	0.25
RNASE6	ribonuclease, RNase A family, k6 [Source:HGNC Symbol;Acc:10048]	0.49	0.38	0.32
CHIC1	cysteine-rich hydrophobic domain 1 [Source:HGNC Symbol;Acc:1934]	0.4	0.31	0.25
SUV39H1	suppressor of variegation 3-9 homolog 1 (Drosophila) [Source:HGNC Symbol;Acc:11479]	0.49	0.43	0.37
MASP1	mannan-binding lectin serine peptidase 1 (C4/C2 activating component of Ra-reactive factor) [Source:HGNC Symbol;Acc:6901]	0.54	0.49	0.43
FABP7	fatty acid binding protein 7, brain [Source:HGNC Symbol;Acc:3562]	0.47	0.43	0.37
FAM63A	family with sequence similarity 63, member A [Source:HGNC Symbol;Acc:25648]	0.37	0.39	0.31
SUDS3	suppressor of defective silencing 3 homolog (S. cerevisiae) [Source:HGNC Symbol;Acc:29545]	0.33	0.33	0.27
UTS2D	urotensin 2 domain containing [Source:HGNC Symbol;Acc:30894]	0.44	0.46	0.38
TTK	TTK protein kinase [Source:HGNC Symbol;Acc:12401]	0.29	0.31	0.23
MYBPC3	myosin binding protein C, cardiac [Source:HGNC Symbol;Acc:7551]	0.89	0.83	0.77
APLNR	apelin receptor [Source:HGNC Symbol;Acc:339]	0.96	0.94	0.88
SNORA45	small nucleolar RNA, H/ACA box 45 [Source:HGNC Symbol;Acc:32638]	0.84	0.82	0.76
HTR1A	5-hydroxytryptamine (serotonin) receptor 1A, G protein-coupled [Source:HGNC Symbol;Acc:5286]	0.73	0.71	0.65
FSHR	follicle stimulating hormone receptor [Source:HGNC Symbol;Acc:3969]	0.73	0.72	0.66
OR6C76	olfactory receptor, family 6, subfamily C, member 76 [Source:HGNC Symbol;Acc:31305]	0.94	0.94	0.88
TNS1	tensin 1 [Source:HGNC Symbol;Acc:11973]	0.74	0.62	0.57

CHRNA10	cholinergic receptor, nicotinic, alpha 10 (neuronal) [Source:HGNC Symbol;Acc:13800]	0.83	0.64	0.59
OR2T2	olfactory receptor, family 2, subfamily T, member 2 [Source:HGNC Symbol;Acc:14725]	0.67	0.54	0.49
BMP10	bone morphogenetic protein 10 [Source:HGNC Symbol;Acc:20869]	0.93	0.9	0.85
NHLH2	nescient helix loop helix 2 [Source:HGNC Symbol;Acc:7818]	0.71	0.68	0.63
MUC15	mucin 15, cell surface associated [Source:HGNC Symbol;Acc:14956]	0.95	0.92	0.87
AMZ2	archaelysin family metallopeptidase 2 [Source:HGNC Symbol;Acc:28041]	0.93	0.92	0.87
OVOL1	ovo-like 1(Drosophila) [Source:HGNC Symbol;Acc:8525]	0.75	0.75	0.7
SCARNA10	small Cajal body-specific RNA 10 [Source:HGNC Symbol;Acc:32567]	0.87	0.9	0.82
SGCG	sarcoglycan, gamma (35kDa dystrophin-associated glycoprotein) [Source:HGNC Symbol;Acc:10809]	0.87	0.9	0.82
AIF1	allograft inflammatory factor 1 [Source:HGNC Symbol;Acc:352]	0.68	0.68	0.63
SYBU	syntabulin (syntaxin-interacting) [Source:HGNC Symbol;Acc:26011]	0.89	0.89	0.84
FLNC	filamin C, gamma [Source:HGNC Symbol;Acc:3756]	0.23	0.32	0.18
IGSF22	immunoglobulin superfamily, member 22 [Source:HGNC Symbol;Acc:26750]	0.22	0.11	0.06
VPS25	vacuolar protein sorting 25 homolog (S. cerevisiae) [Source:HGNC Symbol;Acc:28122]	0.11	0.08	0.03
RPH3A	rabphilin 3A homolog (mouse) [Source:HGNC Symbol;Acc:17056]	0.1	0.11	0.05
F2RL2	coagulation factor II (thrombin) receptor-like 2 [Source:HGNC Symbol;Acc:3539]	0.64	0.22	0.17
ATG4A	autophagy related 4A, cysteine peptidase [Source:HGNC Symbol;Acc:16489]	0.37	0.41	0.32
FAM70A	transmembrane protein 255A [Source:HGNC Symbol;Acc:26086]	0.45	0.44	0.39
PSMD10	proteasome (prosome, macropain) 26S subunit, non-ATPase, 10 [Source:HGNC Symbol;Acc:9555]	0.37	0.41	0.32
REG1B	regenerating islet-derived 1 beta [Source:HGNC Symbol;Acc:9952]	0.85	0.83	0.78
KCNS2	potassium voltage-gated channel, delayed-rectifier, subfamily S, member 2 [Source:HGNC Symbol;Acc:6301]	0.83	0.82	0.77
HPX	hemopexin [Source:HGNC Symbol;Acc:5171]	0.86	0.89	0.81
MUSK	muscle, skeletal, receptor tyrosine kinase [Source:HGNC Symbol;Acc:7525]	0.96	0.96	0.91
