# Supplementary material for: Global DNA methylation and transcriptional analyses of human ESC-derived cardiomyocytes
Source: Protein Cell. 2014 Jan 29;5(1):59–68. doi: 10.1007/s13238-013-0016-x (PMC3938846; doi:10.1007/s13238-013-0016-x)
Supplement: Supplementary file 15 — Table S12: Gene association network information for hCM enriched genes that were involved in cardiac transcriptional regulation. This file shows the details of gene-gene interactions among the hCM enriched genes that were involved in cardiac transcriptionalregulation. Network was shown in Figure S2 [file 13238_2013_16_MOESM15_ESM.pdf]

Gene name	Description and ID	CM vs ES	CM vs NSC
SOX2	SRY (sex determining region Y)-box 2 [Source:HGNC Symbol;Acc:11195]	-7.839962992	-7.774789591
PLP1	proteolipid protein 1 [Source:HGNC Symbol;Acc:9086]	-7.224921152	-7.167543891
SOX21	SRY (sex determining region Y)-box 21 [Source:HGNC Symbol;Acc:11197]	-6.963984948	-7.926561808
LIN28A	lin-28 homolog A (C. elegans) [Source:HGNC Symbol;Acc:15986]	-8.607022034	-6.846035447
GLDC	glycine dehydrogenase (decarboxylating) [Source:HGNC Symbol;Acc:4313]	-5.86427679	-5.965852322
ZIC2	Zic family member 2 [Source:HGNC Symbol;Acc:12873]	-8.686744126	-5.740500141
SCG3	secretogranin III [Source:HGNC Symbol;Acc:13707]	-5.834330978	-5.649633255
PTPRZ1	protein tyrosine phosphatase, receptor-type, Z polypeptide 1 [Source:HGNC Symbol;Acc:9685]	-5.727618544	-5.625208291
CAMKV	CaM kinase-like vesicle-associated [Source:HGNC Symbol;Acc:28788]	-5.057126654	-5.660889156
SP8	Sp8 transcription factor [Source:HGNC Symbol;Acc:19196]	-4.92099071	-6.925985278
SFRP2	secreted frizzled-related protein 2 [Source:HGNC Symbol;Acc:10777]	-4.92864031	-4.842781245
CRABP1	cellular retinoic acid binding protein 1 [Source:HGNC Symbol;Acc:2338]	-4.801158849	-8.286504634
KCNQ2	potassium voltage-gated channel, KQT-like subfamily, member 2 [Source:HGNC Symbol;Acc:6296]	-4.769309556	-5.743081305
GNG4	guanine nucleotide binding protein (G protein), gamma 4 [Source:HGNC Symbol;Acc:4407]	-4.657414625	-4.867395733
TOX3	TOX high mobility group box family member 3 [Source:HGNC Symbol;Acc:11972]	-4.645469371	-5.590729917
GPM6B	glycoprotein M6B [Source:HGNC Symbol;Acc:4461]	-4.612015299	-5.802143545
EDNRB	endothelin receptor type B [Source:HGNC Symbol;Acc:3180]	-4.564931386	-4.56939646
FUT9	fucosyltransferase 9 (alpha (1,3) fucosyltransferase) [Source:HGNC Symbol;Acc:4020]	-4.818795907	-4.534002331
GDPD2	glycerophosphodiester phosphodiesterase domain containing 2 [Source:HGNC Symbol;Acc:25974]	-4.54373224	-4.527441968
SLC7A3	solute carrier family 7 (cationic amino acid transporter, y+ system), member 3 [Source:HGNC Symbol;Acc:11061]	-6.871550081	-4.471359566
MEGF10	multiple EGF-like-domains 10 [Source:HGNC Symbol;Acc:29634]	-4.442057546	-4.823518037
USP44	ubiquitin specific peptidase 44 [Source:HGNC Symbol;Acc:20064]	-7.867539796	-4.373767985
CNTNAP2	contactin associated protein-like 2 [Source:HGNC Symbol;Acc:13830]	-5.191150191	-4.343482063
TRIM71	tripartite motif containing 71, E3 ubiquitin protein ligase [Source:HGNC Symbol;Acc:32669]	-5.747294626	-4.296759092
SALL1	sal-like 1 (Drosophila) [Source:HGNC Symbol;Acc:10524]	-4.2858986	-4.621186702
RGMA	RGM domain family, member A [Source:HGNC Symbol;Acc:30308]	-4.169075608	-6.080173361
NELL2	NEL-like 2 (chicken) [Source:HGNC Symbol;Acc:7751]	-4.158390895	-6.022632479
ZIC5	Zic family member 5 [Source:HGNC Symbol;Acc:20322]	-8.194076441	-4.144546832
LOC100509231	ovostatin homolog 2-like	-4.140954396	-4.224717334
LDB2	LIM domain binding 2 [Source:HGNC Symbol;Acc:6533]	-5.81007366	-4.128443604
LPAR4	lysophosphatidic acid receptor 4 [Source:HGNC Symbol;Acc:4478]	-4.092120637	-4.590780171
TEX15	testis expressed 15 [Source:HGNC Symbol;Acc:11738]	-4.086406948	-4.09125063
POU3F1	POU class 3 homeobox 1 [Source:HGNC Symbol;Acc:9214]	-5.65379929	-4.062074478
ZNF423	zinc finger protein 423 [Source:HGNC Symbol;Acc:16762]	-4.028785258	-4.812665928
NPTX1	neuronal pentraxin I [Source:HGNC Symbol;Acc:7952]	-4.113350319	-3.982773235
KIF5C	kinesin family member 5C [Source:HGNC Symbol;Acc:6325]	-3.954629015	-5.141412848
DCLK1	doublecortin-like kinase 1 [Source:HGNC Symbol;Acc:2700]	-3.893754409	-4.614117299
ZIC3	Zic family member 3 [Source:HGNC Symbol;Acc:12874]	-8.019248449	-3.828794475
DPPA4	developmental pluripotency associated 4 [Source:HGNC Symbol;Acc:19200]	-8.039698958	-3.79741387
PPM1E	protein phosphatase, Mg2+/Mn2+ dependent, 1E [Source:HGNC Symbol;Acc:19322]	-3.781747062	-4.941108038
C1orf187	dorsal inhibitory axon guidance protein [Source:HGNC Symbol;Acc:25054]	-3.7250466	-4.913959373
PCDH18	protocadherin 18 [Source:HGNC Symbol;Acc:14268]	-3.712861256	-3.769190761
RAB3C	RAB3C, member RAS oncogene family [Source:HGNC Symbol;Acc:30269]	-3.69419453	-3.945469082
DEPDC1B	DEP domain containing 1B [Source:HGNC Symbol;Acc:24902]	-3.682324665	-3.700435205
ATCAY	ataxia, cerebellar, Cayman type [Source:HGNC Symbol;Acc:779]	-3.679111823	-4.179098401
RAB11FIP4	RAB11 family interacting protein 4 (class II) [Source:HGNC Symbol;Acc:30267]	-3.661309438	-4.475792086
NHLH2	nescient helix loop helix 2 [Source:HGNC Symbol;Acc:7818]	-3.649568484	-6.854177165
MYCL1	v-myc myelocytomatosis viral oncogene homolog 1, lung carcinoma derived (avian) [Source:HGNC Symbol;Acc:7555]	-3.571893501	-3.731715667
FABP7	fatty acid binding protein 7, brain [Source:HGNC Symbol;Acc:3562]	-3.56517929	-5.553519622
SALL4	sal-like 4 (Drosophila) [Source:HGNC Symbol;Acc:15924]	-5.176639541	-3.549479122
LRRN1	leucine rich repeat neuronal 1 [Source:HGNC Symbol;Acc:20980]	-3.690157325	-3.545691701
CTNNA2	catenin (cadherin-associated protein), alpha 2 [Source:HGNC Symbol;Acc:2510]	-3.540637526	-3.591254403
NPTX2	neuronal pentraxin II [Source:HGNC Symbol;Acc:7953]	-3.508729908	-4.80133265
FGFBP3	fibroblast growth factor binding protein 3 [Source:HGNC Symbol;Acc:23428]	-3.488613093	-4.758633121
SOX3	SRY (sex determining region Y)-box 3 [Source:HGNC Symbol;Acc:11199]	-3.487365137	-6.834135838
CCDC88C	coiled-coil domain containing 88C [Source:HGNC Symbol;Acc:19967]	-3.486851308	-3.620045945
CDC7	cell division cycle 7 [Source:HGNC Symbol;Acc:1745]	-3.481602511	-3.6300447
HELLS	helicase, lymphoid-specific [Source:HGNC Symbol;Acc:4861]	-3.563082677	-3.470897269
LIN28B	lin-28 homolog B (C. elegans) [Source:HGNC Symbol;Acc:32207]	-3.895469142	-3.446099009
EFS	embryonal Fyn-associated substrate [Source:HGNC Symbol;Acc:16898]	-3.444370296	-3.578775756
IQGAP2	IQ motif containing GTPase activating protein 2 [Source:HGNC Symbol;Acc:6111]	-3.56455819	-3.434338911
TMEM158	transmembrane protein 158 (gene/pseudogene) [Source:HGNC Symbol;Acc:30293]	-3.656748916	-3.411072344
CBX2	chromobox homolog 2 [Source:HGNC Symbol;Acc:1552]	-3.439598224	-3.409575693
SH3GL2	SH3-domain GRB2-like 2 [Source:HGNC Symbol;Acc:10831]	-3.857888698	-3.397812788
ZNF90	zinc finger protein 90 [Source:HGNC Symbol;Acc:13165]	-5.167377754	-3.395437654
RAPGEF5	Rap guanine nucleotide exchange factor (GEF) 5 [Source:HGNC Symbol;Acc:16862]	-3.364544848	-3.754682084
NHS	Nance-Horan syndrome (congenital cataracts and dental anomalies) [Source:HGNC Symbol;Acc:7820]	-3.358290213	-4.098741873
PTPN5	protein tyrosine phosphatase, non-receptor type 5 (striatum-enriched) [Source:HGNC Symbol;Acc:9657]	-3.540360425	-3.345242812
MIR17HG	microRNA 19a [Source:HGNC Symbol;Acc:31574]	-3.797942211	-3.330138913
PHF21B	PHD finger protein 21B [Source:HGNC Symbol;Acc:25161]	-3.315301134	-4.325174661
MCM10	minichromosome maintenance complex component 10 [Source:HGNC Symbol;Acc:18043]	-3.302537999	-3.571514844
PROM1	prominin 1 [Source:HGNC Symbol;Acc:9454]	-3.29367641	-3.8591924

RNF175	ring finger protein 175 [Source:HGNC Symbol;Acc:27735]	-3.28820287	-3.981979886
WSCD1	WSC domain containing 1 [Source:HGNC Symbol;Acc:29060]	-3.261304161	-3.417103488
FANCD2	Fanconi anemia, complementation group D2 [Source:HGNC Symbol;Acc:3585]	-3.53839393	-3.257923109
FAM64A	family with sequence similarity 64, member A [Source:HGNC Symbol;Acc:25483]	-3.280828763	-3.252523079
C18orf54	chromosome 18 open reading frame 54 [Source:HGNC Symbol;Acc:13796]	-3.816582807	-3.243167893
IMPA2	inositol(myo)-1(or 4)-monophosphatase 2 [Source:HGNC Symbol;Acc:6051]	-4.70418179	-3.239674659
RGS17	regulator of G-protein signaling 17 [Source:HGNC Symbol;Acc:14088]	-3.695549257	-3.219413917
MYB	v-myb myeloblastosis viral oncogene homolog (avian) [Source:HGNC Symbol;Acc:7545]	-3.374179097	-3.214364635
COCH	coagulation factor C homolog, cochlin (Limulus polyphemus) [Source:HGNC Symbol;Acc:2180]	-3.268859979	-3.185550782
C14orf106	MIS18 binding protein 1 [Source:HGNC Symbol;Acc:20190]	-3.329647477	-3.160041393
BLM	Bloom syndrome, RecQ helicase-like [Source:HGNC Symbol;Acc:1058]	-3.189573322	-3.154366064
KIT	v-kit Hardy-Zuckerman 4 feline sarcoma viral oncogene homolog [Source:HGNC Symbol;Acc:6342]	-3.141762543	-3.71993571
RTKN2	rhotekin 2 [Source:HGNC Symbol;Acc:19364]	-3.118215021	-3.453432174
NEFM	neurofilament, medium polypeptide [Source:HGNC Symbol;Acc:7734]	-3.106054392	-7.095495018
C14orf162	coiled-coil domain containing 177 [Source:HGNC Symbol;Acc:23243]	-3.103175299	-3.260421876
TUBB8	tubulin, beta 8 class VIII [Source:HGNC Symbol;Acc:20773]	-3.36803279	-3.096215044
KIAA0101	KIAA0101 [Source:HGNC Symbol;Acc:28961]	-3.094862017	-3.903106969
PSIP1	PC4 and SFRS1 interacting protein 1 [Source:HGNC Symbol;Acc:9527]	-3.755459411	-3.049104995
ATAD5	ATPase family, AAA domain containing 5 [Source:HGNC Symbol;Acc:25752]	-3.440621501	-3.038619072
KIF21B	kinesin family member 21B [Source:HGNC Symbol;Acc:29442]	-3.029568473	-3.75648416
CNTLN	centlein, centrosomal protein [Source:HGNC Symbol;Acc:23432]	-3.037285926	-3.003510708
TAC1	tachykinin, precursor 1 [Source:HGNC Symbol;Acc:11517]	-2.99870726	-3.483406975
RMI1	RMI1, RecQ mediated genome instability 1, homolog (S. cerevisiae) [Source:HGNC Symbol;Acc:25764]	-3.146191083	-2.985720613
CCDC138	coiled-coil domain containing 138 [Source:HGNC Symbol;Acc:26531]	-3.140219262	-2.984977587
ZNF681	zinc finger protein 681 [Source:HGNC Symbol;Acc:26457]	-2.980553547	-3.190044838
CDT1	chromatin licensing and DNA replication factor 1 [Source:HGNC Symbol;Acc:24576]	-3.086333671	-2.968633685
HNRNPU	heterogeneous nuclear ribonucleoprotein U (scaffold attachment factor A) [Source:HGNC Symbol;Acc:5048]	-2.962038564	-2.9890991
BIRC5	baculoviral IAP repeat containing 5 [Source:HGNC Symbol;Acc:593]	-2.944008384	-3.010642809
PELI2	pellino E3 ubiquitin protein ligase family member 2 [Source:HGNC Symbol;Acc:8828]	-3.400712341	-2.928147046
LRRCC1	leucine rich repeat and coiled-coil centrosomal protein 1 [Source:HGNC Symbol;Acc:29373]	-2.923257386	-3.146858579
OVOS2	ovostatin 2	-2.917358306	-3.450396636
GABRB3	gamma-aminobutyric acid (GABA) A receptor, beta 3 [Source:HGNC Symbol;Acc:4083]	-3.787634516	-2.915319951
FAM19A5	family with sequence similarity 19 (chemokine (C-C motif)-like), member A5 [Source:HGNC Symbol;Acc:21592]	-3.030468448	-2.912445615
DBF4	DBF4 homolog (S. cerevisiae) [Source:HGNC Symbol;Acc:17364]	-3.040865565	-2.912227728
GPR19	G protein-coupled receptor 19 [Source:HGNC Symbol;Acc:4473]	-2.910281615	-3.436087935
DSCC1	defective in sister chromatid cohesion 1 homolog (S. cerevisiae) [Source:HGNC Symbol;Acc:24453]	-4.123581102	-2.908907716
GNAL	guanine nucleotide binding protein (G protein), alpha activating activity polypeptide, olfactory type [Source:HGNC Symbol;Acc:4388]	-3.771013868	-2.897689294
CDCA5	cell division cycle associated 5 [Source:HGNC Symbol;Acc:14626]	-2.892954815	-2.937012086
PLCH1	phospholipase C, eta 1 [Source:HGNC Symbol;Acc:29185]	-2.869170703	-4.699135028
TLE1	transducin-like enhancer of split 1 (E(sp1) homolog, Drosophila) [Source:HGNC Symbol;Acc:11837]	-2.863281887	-3.309752688
SHANK2	SH3 and multiple ankyrin repeat domains 2 [Source:HGNC Symbol;Acc:14295]	-3.954659288	-2.855542638
C21orf45	MIS18 kinetochore protein homolog A (S. pombe) [Source:HGNC Symbol;Acc:1286]	-3.429994012	-2.853375537
C1orf106	chromosome 1 open reading frame 106 [Source:HGNC Symbol;Acc:25599]	-2.841242907	-3.539296741
FAM19A4	family with sequence similarity 19 (chemokine (C-C motif)-like), member A4 [Source:HGNC Symbol;Acc:21591]	-2.99529826	-2.829627197
CDC20	cell division cycle 20 [Source:HGNC Symbol;Acc:1723]	-3.140359658	-2.818835975
SALL2	sal-like 2 (Drosophila) [Source:HGNC Symbol;Acc:10526]	-3.293897154	-2.817552006
TMEM194A	transmembrane protein 194A [Source:HGNC Symbol;Acc:29001]	-2.925709694	-2.798612509
WDHD1	WD repeat and HMG-box DNA binding protein 1 [Source:HGNC Symbol;Acc:23170]	-4.004846876	-2.785190229
CENPF	centromere protein F, 350/400kDa [Source:HGNC Symbol;Acc:1857]	-2.779878838	-3.553200001
OLFM1	olfactomedin 1 [Source:HGNC Symbol;Acc:17187]	-3.020349608	-2.775508267
SOHLH2	spermatogenesis and oogenesis specific basic helix-loop-helix 2 [Source:HGNC Symbol;Acc:26026]	-3.134453589	-2.767000679
TMSB15A	thymosin beta 15a [Source:HGNC Symbol;Acc:30744]	-2.763697467	-3.580768461
C16orf88	chromosome 16 open reading frame 88 [Source:HGNC Symbol;Acc:34404]	-3.444400303	-2.761401297
TMPO	thymopoietin [Source:HGNC Symbol;Acc:11875]	-2.754034116	-3.164952042
NKAIN1	Na+/K+ transporting ATPase interacting 1 [Source:HGNC Symbol;Acc:25743]	-2.749606337	-3.473393397
PLK4	polo-like kinase 4 [Source:HGNC Symbol;Acc:11397]	-2.748787333	-2.870475562
STMN3	stathmin-like 3 [Source:HGNC Symbol;Acc:15926]	-2.736153997	-5.397062365
DTL	denticleless E3 ubiquitin protein ligase homolog (Drosophila) [Source:HGNC Symbol;Acc:30288]	-2.923257499	-2.731174854
CENPV	centromere protein V [Source:HGNC Symbol;Acc:29920]	-2.729800002	-3.725464253
PASK	PAS domain containing serine/threonine kinase [Source:HGNC Symbol;Acc:17270]	-3.05183214	-2.684714617
CHRFAM7A	cholinergic receptor, nicotinic, alpha 7 (neuronal) [Source:HGNC Symbol;Acc:1960]	-2.683556202	-2.940300833
ZNF138	zinc finger protein 138 [Source:HGNC Symbol;Acc:12922]	-3.117395647	-2.678789939
ADD2	adducin 2 (beta) [Source:HGNC Symbol;Acc:244]	-3.430613525	-2.676988408
PIF1	PIF1 5'-to-3' DNA helicase homolog (S. cerevisiae) [Source:HGNC Symbol;Acc:26220]	-3.65630298	-2.675838543
SGOL2	shugoshin-like 2 (S. pombe) [Source:HGNC Symbol;Acc:30812]	-2.674371053	-2.669691368
FLVCR1	feline leukemia virus subgroup C cellular receptor 1 [Source:HGNC Symbol;Acc:24682]	-2.852928563	-2.668792582
CHRNA5	cholinergic receptor, nicotinic, alpha 5 (neuronal) [Source:HGNC Symbol;Acc:1959]	-2.676072779	-2.665351419
SALL3	sal-like 3 (Drosophila) [Source:HGNC Symbol;Acc:10527]	-2.655937651	-2.860325698
PRPS2	phosphoribosyl pyrophosphate synthetase 2 [Source:HGNC Symbol;Acc:9465]	-2.645514157	-2.676626286
NARG2	NMDA receptor regulated 2 [Source:HGNC Symbol;Acc:29885]	-2.814949577	-2.634367014
LRIG1	leucine-rich repeats and immunoglobulin-like domains 1 [Source:HGNC Symbol;Acc:17360]	-2.633646705	-3.495252683
TOP2A	topoisomerase (DNA) II alpha 170kDa [Source:HGNC Symbol;Acc:11989]	-2.630821238	-2.713826377

MKI67	antigen identified by monoclonal antibody Ki-67 [Source:HGNC Symbol;Acc:7107]	-2.630121999	-2.708933154
GNG2	guanine nucleotide binding protein (G protein), gamma 2 [Source:HGNC Symbol;Acc:4404]	-2.629944937	-5.751320974
PDE7A	phosphodiesterase 7A [Source:HGNC Symbol;Acc:8791]	-2.696015799	-2.625771226
MYO10	myosin X [Source:HGNC Symbol;Acc:7593]	-3.25793319	-2.625229592
C12orf48	PARP1 binding protein [Source:HGNC Symbol;Acc:26074]	-3.031278803	-2.619537466
CIT	citron (rho-interacting, serine/threonine kinase 21) [Source:HGNC Symbol;Acc:1985]	-2.618203865	-2.663328034
HIST1H2BH	histone cluster 1, H2bh [Source:HGNC Symbol;Acc:4755]	-3.195240211	-2.617254242
GINS1	GINS complex subunit 1 (Psf1 homolog) [Source:HGNC Symbol;Acc:28980]	-2.861568007	-2.616319402
POLE2	polymerase (DNA directed), epsilon 2, accessory subunit [Source:HGNC Symbol;Acc:9178]	-2.952606165	-2.611297679
NLGN4X	neuroligin 4, X-linked [Source:HGNC Symbol;Acc:14287]	-3.740269827	-2.607226467
OIP5	Opa interacting protein 5 [Source:HGNC Symbol;Acc:20300]	-2.952053911	-2.60621875
PAPLN	papilin, proteoglycan-like sulfated glycoprotein [Source:HGNC Symbol;Acc:19262]	-3.190603873	-2.604410617
CXCL3	chemokine (C-X-C motif) ligand 3 [Source:HGNC Symbol;Acc:4604]	-3.108663977	-2.597648375
MND1	meiotic nuclear divisions 1 homolog (S. cerevisiae) [Source:HGNC Symbol;Acc:24839]	-2.714563922	-2.59747157
CHAF1A	chromatin assembly factor 1, subunit A (p150) [Source:HGNC Symbol;Acc:1910]	-2.705828942	-2.591508616
PCDH1	protocadherin 1 [Source:HGNC Symbol;Acc:8655]	-4.628006512	-2.58452412
SMC3	structural maintenance of chromosomes 3 [Source:HGNC Symbol;Acc:2468]	-2.572355994	-2.746105402
SKA3	spindle and kinetochore associated complex subunit 3 [Source:HGNC Symbol;Acc:20262]	-2.712176896	-2.572109481
C6orf168	failed axon connections homolog (Drosophila) [Source:HGNC Symbol;Acc:20742]	-2.564983352	-2.979833089
IRX2	iroquois homeobox 2 [Source:HGNC Symbol;Acc:14359]	-3.046765998	-2.564883534
FZD7	frizzled family receptor 7 [Source:HGNC Symbol;Acc:4045]	-3.662007165	-2.561801814
KIF2C	kinesin family member 2C [Source:HGNC Symbol;Acc:6393]	-2.699352129	-2.557254064
PDE2A	phosphodiesterase 2A, cGMP-stimulated [Source:HGNC Symbol;Acc:8777]	-2.821553386	-2.545747656
NEK2	NIMA-related kinase 2 [Source:HGNC Symbol;Acc:7745]	-2.54518259	-2.662139123
ORC1	origin recognition complex, subunit 1 [Source:HGNC Symbol;Acc:8487]	-2.604581127	-2.542416781
SHISA3	shisa homolog 3 (Xenopus laevis) [Source:HGNC Symbol;Acc:25159]	-2.538087628	-5.45125201
VASH2	vasohibin 2 [Source:HGNC Symbol;Acc:25723]	-5.470773876	-2.526994
KIF15	kinesin family member 15 [Source:HGNC Symbol;Acc:17273]	-2.52658874	-3.318068676
RNASEH2A	ribonuclease H2, subunit A [Source:HGNC Symbol;Acc:18518]	-2.799487757	-2.519783376
FBXO5	F-box protein 5 [Source:HGNC Symbol;Acc:13584]	-2.513304574	-2.564464208
NUDT10	nudix (nucleoside diphosphate linked moiety X)-type motif 10 [Source:HGNC Symbol;Acc:17621]	-2.512125312	-2.514898221
C13orf34	bora, aurora kinase A activator [Source:HGNC Symbol;Acc:24724]	-2.502799919	-2.654995079
NETO1	neuropilin (NRP) and tolloid (TLL)-like 1 [Source:HGNC Symbol;Acc:13823]	-2.502119789	-3.197200288
RAD54L	RAD54-like (S. cerevisiae) [Source:HGNC Symbol;Acc:9826]	-2.978470304	-2.500651243
CHGA	chromogranin A (parathyroid secretory protein 1) [Source:HGNC Symbol;Acc:1929]	-4.115058061	-2.499041736
CA2	carbonic anhydrase II [Source:HGNC Symbol;Acc:1373]	-2.498939933	-3.791676869
CCNB2	cyclin B2 [Source:HGNC Symbol;Acc:1580]	-2.495607011	-2.493604175
HAUS6	HAUS augmin-like complex, subunit 6 [Source:HGNC Symbol;Acc:25948]	-3.071589565	-2.492873454
PPP4R4	protein phosphatase 4, regulatory subunit 4 [Source:HGNC Symbol;Acc:23788]	-3.889974906	-2.489210996
AURKB	aurora kinase B [Source:HGNC Symbol;Acc:11390]	-2.589284169	-2.488219405
ZNF695	zinc finger protein 695 [Source:HGNC Symbol;Acc:30954]	-3.199658661	-2.483965125
HES6	hairy and enhancer of split 6 (Drosophila) [Source:HGNC Symbol;Acc:18254]	-2.482101281	-4.353965196
PRIM1	primase, DNA, polypeptide 1 (49kDa) [Source:HGNC Symbol;Acc:9369]	-3.251238211	-2.478370603
FIGNL2	fidgetin-like 2 [Source:HGNC Symbol;Acc:13287]	-2.476124075	-3.10462778
ST8SIA3	ST8 alpha-N-acetyl-neuraminide alpha-2,8-sialyltransferase 3 [Source:HGNC Symbol;Acc:14269]	-2.471957938	-3.100325429
BTBD17	BTB (POZ) domain containing 17 [Source:HGNC Symbol;Acc:33758]	-2.469919439	-3.810735884
ACBD7	acyl-CoA binding domain containing 7 [Source:HGNC Symbol;Acc:17715]	-2.903025587	-2.466983915
SLC27A2	solute carrier family 27 (fatty acid transporter), member 2 [Source:HGNC Symbol;Acc:10996]	-4.544801875	-2.462667615
KNTC1	kinetochore associated 1 [Source:HGNC Symbol;Acc:17255]	-2.697090219	-2.460524371
ARHGAP11A	Rho GTPase activating protein 11A [Source:HGNC Symbol;Acc:15783]	-2.728889493	-2.460356225
MNS1	meiosis-specific nuclear structural 1 [Source:HGNC Symbol;Acc:29636]	-2.459168962	-2.90790214
BRCA1	breast cancer 1, early onset [Source:HGNC Symbol;Acc:1100]	-2.457377335	-3.075293646
MAP2K6	mitogen-activated protein kinase kinase 6 [Source:HGNC Symbol;Acc:6846]	-2.457330383	-2.89736236
ARMC4	armadillo repeat containing 4 [Source:HGNC Symbol;Acc:25583]	-3.077878219	-2.446916145
FEZ1	fasciculation and elongation protein zeta 1 (zygin I) [Source:HGNC Symbol;Acc:3659]	-2.559768099	-2.443710794
ZNF551	zinc finger protein 551 [Source:HGNC Symbol;Acc:25108]	-3.337901684	-2.442893384
ARRB2	arrestin, beta 2 [Source:HGNC Symbol;Acc:712]	-2.614106742	-2.434356338
STEAP1	six transmembrane epithelial antigen of the prostate 1 [Source:HGNC Symbol;Acc:11378]	-3.455444632	-2.4340855
ZNF670	zinc finger protein 670 [Source:HGNC Symbol;Acc:28167]	-2.429368638	-2.462832127
HMGB3	high mobility group box 3 [Source:HGNC Symbol;Acc:5004]	-2.578240122	-2.426992565
MCM2	minichromosome maintenance complex component 2 [Source:HGNC Symbol;Acc:6944]	-2.422636029	-2.825839029
CCNF	cyclin F [Source:HGNC Symbol;Acc:1591]	-2.671935327	-2.421658132
MCM7	minichromosome maintenance complex component 7 [Source:HGNC Symbol;Acc:6950]	-2.416301799	-2.472126399
ARL6IP6	ADP-ribosylation-like factor 6 interacting protein 6 [Source:HGNC Symbol;Acc:24048]	-2.497803303	-2.408416361
CCNA2	cyclin A2 [Source:HGNC Symbol;Acc:1578]	-2.406753974	-2.408894012
KIF14	kinesin family member 14 [Source:HGNC Symbol;Acc:19181]	-2.585169285	-2.404478107
RCC1	regulator of chromosome condensation 1 [Source:HGNC Symbol;Acc:1913]	-2.400507953	-2.405259788
ESPL1	extra spindle pole bodies homolog 1 (S. cerevisiae) [Source:HGNC Symbol;Acc:16856]	-2.399080024	-2.942828633
C1orf112	chromosome 1 open reading frame 112 [Source:HGNC Symbol;Acc:25565]	-2.545270113	-2.398088182
ZNF519	zinc finger protein 519 [Source:HGNC Symbol;Acc:30574]	-2.66195624	-2.396866981
HNRNPD	heterogeneous nuclear ribonucleoprotein D (AU-rich element RNA binding protein 1, 37kDa) [Source:HGNC Symbol;Acc:5036]	-2.395209695	-2.45022425
C9orf140	N/A	-2.478220422	-2.389809355
ALPL	alkaline phosphatase, liver/bone/kidney [Source:HGNC Symbol;Acc:438]	-3.060848552	-2.387369401
DLEU7	deleted in lymphocytic leukemia, 7 [Source:HGNC Symbol;Acc:17567]	-2.387297636	-4.587383805
MAD2L1	MAD2 mitotic arrest deficient-like 1 (yeast) [Source:HGNC Symbol;Acc:6763]	-2.43187875	-2.386721038

DNMT3B	DNA (cytosine-5-)-methyltransferase 3 beta [Source:HGNC Symbol;Acc:2979]	-6.012814171	-2.381845066
FANCI	Fanconi anemia, complementation group I [Source:HGNC Symbol;Acc:25568]	-2.377810728	-2.653888863
MBD2	methyl-CpG binding domain protein 2 [Source:HGNC Symbol;Acc:6917]	-3.49837286	-2.375102222
TTK	TTK protein kinase [Source:HGNC Symbol;Acc:12401]	-2.631666045	-2.374155059
ZNF92	zinc finger protein 92 [Source:HGNC Symbol;Acc:13168]	-2.365821539	-2.462084665
SOX8	SRY (sex determining region Y)-box 8 [Source:HGNC Symbol;Acc:11203]	-2.365616734	-4.703080052
ZDHHC22	zinc finger, DHHC-type containing 22 [Source:HGNC Symbol;Acc:20106]	-2.720793011	-2.359845759
MLF1IP	MLF1 interacting protein [Source:HGNC Symbol;Acc:21348]	-2.450520175	-2.357249617
FAM54A	mitochondrial fission regulator 2 [Source:HGNC Symbol;Acc:21115]	-2.665772723	-2.355242754
ERCC6L	excision repair cross-complementing rodent repair deficiency, complementation group 6-like [Source:HGNC Symbol;Acc:20794]	-3.221690196	-2.347545818
CDC25C	cell division cycle 25C [Source:HGNC Symbol;Acc:1727]	-2.616314942	-2.347167344
TUBB2B	tubulin, beta 2B class IIb [Source:HGNC Symbol;Acc:30829]	-2.344667701	-3.093042512
MCM6	minichromosome maintenance complex component 6 [Source:HGNC Symbol;Acc:6949]	-2.467672404	-2.338679028
VRK1	vaccinia related kinase 1 [Source:HGNC Symbol;Acc:12718]	-2.900813463	-2.336559141
IDH1	isocitrate dehydrogenase 1 (NADP+), soluble [Source:HGNC Symbol;Acc:5382]	-2.333667921	-2.370309803
TCERG1	transcription elongation regulator 1 [Source:HGNC Symbol;Acc:15630]	-2.671790777	-2.327523557
CALML4	calmodulin-like 4 [Source:HGNC Symbol;Acc:18445]	-2.596187058	-2.325648076
ECT2	epithelial cell transforming sequence 2 oncogene [Source:HGNC Symbol;Acc:3155]	-2.594187269	-2.323204752
C4orf21	chromosome 4 open reading frame 21 [Source:HGNC Symbol;Acc:25654]	-2.910903121	-2.32308981
NCAPH	non-SMC condensin I complex, subunit H [Source:HGNC Symbol;Acc:1112]	-2.32271253	-2.424323634
ZNF114	zinc finger protein 114 [Source:HGNC Symbol;Acc:12894]	-3.251773187	-2.320152123
SEMA5B	sema domain, seven thrombospondin repeats (type 1 and type 1-like), transmembrane domain (TM) and short cytoplasmic domain, (semaphorin) 5B [Source:HGNC Symbol;Acc:10737]	-2.319834659	-5.150889037
TAF1A	TATA box binding protein (TBP)-associated factor, RNA polymerase I, A, 48kDa [Source:HGNC Symbol;Acc:11532]	-2.463845024	-2.317282057
KIFC1	kinesin family member C1 [Source:HGNC Symbol;Acc:6389]	-2.314358439	-2.428275671
PRIM2	primase, DNA, polypeptide 2 (58kDa) [Source:HGNC Symbol;Acc:9370]	-2.419633795	-2.3115573
CA14	carbonic anhydrase XIV [Source:HGNC Symbol;Acc:1372]	-3.987454285	-2.311536156
NMU	neuromedin U [Source:HGNC Symbol;Acc:7859]	-2.30910431	-3.322841062
TOP1MT	topoisomerase (DNA) I, mitochondrial [Source:HGNC Symbol;Acc:29787]	-2.945097323	-2.302339746
GPRC5B	G protein-coupled receptor, family C, group 5, member B [Source:HGNC Symbol;Acc:13308]	-3.010460138	-2.297505121
ADRBK2	adrenergic, beta, receptor kinase 2 [Source:HGNC Symbol;Acc:290]	-2.410153922	-2.297424381
B3GAT1	beta-1,3-glucuronyltransferase 1 (glucuronosyltransferase P) [Source:HGNC Symbol;Acc:921]	-2.29662799	-2.570590355
RHBDL3	rhomboid, veinlet-like 3 (Drosophila) [Source:HGNC Symbol;Acc:16502]	-2.294661688	-4.218120694
NUSAP1	nucleolar and spindle associated protein 1 [Source:HGNC Symbol;Acc:18538]	-2.394257803	-2.292969991
RGS16	regulator of G-protein signaling 16 [Source:HGNC Symbol;Acc:9997]	-2.2917063	-4.124242911
ZNF232	zinc finger protein 232 [Source:HGNC Symbol;Acc:13026]	-2.39322047	-2.290547842
TAF5	TAF5 RNA polymerase II, TATA box binding protein (TBP)-associated factor, 100kDa [Source:HGNC Symbol;Acc:11539]	-2.432040126	-2.281609569
MOBKL2B	MOB kinase activator 3B [Source:HGNC Symbol;Acc:23825]	-2.281028674	-3.594146986
RQCD1	RCD1 required for cell differentiation1 homolog (S. pombe) [Source:HGNC Symbol;Acc:10445]	-2.280646322	-2.416491151
NCAPG	non-SMC condensin I complex, subunit G [Source:HGNC Symbol;Acc:24304]	-2.5187037	-2.276068471
EXO1	exonuclease 1 [Source:HGNC Symbol;Acc:3511]	-2.702491832	-2.275191812
RRM1	ribonucleotide reductase M1 [Source:HGNC Symbol;Acc:10451]	-2.274163054	-2.623006431
C9orf100	Rho guanine nucleotide exchange factor (GEF) 39 [Source:HGNC Symbol;Acc:25909]	-3.488044348	-2.273683909
GLI1	GLI family zinc finger 1 [Source:HGNC Symbol;Acc:4317]	-2.273476724	-3.852324237
VAV3	vav 3 guanine nucleotide exchange factor [Source:HGNC Symbol;Acc:12659]	-2.635938264	-2.272105367
ZNF682	zinc finger protein 682 [Source:HGNC Symbol;Acc:28857]	-2.270756883	-2.271614105
CALB1	calbindin 1, 28kDa [Source:HGNC Symbol;Acc:1434]	-3.742749525	-2.270053362
RAD51AP1	RAD51 associated protein 1 [Source:HGNC Symbol;Acc:16956]	-2.53465196	-2.269603474
FGFR3	fibroblast growth factor receptor 3 [Source:HGNC Symbol;Acc:3690]	-2.267828707	-3.825215155
TIAM1	T-cell lymphoma invasion and metastasis 1 [Source:HGNC Symbol;Acc:11805]	-2.265658934	-2.579514353
SSRP1	structure specific recognition protein 1 [Source:HGNC Symbol;Acc:11327]	-2.264186187	-2.500146101
CENPJ	centromere protein J [Source:HGNC Symbol;Acc:17272]	-2.264052654	-2.426834944
CCDC109B	coiled-coil domain containing 109B [Source:HGNC Symbol;Acc:26076]	-2.258092844	-2.757959764
GINS4	GINS complex subunit 4 (Sld5 homolog) [Source:HGNC Symbol;Acc:28226]	-2.257828866	-2.325402642
BRCA2	breast cancer 2, early onset [Source:HGNC Symbol;Acc:1101]	-2.252654538	-2.319188966
RAB8B	RAB8B, member RAS oncogene family [Source:HGNC Symbol;Acc:30273]	-2.250593072	-3.089149133
C11orf70	chromosome 11 open reading frame 70 [Source:HGNC Symbol;Acc:28188]	-2.250140909	-3.247195947
DLGAP5	discs, large (Drosophila) homolog-associated protein 5 [Source:HGNC Symbol;Acc:16864]	-2.808583613	-2.249328009
C16orf59	chromosome 16 open reading frame 59 [Source:HGNC Symbol;Acc:25849]	-2.247417517	-2.323433818
HIST1H4J	histone cluster 1, H4j [Source:HGNC Symbol;Acc:4785]	-2.348355186	-2.245712241
IGDCC3	immunoglobulin superfamily, DCC subclass, member 3 [Source:HGNC Symbol;Acc:9700]	-2.244111169	-5.44112511
TRERF1	transcriptional regulating factor 1 [Source:HGNC Symbol;Acc:18273]	-2.242090265	-2.520670791
IGFBPL1	insulin-like growth factor binding protein-like 1 [Source:HGNC Symbol;Acc:20081]	-2.241463219	-3.559220967
E2F7	E2F transcription factor 7 [Source:HGNC Symbol;Acc:23820]	-2.240348237	-2.753184872
ANKRD32	ankyrin repeat domain 32 [Source:HGNC Symbol;Acc:25408]	-2.336187563	-2.239382838
TIMELESS	timeless circadian clock [Source:HGNC Symbol;Acc:11813]	-2.239351258	-2.527283518
NUP50	nucleoporin 50kDa [Source:HGNC Symbol;Acc:8065]	-2.241732437	-2.237552617
THBS4	thrombospondin 4 [Source:HGNC Symbol;Acc:11788]	-3.302022143	-2.235367308
SPAG5	sperm associated antigen 5 [Source:HGNC Symbol;Acc:13452]	-2.45307556	-2.234909649
PSMC3IP	PSMC3 interacting protein [Source:HGNC Symbol;Acc:17928]	-2.230801668	-2.545006103
ILF3	interleukin enhancer binding factor 3, 90kDa [Source:HGNC Symbol;Acc:6038]	-2.229646311	-2.388231533
RNF144A	ring finger protein 144A [Source:HGNC Symbol;Acc:20457]	-2.227575671	-3.473033944
EPB41L2	erythrocyte membrane protein band 4.1-like 2 [Source:HGNC Symbol;Acc:3379]	-2.6445657	-2.223204374

NCRNA00201	HNRNPU antisense RNA 1 [Source:HGNC Symbol;Acc:27651]	-2.828809691	-2.222269651
ASPM	asp (abnormal spindle) homolog, microcephaly associated (Drosophila) [Source:HGNC Symbol;Acc:19048]	-2.222066926	-2.233170218
SMC2	structural maintenance of chromosomes 2 [Source:HGNC Symbol;Acc:14011]	-2.221850829	-2.490659444
BCL2L11	BCL2-like 11 (apoptosis facilitator) [Source:HGNC Symbol;Acc:994]	-2.83952762	-2.220318315
FAM49A	family with sequence similarity 49, member A [Source:HGNC Symbol;Acc:25373]	-2.220255555	-4.032175602
NUF2	NUF2, NDC80 kinetochore complex component, homolog (S. cerevisiae) [Source:HGNC Symbol;Acc:14621]	-2.219527572	-2.413752347
KIF11	kinesin family member 11 [Source:HGNC Symbol;Acc:6388]	-2.216932811	-2.642091739
PSAT1	phosphoserine aminotransferase 1 [Source:HGNC Symbol;Acc:19129]	-2.78828642	-2.214870693
RFC5	replication factor C (activator 1) 5, 36.5kDa [Source:HGNC Symbol;Acc:9973]	-2.214786116	-2.422431456
C6orf174	SOGA family member 3 [Source:HGNC Symbol;Acc:21494]	-2.214281499	-4.39442214
HAUS5	HAUS augmin-like complex, subunit 5 [Source:HGNC Symbol;Acc:29130]	-2.214088142	-2.83283596
MAP7	microtubule-associated protein 7 [Source:HGNC Symbol;Acc:6869]	-4.169013574	-2.210358933
NCAPD2	non-SMC condensin I complex, subunit D2 [Source:HGNC Symbol;Acc:24305]	-2.29243702	-2.204773339
RNF182	ring finger protein 182 [Source:HGNC Symbol;Acc:28522]	-2.203423456	-2.697909545
RFC3	replication factor C (activator 1) 3, 38kDa [Source:HGNC Symbol;Acc:9971]	-2.464885304	-2.200561979
SYT1	synaptotagmin I [Source:HGNC Symbol;Acc:11509]	-2.190091588	-2.845890938
RPL31	ribosomal protein L31 [Source:HGNC Symbol;Acc:10334]	-2.18385781	-2.250515188
RPRML	reprimo-like [Source:HGNC Symbol;Acc:32422]	-2.182478038	-3.949806825
TTYH1	tweety homolog 1 (Drosophila) [Source:HGNC Symbol;Acc:13476]	-2.181136547	-4.963133288
STIL	SCL/TAL1 interrupting locus [Source:HGNC Symbol;Acc:10879]	-2.576796319	-2.17462472
MELK	maternal embryonic leucine zipper kinase [Source:HGNC Symbol;Acc:16870]	-2.174256831	-2.192468834
TET1	tet methylcytosine dioxygenase 1 [Source:HGNC Symbol;Acc:29484]	-3.762141846	-2.173289446
BRIP1	BRCA1 interacting protein C-terminal helicase 1 [Source:HGNC Symbol;Acc:20473]	-2.17006504	-2.252063078
RIF1	RAP1 interacting factor homolog (yeast) [Source:HGNC Symbol;Acc:23207]	-2.874039382	-2.16906343
IFITM1	interferon induced transmembrane protein 1 [Source:HGNC Symbol;Acc:5412]	-5.001258855	-2.166445875
PKN3	protein kinase N3 [Source:HGNC Symbol;Acc:17999]	-2.176247152	-2.159476621
NASP	nuclear autoantigenic sperm protein (histone-binding) [Source:HGNC Symbol;Acc:7644]	-2.306475422	-2.158105682
TDP1	tyrosyl-DNA phosphodiesterase 1 [Source:HGNC Symbol;Acc:18884]	-2.626868416	-2.157314924
RBMX	RNA binding motif protein, X-linked [Source:HGNC Symbol;Acc:9910]	-2.156171602	-2.291295609
CCDC136	coiled-coil domain containing 136 [Source:HGNC Symbol;Acc:22225]	-2.136027865	-2.233196764
41155	N/A	-2.135658418	-3.728140727
TMEM132B	transmembrane protein 132B [Source:HGNC Symbol;Acc:29397]	-2.493342377	-2.135477369
GINS2	GINS complex subunit 2 (Psf2 homolog) [Source:HGNC Symbol;Acc:24575]	-2.13008454	-2.76944041
MRE11A	MRE11 meiotic recombination 11 homolog A (S. cerevisiae) [Source:HGNC Symbol;Acc:7230]	-2.210055664	-2.129763061
MCM4	minichromosome maintenance complex component 4 [Source:HGNC Symbol;Acc:6947]	-2.438621806	-2.128408579
ZNF100	zinc finger protein 100 [Source:HGNC Symbol;Acc:12880]	-2.128139502	-2.444876203
HMMR	hyaluronan-mediated motility receptor (RHAMM) [Source:HGNC Symbol;Acc:5012]	-3.146181828	-2.126014518
GSG2	germ cell associated 2 (haspin) [Source:HGNC Symbol;Acc:19682]	-2.51332707	-2.125017233
HNRNPA1	heterogeneous nuclear ribonucleoprotein A1 [Source:HGNC Symbol;Acc:5031]	-2.128228985	-2.124985642
SUV39H2	suppressor of variegation 3-9 homolog 2 (Drosophila) [Source:HGNC Symbol;Acc:17287]	-2.115627579	-2.834962036
CDC45	cell division cycle 45 [Source:HGNC Symbol;Acc:1739]	-2.11346827	-2.238623225
SAAL1	serum amyloid A-like 1 [Source:HGNC Symbol;Acc:25158]	-2.174569032	-2.112885766
CCDC150	coiled-coil domain containing 150 [Source:HGNC Symbol;Acc:26834]	-2.110986812	-2.197094608
EPB41	erythrocyte membrane protein band 4.1 (elliptocytosis 1, RH-linked) [Source:HGNC Symbol;Acc:3377]	-2.110132195	-3.122737968
CDCA7L	cell division cycle associated 7-like [Source:HGNC Symbol;Acc:30777]	-3.171393909	-2.109082224
ATPBD4	ATP binding domain 4 [Source:HGNC Symbol;Acc:30543]	-2.996330975	-2.108971376
FAM108B1	family with sequence similarity 108, member B1 [Source:HGNC Symbol;Acc:24278]	-2.712689621	-2.107826826
ITGB3BP	integrin beta 3 binding protein (beta3-endonexin) [Source:HGNC Symbol;Acc:6157]	-2.106588892	-2.135475294
RFC4	replication factor C (activator 1) 4, 37kDa [Source:HGNC Symbol;Acc:9972]	-2.320674997	-2.104170286
MUTYH	mutY homolog (E. coli) [Source:HGNC Symbol;Acc:7527]	-2.597327663	-2.101936967
CAMTA1	calmodulin binding transcription activator 1 [Source:HGNC Symbol;Acc:18806]	-2.097765105	-3.027713194
SGOL1	shugoshin-like 1 (S. pombe) [Source:HGNC Symbol;Acc:25088]	-2.328737052	-2.096917561
LOC100509382	putative tubulin beta-4q chain-like	-2.614749369	-2.09637035
DDIT4	DNA-damage-inducible transcript 4 [Source:HGNC Symbol;Acc:24944]	-3.911478645	-2.091862493
KCNK5	potassium channel, subfamily K, member 5 [Source:HGNC Symbol;Acc:6280]	-4.857163076	-2.091108819
ZIK1	zinc finger protein interacting with K protein 1 [Source:HGNC Symbol;Acc:33104]	-2.090491253	-2.349948101
LEMD1	LEM domain containing 1 [Source:HGNC Symbol;Acc:18725]	-2.514779333	-2.090332987
LONRF1	LON peptidase N-terminal domain and ring finger 1 [Source:HGNC Symbol;Acc:26302]	-2.249790332	-2.088701418
SYT14	synaptotagmin XIV [Source:HGNC Symbol;Acc:23143]	-2.505408018	-2.088226107
FANCA	Fanconi anemia, complementation group A [Source:HGNC Symbol;Acc:3582]	-2.189838774	-2.087890943
GNAZ	guanine nucleotide binding protein (G protein), alpha z polypeptide [Source:HGNC Symbol;Acc:4395]	-2.08747227	-2.823795805
TRIM59	tripartite motif containing 59 [Source:HGNC Symbol;Acc:30834]	-2.356201289	-2.084866139
MAGOHB	mago-nashi homolog, proliferation-associated (Drosophila) [Source:HGNC Symbol;Acc:6815]	-2.078991196	-2.945580305
ZNF331	zinc finger protein 331 [Source:HGNC Symbol;Acc:15489]	-3.100962704	-2.078110155
PAK3	p21 protein (Cdc42/Rac)-activated kinase 3 [Source:HGNC Symbol;Acc:8592]	-2.076337565	-3.633193459
FAM161A	family with sequence similarity 161, member A [Source:HGNC Symbol;Acc:25808]	-2.074982962	-2.799760046
FGD4	FYVE, RhoGEF and PH domain containing 4 [Source:HGNC Symbol;Acc:19125]	-2.793963741	-2.073599517
SEPHS1	selenophosphate synthetase 1 [Source:HGNC Symbol;Acc:19685]	-2.866451237	-2.070318234
FKBP5	FK506 binding protein 5 [Source:HGNC Symbol;Acc:3721]	-2.58116352	-2.070251045
CHRDL1	chordin-like 1 [Source:HGNC Symbol;Acc:29861]	-2.06820709	-2.203120963
DPYSL2	dihydropyrimidinase-like 2 [Source:HGNC Symbol;Acc:3014]	-2.066905978	-3.053793106
TACC3	transforming, acidic coiled-coil containing protein 3 [Source:HGNC Symbol;Acc:11524]	-2.066179372	-2.089537568
OLFML2B	olfactomedin-like 2B [Source:HGNC Symbol;Acc:24558]	-2.061999133	-2.53900979
ZNF85	zinc finger protein 85 [Source:HGNC Symbol;Acc:13160]	-2.306112684	-2.061702315

CDO1	cysteine dioxygenase, type I [Source:HGNC Symbol;Acc:1795]	-2.058034315	-2.673572635
NOTCH1	notch 1 [Source:HGNC Symbol;Acc:7881]	-2.056368642	-2.630689214
TRDMT1	tRNA aspartic acid methyltransferase 1 [Source:HGNC Symbol;Acc:2977]	-2.051341839	-2.244903859
FNBP1L	formin binding protein 1-like [Source:HGNC Symbol;Acc:20851]	-2.842914145	-2.051292175
SLC16A9	solute carrier family 16, member 9 (monocarboxylic acid transporter 9) [Source:HGNC Symbol;Acc:23520]	-2.050571445	-3.690382532
NXT2	nuclear transport factor 2-like export factor 2 [Source:HGNC Symbol;Acc:18151]	-2.419622556	-2.048151847
TCF3	transcription factor 3 (E2A immunoglobulin enhancer binding factors E12/E47) [Source:HGNC Symbol;Acc:11633]	-2.048141663	-2.401936791
C7orf68	hypoxia inducible lipid droplet-associated [Source:HGNC Symbol;Acc:28859]	-2.045540552	-2.469271384
PTPRN2	protein tyrosine phosphatase, receptor type, N polypeptide 2 [Source:HGNC Symbol;Acc:9677]	-2.04509321	-6.221786533
GJB7	gap junction protein, beta 7, 25kDa [Source:HGNC Symbol;Acc:16690]	-2.201536494	-2.042642424
SHMT1	serine hydroxymethyltransferase 1 (soluble) [Source:HGNC Symbol;Acc:10850]	-2.379192416	-2.041838417
UBE2C	ubiquitin-conjugating enzyme E2C [Source:HGNC Symbol;Acc:15937]	-2.24597532	-2.040975304
TMX1	thioredoxin-related transmembrane protein 1 [Source:HGNC Symbol;Acc:15487]	-2.058703611	-2.039255355
TMEM48	transmembrane protein 48 [Source:HGNC Symbol;Acc:25525]	-2.192736742	-2.037843804
UHRF1	ubiquitin-like with PHD and ring finger domains 1 [Source:HGNC Symbol;Acc:12556]	-2.035987062	-3.446516416
CDCA2	cell division cycle associated 2 [Source:HGNC Symbol;Acc:14623]	-2.273553844	-2.034295447
PRR11	proline rich 11 [Source:HGNC Symbol;Acc:25619]	-2.034242806	-2.138530144
ESCO2	establishment of cohesion 1 homolog 2 (S. cerevisiae) [Source:HGNC Symbol;Acc:27230]	-2.033528812	-2.26936026
SYNCRIP	synaptotagmin binding, cytoplasmic RNA interacting protein [Source:HGNC Symbol;Acc:16918]	-2.185141019	-2.028384683
AMD1	adenosylmethionine decarboxylase 1 [Source:HGNC Symbol;Acc:457]	-2.364759511	-2.026018326
SGK494	uncharacterized serine/threonine-protein kinase SgK494 [Source:RefSeq peptide;Acc:NP_001167574]	-2.538704382	-2.024955371
HNRNPC	heterogeneous nuclear ribonucleoprotein C (C1/C2) [Source:HGNC Symbol;Acc:5035]	-2.021932058	-2.067820846
TMEM132D	transmembrane protein 132D [Source:HGNC Symbol;Acc:29411]	-2.017738345	-2.717078719
RARRES2	retinoic acid receptor responder (tazarotene induced) 2 [Source:HGNC Symbol;Acc:9868]	-2.659586218	-2.016906431
PHLPP1	PH domain and leucine rich repeat protein phosphatase 1 [Source:HGNC Symbol;Acc:20610]	-2.579503845	-2.016256019
ZNF826P	zinc finger protein 826, pseudogene [Source:HGNC Symbol;Acc:33875]	-2.786720511	-2.01605884
CCNB1	cyclin B1 [Source:HGNC Symbol;Acc:1579]	-2.008895861	-2.017150384
FOXM1	forkhead box M1 [Source:HGNC Symbol;Acc:3818]	-2.007431147	-2.42727041
KIF4A	kinesin family member 4A [Source:HGNC Symbol;Acc:13339]	-2.005417713	-2.764455004
KHDRBS2	KH domain containing, RNA binding, signal transduction associated 2 [Source:HGNC Symbol;Acc:18114]	-2.003281259	-3.446452148
TKT	transketolase [Source:HGNC Symbol;Acc:11834]	-2.760988944	-2.002753037
RAB39B	RAB39B, member RAS oncogene family [Source:HGNC Symbol;Acc:16499]	-2.064679165	-2.001740133
ZNF642	ZFP69 zinc finger protein [Source:HGNC Symbol;Acc:24708]	-2.00079959	-2.158592559
BTBD3	BTB (POZ) domain containing 3 [Source:HGNC Symbol;Acc:15854]	-2.00024959	-2.010816369
ARL6IP1	ADP-ribosylation factor-like 6 interacting protein 1 [Source:HGNC Symbol;Acc:697]	-2.00024308	-2.371989186
ZNF708	zinc finger protein 708 [Source:HGNC Symbol;Acc:12945]	-2.125541277	-2.000223939
