# Supplementary material for: Global DNA methylation and transcriptional analyses of human ESC-derived cardiomyocytes
Source: Protein Cell. 2014 Jan 29;5(1):59–68. doi: 10.1007/s13238-013-0016-x (PMC3938846; doi:10.1007/s13238-013-0016-x)
Supplement: Supplementary file 16 — Table S13: Gene-disease network information for the top hCM up-regulated genes. This file lists all the genes that show association with cardiovascular diseases out of the top 50 up-regulated hCM enriched genes. It also contains the detailed information (nodes and edges) of the gene-disease network that is shown as Figure 1D [file 13238_2013_16_MOESM16_ESM.pdf]

Gene Name	Description and ID	DNA methylation			Gene expression	
		ES-mCpG	NSC-mCpG	CM-mCpG	CM vs ES	CM vs NSC
POPDC2	popeye domain containing 2 [Source:HGNC Symbol;Acc:17648]	0.93	0.94	0.53	8.402616293	7.562352603
ALPK2	alpha-kinase 2 [Source:HGNC Symbol;Acc:20565]	0.78	0.59	0.25	8.069528368	3.647151766
TRIM55	tripartite motif containing 55 [Source:HGNC Symbol;Acc:14215]	0.89	0.67	0.34	7.373137025	7.267597143
ASPH	aspartate beta-hydroxylase [Source:HGNC Symbol;Acc:757]	0.83	0.93	0.53	4.281290866	3.122582294
FILIP1	filamin A interacting protein 1 [Source:HGNC Symbol;Acc:21015]	0.91	0.75	0.54	6.920428571	7.794390913
HSPB7	heat shock 27kDa protein family, member 7 (cardiovascular) [Source:HGNC Symbol;Acc:5249]	0.91	0.74	0.53	6.447867991	6.295762763
NPPA	natriuretic peptide A [Source:HGNC Symbol;Acc:7939]	0.93	0.91	0.73	7.983519001	8.03271611
KBTBD10	kelch-like 41 (Drosophila) [Source:HGNC Symbol;Acc:16905]	0.9	0.91	0.72	2.910404942	2.670527553
MYL4	myosin, light chain 4, alkali; atrial, embryonic [Source:HGNC Symbol;Acc:7585]	0.92	0.81	0.64	9.22112583	9.002175408
CRYAB	crystallin, alpha B [Source:HGNC Symbol;Acc:2389]	0.94	0.85	0.68	5.935066651	6.305402948
LRRN4	leucine rich repeat neuronal 4 [Source:HGNC Symbol;Acc:16208]	0.68	0.71	0.53	2.491450066	2.533850351
MYOZ2	myozenin 2 [Source:HGNC Symbol;Acc:1330]	0.97	0.96	0.83	9.48535322	9.510284971
OBSCN	obscurin, cytoskeletal calmodulin and titin-interacting RhoGEF [Source:HGNC Symbol;Acc:15719]	0.9	0.86	0.73	2.598541342	2.401037422
SMPX	small muscle protein, X-linked [Source:HGNC Symbol;Acc:11122]	0.92	0.85	0.73	9.91336154	9.678290914
NRK	Nik related kinase [Source:HGNC Symbol;Acc:25391]	0.48	0.51	0.38	2.403506377	4.248352806
ABLIM1	actin binding LIM protein 1 [Source:HGNC Symbol;Acc:78]	0.83	0.83	0.73	2.257898689	2.653017499
SYNPO2L	synaptopodin 2-like [Source:HGNC Symbol;Acc:23532]	0.9	0.93	0.82	9.25673506	9.420535272
ANKRD1	ankyrin repeat domain 1 (cardiac muscle) [Source:HGNC Symbol;Acc:15819]	0.25	0.18	0.1	9.714009013	8.638574866
TNNI1	troponin I type 1 (skeletal, slow) [Source:HGNC Symbol;Acc:11945]	0.79	0.7	0.62	6.705106323	6.794815577
MYH6	myosin, heavy chain 6, cardiac muscle, alpha [Source:HGNC Symbol;Acc:7576]	0.87	0.76	0.69	9.518535857	9.794227963
ABRA	actin-binding Rho activating protein [Source:HGNC Symbol;Acc:30655]	0.97	0.92	0.85	5.464788018	5.340692628
TPM1	tropomyosin 1 (alpha) [Source:HGNC Symbol;Acc:12010]	0.77	0.88	0.7	3.637236226	6.143185497
C15orf52	chromosome 15 open reading frame 52 [Source:HGNC Symbol;Acc:33488]	0.87	0.91	0.8	5.192978911	5.918630472
MYOM1	myomesin 1 [Source:HGNC Symbol;Acc:7613]	0.92	0.9	0.84	5.928375111	5.942358498
MASP1	mannan-binding lectin serine peptidase 1 (C4/C2 activating component of Ra-reactive factor) [Source:HGNC Symbol;Acc:6901]	0.54	0.49	0.43	7.240842303	6.569487061
MYBPC3	myosin binding protein C, cardiac [Source:HGNC Symbol;Acc:7551]	0.89	0.83	0.77	6.01967711	6.115402912
TNS1	tensin 1 [Source:HGNC Symbol;Acc:11973]	0.74	0.62	0.57	2.641474521	2.546919208
FLNC	filamin C, gamma [Source:HGNC Symbol;Acc:3756]	0.23	0.32	0.18	5.199968343	4.370411535
F2RL2	coagulation factor II (thrombin) receptor-like 2 [Source:HGNC Symbol;Acc:3539]	0.64	0.22	0.17	3.218976947	3.106845011
