# Supplementary material for: Global DNA methylation and transcriptional analyses of human ESC-derived cardiomyocytes
Source: Protein Cell. 2014 Jan 29;5(1):59–68. doi: 10.1007/s13238-013-0016-x (PMC3938846; doi:10.1007/s13238-013-0016-x)
Supplement: Supplementary file 20 — Table S17: GO analysis of hCM differentially de-methylated genes-Biological Process. This file contains the list of GO terms in the category of biological process that were significantly (p<0.05) overrepresented among the 195 hCM differentially demethylatedgenes [file 13238_2013_16_MOESM20_ESM.pdf]

**Genes**	**Sequences**
ACTA2-qpcr-F	GTGTTGCCCCTGAAGAGCAT
ACTA2-qpcr-R	GCTGGGACATTGAAAGTCTCA
CASQ2-qpcr-F	GGCAGAAGAGGGGCTTAATTT
CASQ2-qpcr-R	GAAGACACCGGCTCATGGTAG
CREM-qpcr-F	ACACCACCTAGTATTGCTACCA
CREM-qpcr-R	GGATTGTTCCACCTTGGGCTAT
CSRP3-qpcr-F	CCTGTGAAAAGACCGTCTACC
CSRP3-qpcr-R	GTCGTGCTGTCAAGAGCCT
DTNA-qpcr-F	TACCCACGGAAGTTTTGGAGG
DTNA-qpcr-R	GGATCTGACATAAGCGTGTCC
EYA4-qpcr-F	CTTCTTGCAGTCAAAACAGAGC
EYA4-qpcr-R	GTGGATAGGGCTTGGAAGGAT
FBXO32-qpcr-F	GCCTTTGTGCCTACAACTGAA
FBXO32-qpcr-R	CTGCCCTTTGTCTGACAGAAT
GATA4-qpcr-F	CGACACCCCAATCTCGATATG
GATA4-qpcr-R	GTTGCACAGATAGTGACCCGT
GATA5-qpcr-F	CTTCGTGTCCGACTTCTTGGA
GATA5-qpcr-R	CCGAGGCATTCCTTGTGGA
GATA6-qpcr-F	CTCAGTTCCTACGCTTCGCAT
GATA6-qpcr-R	GTCGAGGTCAGTGAACAGCA
HAND2-qpcr-F	CGCCGACACCAAACTCTCC
HAND2-qpcr-R	TCGCCATTCTGGTCGTCCT
HECW2-qpcr-F	AAATCCCCAGATGCGGTACAC
HECW2-qpcr-R	CGGCTCTCAGAAGTCACCA
ISL1-qpcr-F	GCGGAGTGTAATCAGTATTTGGA
ISL1-qpcr-R	GCATTTGATCCCGTACAACCT
KCNA5-qpcr-F	CGCGTCCACATCAACATCTC
KCNA5-qpcr-R	GGTAGAAGCGTATCTCGTCCG
LBH-qpcr-F	GCCCCGACTATCTGAGATCG
LBH-qpcr-R	GCGGTCAAAATCTGACGGGT
LDB3-qpcr-F	CTATCTCCCGGATCACACCAG
LDB3-qpcr-R	GTGAGGCTCAAGTTGTAGCTG
LEFTY2-qpcr-F	TGGACCTCAGGGACTATGGAG
LEFTY2-qpcr-R	CCGAGGCGATACACTGTCG
MARCH11-qpcr-F	CCGGGGAGAGTCTTCCACA
MARCH11-qpcr-R	GCCTCATCCGATTGAACAGGT
MEF2A-qpcr-F	ACTACAGACCTCACAGTGCCA
MEF2A-qpcr-R	GCCTAAGCTATTTGCACCAGT
MEF2C-qpcr-F	CCAACTTCGAGATGCCAGTCT
MEF2C-qpcr-R	GTCGATGTGTTACACCAGGAG
MIR21-qpcr-F	CTGCCTGACTGTCTGCTTGTT

MIR21-qpcr-R	AGATTCAACAGTCAACATCAGTCTGA
MITF-qpcr-F	CAGTCCGAATCGGGGATCG
MITF-qpcr-R	TGCTCTTCAGCGGTTGACTTT
MYBPC3-qpcr-F	CCATGACGCTGAGGTCAAATG
MYBPC3-qpcr-R	GCTTGGCACCGATGGACTC
MYOCD-qpcr-F	ACGGATGCTTTTGCCTTTGAA
MYOCD-qpcr-R	AACCTGTCGAAGGGGTATCTG
MYOZ2-qpcr-F	CCAGGCTATTTAAGATGCGTCA
MYOZ2-qpcr-R	CCTTCCAAGTTACTTCCATCCAC
NEXN-qpcr-F	ACTGTGAAGGGTAGATTTGCTG
NEXN-qpcr-R	TTCTGCGTTTTCGTTCCTCCT
NKX2-5-qpcr-F	CCAAGGACCCTAGAGCCGAA
NKX2-5-qpcr-R	ATAGGCGGGGTAGGCGTTAT
NPPA-qpcr-F	CAACGCAGACCTGATGGATTT
NPPA-qpcr-R	AGCCCCCGCTTCTTCATTC
NPPB-qpcr-F	TGGAAACGTCCGGGTTACAG
NPPB-qpcr-R	CTGATCCGGTCCATCTTCCT
PCGF5-qpcr-F	AGCCAACAACAGTGACGGAAT
PCGF5-qpcr-R	TGAACTTGGTTGCCACACCTT
PLN-qpcr-F	ACCTCACTCGCTCAGCTATAA
PLN-qpcr-R	CATCACGATGATACAGATCAGCA
PPAPDC3-qpcr-F	AGCTGAACCCCTCCTTCAAG
PPAPDC3-qpcr-R	CCGATGACAAAGCCGGAGA
PPARGC1A-qpcr-F	TCTGAGTCTGTATGGAGTGACAT
PPARGC1A-qpcr-R	CCAAGTCGTTCACATCTAGTTCA
PRICKLE1-qpcr-F	TTTGCTTGCTTACCAGAGGAAA
PRICKLE1-qpcr-R	ACTGGCAATACCGTACCTCAT
RARB-qpcr-F	TCCGAAAAGCTCACCAGGAAA
RARB-qpcr-R	GGCCAGTTCACTGAATTTGTCC
SMAD6-qpcr-F	GCTACCAACTCCCTCATCACT
SMAD6-qpcr-R	CGTACACCGCATAGAGGCG
SMAD7-qpcr-F	TTCCTCCGCTGAAACAGGG
SMAD7-qpcr-R	CCTCCCAGTATGCCACCAC
SNTA1-qpcr-F	TGCTCCTCTACTTGTCTCTCC
SNTA1-qpcr-R	TCTGCATCGTAGGGCACTGA
STAT4-qpcr-F	TGTTGGCCCAATGGATTGAAA
STAT4-qpcr-R	GGAAACACGACCTAACTGTTCAT
TBX2-qpcr-F	GCTGACGATTGCCGCTATAAG
TBX2-qpcr-R	GGCTGTCTGGGTGGATGTA
TCEA3-qpcr-F	AAGAGCACGGACATGAAGTACC
TCEA3-qpcr-R	CTCTGCCGTCATCTTGGCTA
TNNT2-qpcr-F	GGAGGAGTCCAAACCAAAGCC

TNNT2-qpcr-R	TCAAAGTCCACTCTCTCTCCATC
TRIM63-qpcr-F	CTTCCAGGCTGCAAATCCCTA
TRIM63-qpcr-R	ACACTCCGTGACGATCCATGA
TTN-qpcr-F	CCCCATCGCCCATAAGACAC
TTN-qpcr-R	CCACGTAGCCCTCTTGCTTC
XPO4-qpcr-F	ATTTCAGCGTTACCTTGCACT
XPO4-qpcr-R	CAGCACATTTTGCGAGGATTC
ZFPM2-qpcr-F	GGCCTGAAAATCTGAGCTGC
ZFPM2-qpcr-R	CAGTCGTCTGTCTCAACTCCA
